# One-pot sequential multicomponent reaction between *in situ* generated aldimines and succinaldehyde: facile synthesis of substituted pyrrole-3-carbaldehydes and applications towards medicinally important fused heterocycles†

**DOI:** 10.1039/c8ra01637b

**Published:** 2018-04-24

**Authors:** Anoop Singh, Nisar A. Mir, Sachin Choudhary, Deepika Singh, Preetika Sharma, Rajni Kant, Indresh Kumar

**Affiliations:** Department of Chemistry, Birla Institute of Technology and Science Pilani 333 031 Rajasthan India indresh.chemistry@gmail.com indresh.kumar@pilani.bits-pilani.ac.in; Instrumentation Division, IIIM-CSIR Lab Jammu 180 001 India; X-ray Crystallography Laboratory, Post-Graduate Department of Physics & Electronics, University of Jammu Jammu 180 006 India; Department of Chemistry Govt. Degree College for Pulwama-192301 J&K India

## Abstract

An efficient sequential multi-component method for the synthesis of *N*-arylpyrrole-3-carbaldehydes has been developed. This reaction involved a proline-catalyzed direct Mannich reaction-cyclization sequence between succinaldehyde and *in situ* generated Ar/HetAr/indolyl-imines, followed by IBX-mediated oxidative aromatization in one-pot operation. The practical utility of this procedure is shown at gram-scale and the synthesis of diverse bioactive fused heterocyclic scaffolds such as pyrroloquinoline, pyrrolo-oxadiazole, dihydro pyrroloquinoline, and pyrrolo-phenanthridine.

## Abstract 2

An efficient sequential multi-component method for the synthesis of *N*-arylpyrrole-3-carbaldehydes has been developed.

## Graphical abstract

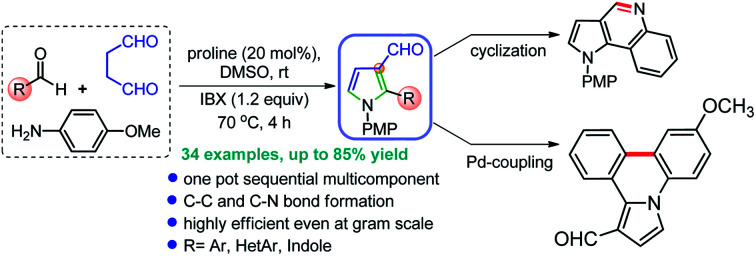

## Introduction

**Fig. 1:**
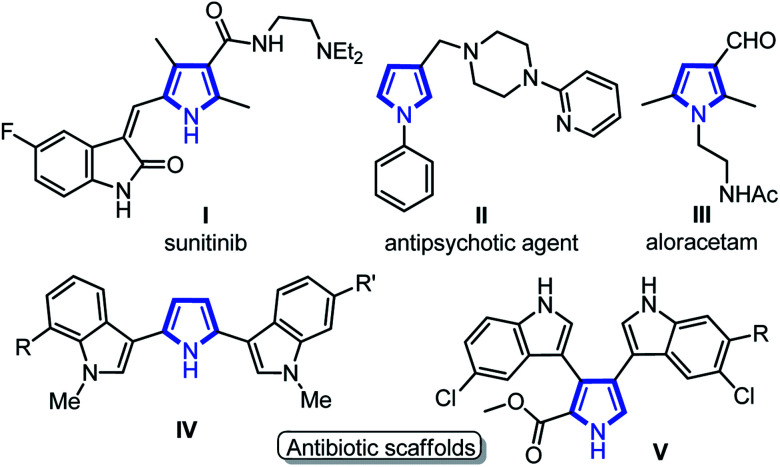
Bioactive pyrroles and related derivatives.

**Scheme 1:**
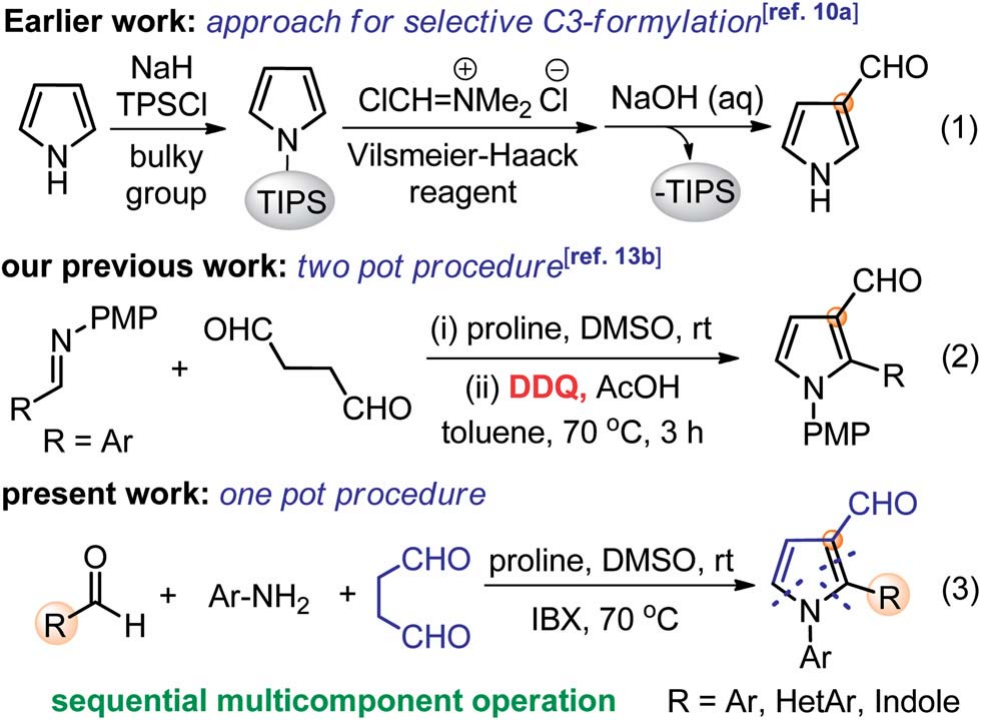
Synthetic approaches for pyrrole-3-carbaldehydes.

Medium sized nitrogen heterocycles are privileged scaffolds present in numerous natural and unnatural compounds.^1^ Among them, the pyrrole core is present in many important classes of natural products and its derivatives have been used as valuable intermediates for the synthesis of many drugs that exhibit interesting biological activities (Fig. 1).^2^ In addition, functionalized pyrroles have shown wide applications in agrochemicals and flavor components, dyes, and functionalized materials.^3^ Over the past few decades, a number of elegant methods to access functionalized pyrroles have been reported, which includes classical methods,^4^ cycloadditions,^5^ multi-component,^6^ metal-catalyzed reactions,^7^ and several others.^8^ Despite the extensive efforts, the synthesis of C3-functionalized pyrroles is probably the most difficult task and required a special strategy.^9^ In particular, the regiospecific synthesis of pyrroles endowed with aldehyde group at C3-position is still very rare.^10^ Pyrrole-3-carbaldehydes have mainly been synthesized by the use of sterically bulky triisopropylsilyl (TIPS) as protecting group on the nitrogen of pyrrole followed by Vilsmeier–Hacck formylation and deprotection as a multistep process (eqn (1), Scheme 1),^10*a*^ along with other direct/indirect methods.^10*b*–*e*^ However, these reported approaches have one or more drawbacks, such as the requirement of specially designed substrates, multistep process with low yields, and harsh reaction conditions. Furthermore, none of these methods could directly yield 1,2-disubstituted pyrrole-3-carbaldehydes, to the best of our knowledge. The development of synthetic protocol, which directly produces the required functionality at the desired position of heterocyclic ring systems, has become a major contribution to the pharmaceutical industry. Thus, the development of modular and simple pot-economic protocol to strategically access substituted pyrrole-3-carbaldehydes from easily available materials is still in high demand. Notably, aldehyde group at C3-position of pyrroles can readily participate in numerous name reactions, thus, holds significant promises to serve as a suitable intermediate to synthesize new medicinal agents and functionalized materials.^11^

In continuation to our efforts to utilize using linear dialdehydes,^12^ for the synthesis of medium-sized N-heterocycles under metal-free conditions,^13^ we recently developed a two-pot protocol for the direct synthesis of pyrrole-3-carbaldehyde from succinaldehyde and imines (eqn (2), Scheme 1).^13*b*^ This method resulted in a quick synthesis of pyrrole-3-aldehydes, though, it required the pre-assembled imines and DDQ as a harsh and toxic reagent for oxidative aromatization. The clear synthetic potential and novelty of this method to suitably functionalized pyrroles prompted us to explore similar transformation in one-pot sequential multicomponent fashion under mild conditions. In addition, the social and environmental demands for more sustainable and practical synthetic protocols that need the use of less hazardous reagents/conditions has also gained much attention of the scientific community. In this context, multicomponent reactions allow the rapid construction of novel libraries of pharmaceutically active compounds and marine alkaloids, thus, the development of such protocol is always applauded.^14^ Herein, we report a simple and most rational sequential multicomponent protocol for the synthesis of pyrrole-3-carbaldehydes *via in situ* imine formation between Ar/HetAr/indole-aldehydes and Ar-NH_2_, followed by amine-catalyzed direct Mannich reaction-cyclization with succinaldehyde, and IBX-mediated aromatization sequence in one-pot operation (eqn (3), Scheme 1). This improved method provides an easy access to pyrrole-3-carbaldehydes under mild and non-toxic condition as compare to our previous two-pot protocol. In addition, *in situ* generated imines derived from indole-3-aldehydes have been explored for the first time for such transformations to yield indole-based medicinally important scaffolds.

## Results and discussion

Based on our previous experience in this direction, we quickly optimized the designed reactions by choosing proline 1 (20 mol%) as a catalyst, *p*-nitrobenzaldehyde 2c as model substrates, along with *p*-anisidine 3, and succinaldehyde 4 (3 M aqueous sol.), in one-pot operation and the results are shown in Table 1. Initial experiments in DMSO as the choice of solvent along with the sequential addition of substrates, catalyst, and oxidant(s) gave 5c in low yield (entry 1 and 2, Table 1). Attempts were made to improve the yields by changing the solvents (entries 2–4, Table 1), however, failed. IBX as the oxidant, also soluble in DMSO, showed good efficiency for this one-pot protocol at rt (entry 5, Table 1) and at 50 °C (entry 6, Table 1). Gratifyingly, an additional improvement in yields (80%) was observed when IBX-oxidation was carried out at 70 °C for 4 h (entry 7, Table 1). However, additional efforts to increase the reaction yield by further increment in reaction temperature (entry 8, Table 1), reduction in catalyst loading (entry 9, Table 1), varying the reaction medium (entries 10 and 11, Table 1), and changing the catalytic system (entry 12, Table 1) were ineffective. Thus, we prefer to perform this one-pot sequential transformation with the optimized conditions (entry 7, Table 1).

**Table 1:** Optimization of reaction conditions^a^

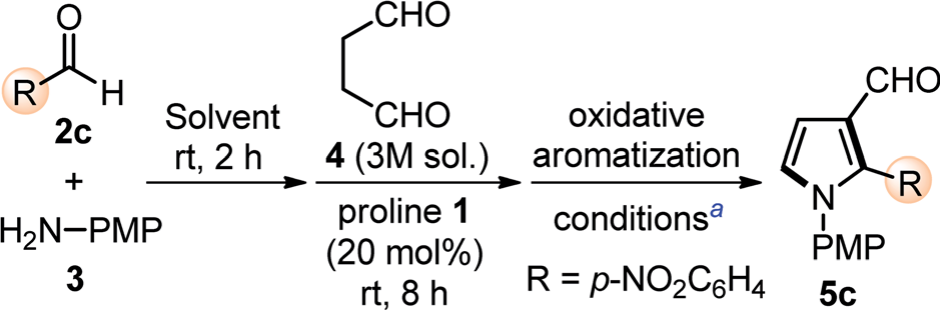			
Entry	Solvent	Conditions^a^	Yield^b^ (%)
1	DMSO	K_2_S_2_O_8_ (1.2 equiv.), rt, 8 h	35
2	DMSO	Oxone (1.2 equiv.), rt, 24 h	40
3	DMF	Oxone (1.2 equiv.), rt, 24 h	30
4	CH_3_CN	Oxone (1.2 equiv.), rt, 24 h	<20
5	DMSO	IBX (1.2 equiv.), rt, 6 h	50
6	DMSO	IBX (1.2 equiv.), 50 °C, 6 h	64
**7**	**DMSO**	**IBX (1.2 equiv.), 70** °**C, 4 h**	**80**
8	DMSO	IBX (1.2 equiv.), 90 °C, 4 h	75
9^c^	DMSO	IBX (1.2 equiv.), 70 °C, 4 h	58
10	DMF	IBX (1.2 equiv.), 70 °C, 4 h	43
11^d^	CH_3_CN	IBX (1.2 equiv.), 70 °C, 4 h	35
12^e^	DMSO	IBX (1.2 equiv.), 70 °C, 4 h	48

a Unless otherwise indicated, the reaction was carried out with (i) aldehyde 2 (0.3 mmol), *p*-anisidine 3 (0.3 mmol), succinaldehyde 4 (3 M aqueous sol., 0.9 mmol), proline 1 (20 mol%), solvent (3.0 mL); (ii) IBX (1.2 equiv.).

b Isolated yield of 5c refers to 2c.

c Proline 1 (10 mol%).

d EtOAc (3.0 mL) was added during IBX-mediated oxidative aromatization.

e Pyrrolidine (20 mol%), PhCO_2_H (20 mol%) were used in place of proline 1.

**Scheme 2:**
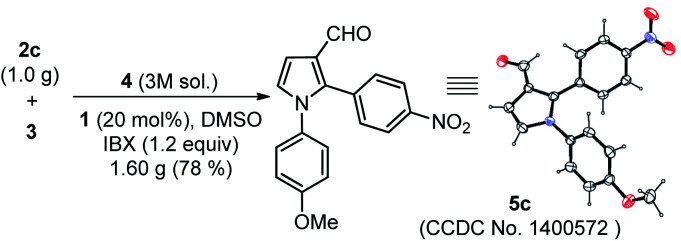
Single-crystal X-ray analysis of 5c. Thermal ellipsoids are drawn at the 40% probability level.

The scope of the reaction was examined by employing various aromatic aldehydes and the results are summarized in Table 2. This one-pot sequential multicomponent protocol works well in case of aromatic aldehydes decorated with various groups (*e.g.* –NO_2_, –F, –Cl, –Br, –CN and CF_3_) at the *ortho*-, *meta*-, or *para*-positions (entries 5a–5l, Table 2) and resulted in 2-aryl-pyrrole-3-carbaldehydes in good to high yields (65–80%). The reaction works well with *in situ* generated simple aryl imine (entry 5m, Table 2), as well as with hetero-aryl imines (entries 5n–5o, Table 2) with good yields. The feasibility of this protocol was also examined at gram scale of 2c (1.0 g) with other reactants under standardized conditions and corresponding product 5c was obtained without much variation in yields (1.60 g, 78%). The structure of 5c was further confirmed with single crystal X-ray diffraction analysis (Scheme 2).^15^

**Table 2:** Substrate scope with respect to various Ar/HetAr-aldehydes 2^a^

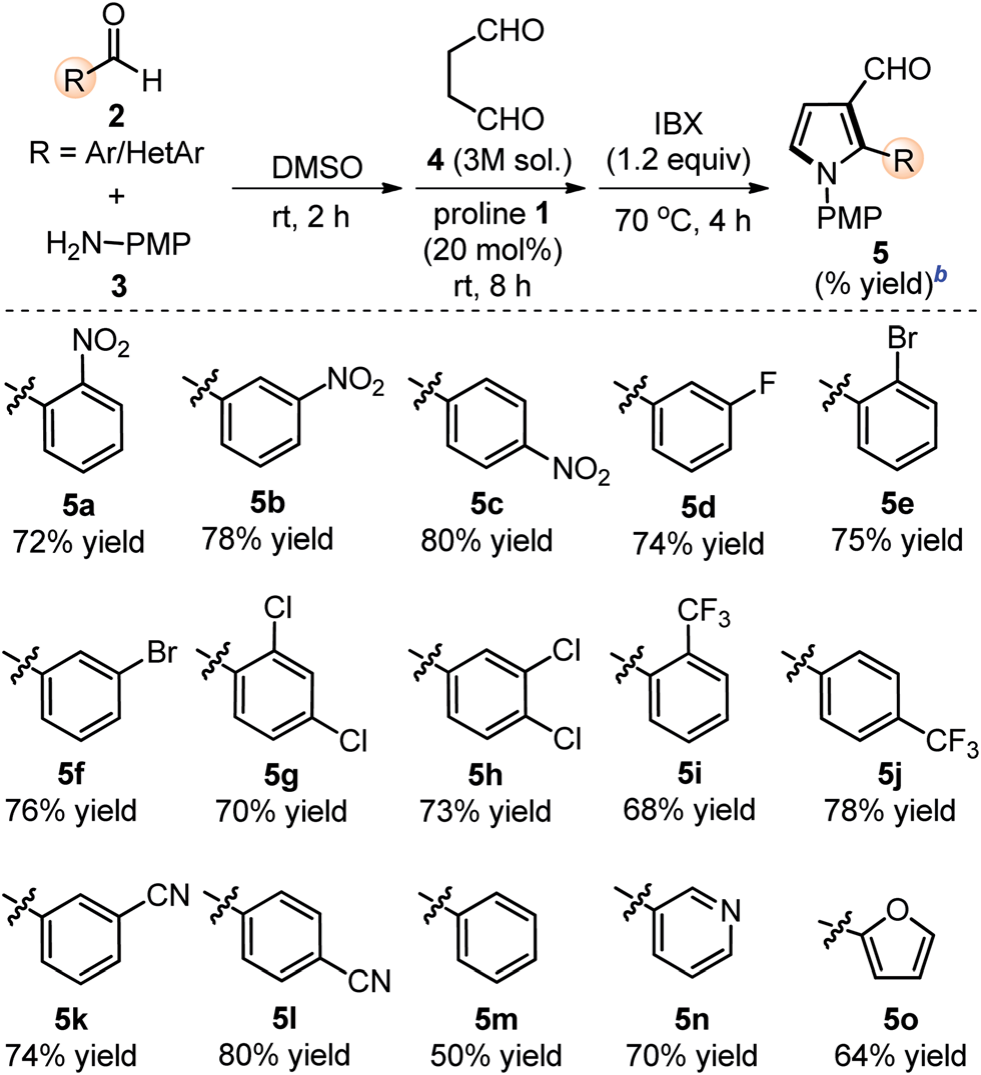

a Unless otherwise indicated, the reaction was carried out with (i) aldehyde 2 (0.3 mmol), *p*-anisidine 3 (0.3 mmol), DMSO (3.0 mL), rt, 2 h, (ii) succinaldehyde 4 (3 M aqueous sol., 0.9 mmol), proline 1 (20 mol%), 8 h, (iii) IBX (1.2 equiv.), 70 °C, 4 h.

b Isolated yield of 5 refers to 2 (≤10% of aldehyde 2 was recovered in all the cases).

### Indolyl-pyrroles synthesis

**Fig. 2:**
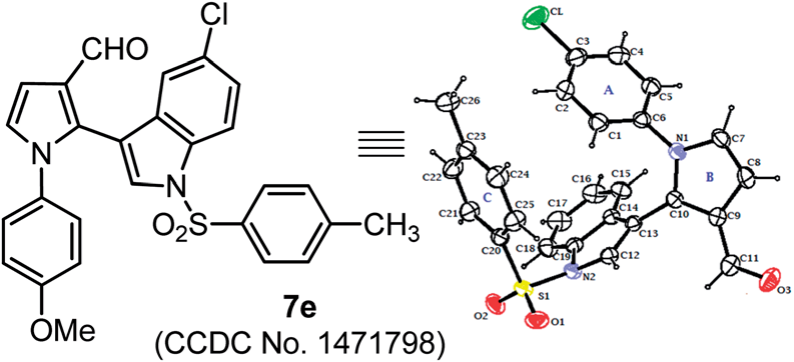
Single-crystal X-ray analysis of 7e. Thermal ellipsoids are drawn at the 40% probability level.

**Scheme 3:**
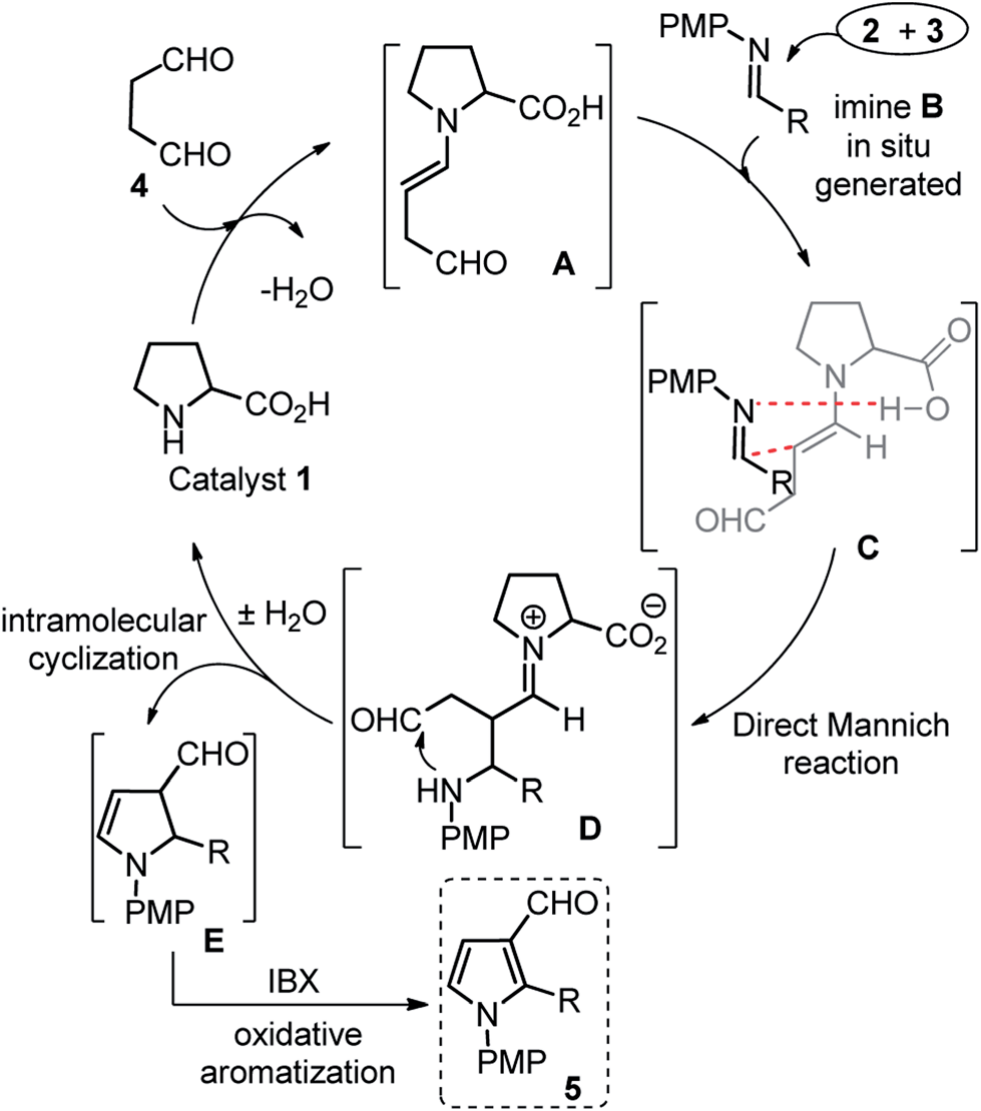
Proposed reaction mechanism for this study.

The scope of this one-pot protocol was further was examined by employing various *in situ* generated imines derived from indole-3-aldehydes 6. This extension to imines derived from indole-3-aldehydes could be motivating as these units have not been utilized for similar direct Mannich reaction and can lead to new structural scaffolds. Indole and its derivatives are an important family of alkaloid compounds and most abundant heterocycles found in nature, which possess interesting biological activities.^16^ Moreover, indole-tethered pyrrole derivatives (for example IV and V in Fig. 1) found in several synthetic compounds and marine alkaloids that showed remarkable bioactivities,^17^ therefore, the synthesis of these compounds is quite interesting. In this context, a series of indolyl-pyrrole-3-carbaldehydes 7a–7p were obtained with moderate to good yields, when *N*-Ts, Ms, SO_2_Ph, Boc protected indole-3-aldehydes 6 were employed for this transformation with *p*-anisidine 3 and succinaldehyde 4 (entries 7a–7p, Table 3). In addition, electron donating or withdrawing substitution on indole-ring did not alter the course of this transformation. Further, the reaction works quite well when other aryl-amines such as 2-aminophenol and 4-chloroaniline (entries 7q–7r, Table 3) were employed instead of *p*-anisidine 3 for this one-pot transformation with *N*-Ts-indol-3-aldehyde 6a and succinaldehyde 4. However, reaction failed when the similar transformation was performed with *N*-benzyl-indole-3-aldehyde, probably because of low reactivity of imine (entry 7s, Table 3). All compounds were well characterized by ^1^H and ^13^C-NMR and mass-analysis. Single crystal X-ray diffraction analysis of 7e further established the product structure (Fig. 2).^15^ Based on our study, a stepwise mechanism is proposed to account for this reaction. As shown in Scheme 3, the enamine A*in situ* generated from succinaldehyde 4 and catalyst 1, reacts with *in situ* generated *N*-PMP-imine B*via* a direct Mannich reaction model C to produced Mannich product D. The intermediate D underwent intramolecular cyclization to dihydropyrrole E with the simultaneous release of catalyst 1. In the same pot cyclic enamine-intermediate E underwent IBX-mediated oxidative aromatization to afford pyrrole-3-carboxaldehyde 4.

**Table 3:** Substrate scope with respect to various indole-3-aldehydes 6^a^

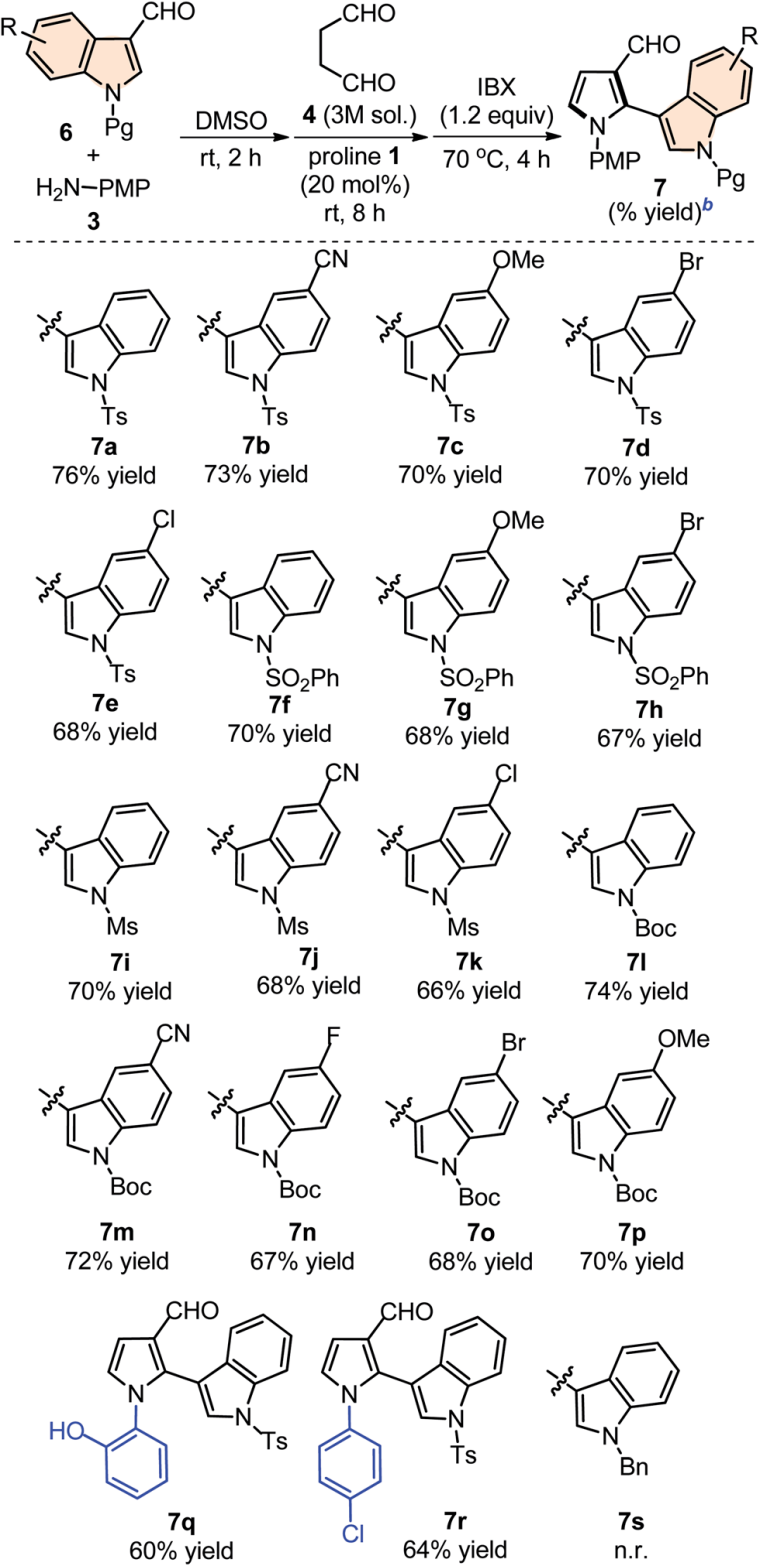

a Unless otherwise indicated, the reaction was carried out with (i) aldehyde 6 (0.3 mmol), *p*-anisidine 3 (0.3 mmol), DMSO (3.0 mL), rt, 2 h, (ii) succinaldehyde 4 (3 M aqueous sol., 0.9 mmol), proline 1 (20 mol%), 8 h, (iii) IBX (1.2 equiv.), 70 °C, 4 h.

b Isolated yield refers to 6 (≤10% of aldehyde 6 was recovered in all the cases).

### Synthetic applications

**Scheme 4:**
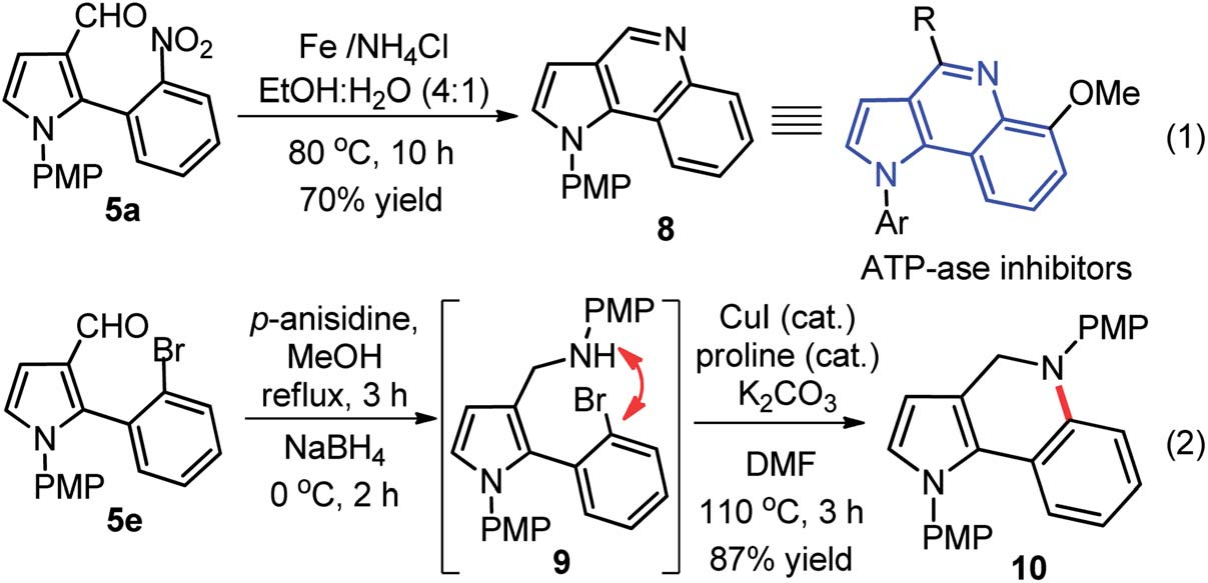
Synthesis of pyrroloquinoline 8 and pyrrolo-dihydroquinoline 10 scaffolds.

Substituted pyrrole-3-aldehydes could participate as suitable intermediates for further functionalization to many important and complex scaffolds, therefore, we developed interesting and useful synthetic applications of these compounds. In this context, a rapid synthesis of pyrrolo[3,2-*c*]quinoline 8 was developed through reductive cyclization. This reaction proceeded *via in situ* amine formation through reduction of nitro-group of 5a Fe/NH_4_Cl in EtOH : H_2_O (4 : 1), which underwent intramolecular cyclization with aldehyde group in the same pot with good yields (eqn (1), Scheme 4). In another approach, the synthesis of pyrrole-dihydroquinoline 10 was accomplished through the reductive amination of 5e with *p*-anisidine 3 in presence of NaBH_4_ to generate *in situ* amine 9, which was further utilized for CuI-catalyzed intramolecular coupling (C–N) without purification to furnished 10 with high yield (87%) over two steps (eqn (2), Scheme 4).^18^ The pyrroloquinoline moiety was found to be present in many natural/synthetic molecule with interesting bioactivity and our protocol may be better alternative to the previous procedure.^19^ The synthesized hybrid scaffolds resemble with various biologically active molecules such as pyrrolo[3,2-*c*]-quinoline derivative, an ATP-ase inhibitor,^20*a*^ pyrrolo[2,3-*c*]-quinoline derivative, a natural product with acetylcholinesterase-inhibiting activity,^20*b*^ and pyrrolo[3,4-*c*]quinoline derivative, a potent 5-HT4R antagonist with analgesic action.^20*c*^

**Scheme 5:**
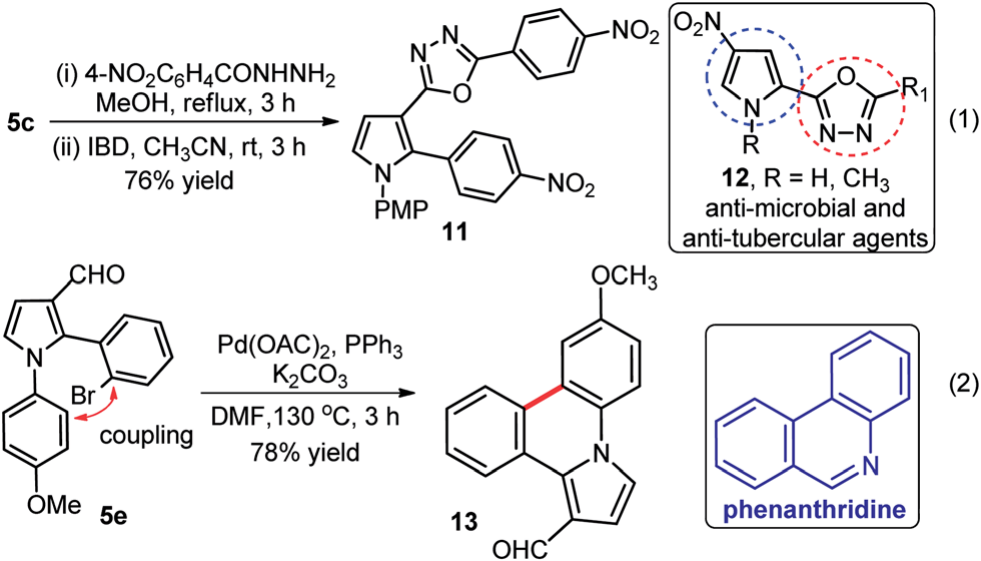
Synthesis of pyrrole-oxadiazole 11, and pyrrole-phenanthridine 13 moieties.

Further applications of our method were shown; (i) synthesis of pyrrole-oxadiazole 11 in good yield (76%) over two steps from compound 5c which was initially condensed with 4-nitrophenylhydrazide, followed by iodobenzene diacetate (IBD) mediated oxidative cyclization under the standardized conditions (eqn (1), Scheme 5),^21^ and (ii) rapid and high yielding (78%) synthesis of pyrrole-phenanthridine 13 from 5e through intramolecular C–C bond formation in presence Pd(OAc)_2_, PPh_3_ and K_2_CO_3_ in DMF at 130 °C as shown in (eqn (2), Scheme 5). Interestingly, 13 might exhibit interesting biological activities because phenanthridines serve as the core structure of natural products from Amaryllidaceae plants and received considerable attention from both chemists and biological scientists.^22^

## Conclusions

In summary, we have developed a straight forwarded sequential multicomponent synthesis of substituted *N*-aryl-pyrrole-3-carbaldehydes. This one-pot protocol proceeds through proline-catalyzed Mannich reaction-cyclization sequence between succinaldehyde and imines, *in situ* generated from Ar/HetAr/indole-aldehydes with aromatic amines, followed by IBX-mediated oxidative aromatization under mild conditions. Easy access to the starting materials and direct synthesis of pyrrole-3-carbaldehydes under metal-free conditions renders this method potentially useful in organic synthesis. Synthetic applicability of the developed method was established through; (i) at gram-scale synthesis, and (ii) the rapid access to the biologically important natural products analogous like-pyrrolo-quinoline, pyrrolo-oxadiazole, dihydro pyrroloquinoline, and pyrrolo-phenanthridine.

## Experimental

### General remarks

Unless otherwise stated, all reagents were purchased from commercial suppliers and used without further purification. All solvents employed in the reactions were distilled from appropriate drying agents prior to use. All reactions under standard conditions were monitored by thin-layer chromatography (TLC) on Merck silica gel 60 F254 pre-coated plates (0.25 mm). The column chromatography was performed on silica gel (100–200) using a mixture of hexane/EtOAc. Chemical yields refer to pure isolated substances. ^1^H-NMR spectra were recorded on a BRUKER-AV400 (400 MHz) spectrometer. Chemical shifts are reported in ppm from tetramethylsilane with the solvent resonance as the internal standard (CDCl_3_ = *δ* 7.26 for ^1^H, and 77.0 for ^13^C-NMR). Data are reported as follows: chemical shift, multiplicity (s = singlet, d = doublet, dd = doublet of doublet, t = triplet, q = quartet, br = broad, m = multiplet), coupling constants (Hz) and integration. ^13^C-NMR spectra were recorded on a BRUKER-AV400 (75 MHz) spectrometer with complete proton decoupling. HRMS were performed employing an ESI+ ionization method and TOF as an analyzer. Infrared (FT-IR) spectra were recorded on an ABB Bomen MB 3000 FTIR Spectrophotometer system using KBr pellets. Melting points were determined in open capillary tubes with an EZ-Melt automated melting point apparatus and may be incorrect.

### Typical procedure for the synthesis of pyrrole-3-carboxaldehydes

To a stirred solution of Ar/HetAr-aldehyde 2 (0.3 mmol) or N-protected indole-3-aldehyde 6 (0.3 mmol) in DMSO (3.0 mL) was added *p*-anisidine 3 (0.3 mmol) and stirred initially for 2 h at rt. To this *in situ* generated-imine solution was added succinaldehyde 4 (0.3 mL, 0.9 mmol, 3 M solution) and proline 1 (7.0 mg, 0.06 mmol) at the same temperature. The combined reaction mixture was stirred further for 8 h at rt. At that time, IBX (100 mg, 0.36 mmol, 1.2 equiv.) was added to the reaction mixture and heated at 70 °C for additional 3 h. The reaction was cooled to room temperature quenched with NaHCO_3_ solution (10% solution, 5 mL) and extracted with EtOAc (3 × 6 mL). The combined organic extracts were washed with brine, dried over Na_2_SO_4_ anhydrous, and concentrated under reduced pressure. Purification through silica gel column chromatography by eluting the mixture of hexane/EtOAc, gave pyrrole-3-carbaldehydes 5 or 7 with 50–80% yields. In almost all the cases, we also obtained about <10% initial starting aldehyde due to cleavage of corresponding imine under these conditions.

#### 1-(4-Methoxyphenyl)-2-(2-nitrophenyl)-1*H*-pyrrole-3-carbaldehyde (5a)

Reddish pasty liquid (70 mg, 72%); ^1^H NMR (400 MHz, CDCl_3_) *δ* 3.77 (s, 3H), 6.77 (d, *J* = 8.9 Hz, 2H), 6.87 (d, *J* = 3.0 Hz, 1H), 6.94 (d, *J* = 3.0 Hz, 1H), 7.02 (d, *J* = 8.9 Hz, 2H), 7.45 (d, *J* = 6.5 Hz, 1H), 7.54 (t, *J* = 7.8 Hz, 1H), 7.62 (t, *J* = 6.6 Hz, 1H), 7.97 (d, *J* = 8.1 Hz, 1H), 9.60 (s, 1H); ^13^C NMR (75 MHz, CDCl_3_) *δ* 55.4, 109.1, 114.3 (2C), 124.4, 124.8, 125.1, 125.2, 126.9 (2C), 130.1, 130.8, 132.7, 134.1, 135.8, 149.3, 159.1, 185.5; IR (KBr)/cm^−1^, 2932, 1666, 1520, 1350, 1296, 1034; HRMS (ESI): calcd for C_18_H_14_N_2_O_4_ (MH^+^) 323.1032; found 323.1033.

#### 1-(4-Methoxyphenyl)-2-(3-nitrophenyl)-1*H*-pyrrole-3-carbaldehyde (5b)

Semi-solid (75 mg, 78%); ^1^H NMR (400 MHz, CDCl_3_) *δ* 3.73 (s, 3H), 6.77 (d, *J* = 8.8 Hz, 2H), 6.82 (d, *J* = 3.0 Hz, 1H), 6.87 (d, *J* = 3.0 Hz, 1H), 6.97 (d, *J* = 8.0 Hz, 2H), 7.41–7.49 (m, 2H), 8.01 (t, *J* = 1.4 Hz, 1H), 8.11 (d, *J* = 8.0 Hz, 1H), 9.65 (s, 1H); ^13^C NMR (75 MHz, CDCl_3_) *δ* 55.5, 108.8, 114.6 (2C), 123.2, 124.9, 125.5, 125.8, 127.3 (2C), 129.2, 130.8, 131.1, 136.6, 138.2, 147.9, 159.3, 186.0; IR (KBr)/cm^−1^ 2920, 1746, 1680, 1244, 1172; HRMS (ESI): calcd for C_18_H_14_N_2_O_4_ (MH^+^) 323.1032; found 323.1033.

#### 1-(4-Methoxyphenyl)-2-(4-nitrophenyl)-1*H*-pyrrole-3-carbaldehyde (5c)

Pale yellow solid (mp = 102–104 °C) (74 mg, 80%); ^1^H NMR (400 MHz, CDCl_3_) *δ* 3.82 (s, 3H), 6.84 (d, *J* = 8.8 Hz, 2H), 6.89 (d, *J* = 3.0 Hz, 1H), 6.94 (d, *J* = 3.0 Hz, 1H), 7.01 (d, *J* = 8.8 Hz, 2H), 7.35 (d, *J* = 8.8 Hz, 2H), 8.16 (d, *J* = 8.8 Hz, 2H), 9.73 (s, 1H); ^13^C NMR (75 MHz, CDCl_3_) *δ* 55.4, 109.0, 114.6 (2C), 123.4 (2C), 125.1, 126.1, 127.1 (2C), 130.9, 131.6 (2C), 135.9, 138.2, 147.4, 159.3, 186.0; IR (KBr)/cm^−1^ 2933, 1724, 1660, 1249, 1174; HRMS (ESI): calcd for C_18_H_14_N_2_O_4_ (MH^+^) 323.1032; found 323.1028.

#### 2-(3-Fluorophenyl)-1-(4-methoxyphenyl)-1*H*-pyrrole-3-carbaldehyde (5d)

Yellow pasty liquid (65 mg, 74%); ^1^H NMR (400 MHz, CDCl_3_) *δ* 3.80 (s, 3H), 6.83 (d, *J* = 8.9 Hz, 2H), 6.86 (d, *J* = 3.1 Hz, 1H), 6.89 (d, *J* = 2.8 Hz, 1H), 6.91 (t, *J* = 1.8 Hz, 1H), 7.00–7.06 (m, 4H), 7.28–7.32 (m, 1H), 9.70 (s, 1H); ^13^C NMR (75 MHz, CDCl_3_) *δ* 55.4, 107.8, 114.3 (2C), 115.7, 117.9, 124.6, 125.2, 126.8, 127.0, (2C), 129.8, 129.9, 131.3, 140.4, 158.9, 161.2, 186.6; IR (KBr)/cm^−1^, 2962, 1720, 1512, 1247, 1172; HRMS (ESI): calcd for C_18_H_14_FNO_2_ (MH^+^) 296.1087; found 296.1070.

#### 2-(2-Bromophenyl)-1-(4-methoxyphenyl)-1*H*-pyrrole-3-carbaldehyde (5e)

Red viscous liquid (80 mg, 75%); ^1^H NMR (400 MHz, CDCl_3_) *δ* 3.77 (s, 3H), 6.78 (d, *J* = 8.9 Hz, 2H), 6.86 (d, *J* = 3.1 Hz, 1H), 6.92 (d, *J* = 3.0 Hz, 1H), 6.95 (d, *J* = 8.9 Hz, 2H), 6.98–7.00 (m, 1H), 7.56 (dd, *J* = 7.9, 4.3 Hz, 2H), 7.64 (d, *J* = 8.3 Hz, 1H), 9.51 (s, 1H); ^13^C NMR (75 MHz, CDCl_3_) *δ* 55.4, 107.9, 114.2 (2C), 124.9, 125.1, 126.5 (2C), 126.9, 127.7, 129.7, 131.3, 134.0, 135.9, 136.1, 137.2, 158.9, 185.9; IR (KBr)/cm^−1^, 3016, 1720, 1519, 1226, 1026; HRMS (ESI): calcd for C_18_H_14_BrNO_2_ (MH^+^) 356.0286; found 356.0295.

#### 2-(3-Bromophenyl)-1-(4-methoxyphenyl)-1*H*-pyrrole-3-carbaldehyde (5f)

Brown pasty liquid (81 mg, 76%); ^1^H NMR (400 MHz, CDCl_3_) *δ* 3.81 (s, 3H), 6.84 (d, *J* = 2.1 Hz, 1H), 6.86 (d, *J* = 4.1 Hz, 2H), 6.89 (d, *J* = 3.0 Hz, 1H), 7.03 (d, *J* = 7.0 Hz, 2H), 7.08–7.11 (m, 1H), 7.17 (t, *J* = 7.8 Hz, 1H) 7.41 (t, *J* = 1.7 Hz, 1H) 7.45–7.48 (m, 1H), 9.69 (s, 1H); ^13^C NMR (75 MHz, CDCl_3_) *δ* 55.5, 108.0, 114.4 (2C), 122.2, 122.8, 124.7, 125.3, 127.1 (2C), 129.5, 129.7, 131.3, 131.6, 133.7, 137.2, 159.0, 186.5; IR (KBr)/cm^−1^, 2985, 1728, 1519, 1373, 1242, 1049; HRMS (ESI): calcd for C_18_H_14_BrNO_2_ (MH^+^) 356.0286; found 356.0288.

#### 2-(2,4-Dichlorophenyl)-1-(4-methoxyphenyl)-1*H*-pyrrole-3-carbaldehyde (5g)

Yellow oily liquid (77 mg, 70%); ^1^H NMR (400 MHz, CDCl_3_) *δ* 3.78 (s, 3H), 6.80 (d, *J* = 8.9 Hz, 2H), 6.86 (d, *J* = 3.1 Hz, 1H), 6.94 (d, *J* = 2.9 Hz, 1H), 7.03 (d, *J* = 8.9 Hz, 2H), 7.24 (d, *J* = 1.7 Hz, 2H),7.40 (s, 1H), 9.53 (s, 1H); ^13^C NMR (75 MHz, CDCl_3_) *δ* 55.4, 107.9, 114.2 (2C), 124.9, 125.1, 126.5 (2C), 126.9, 127.7, 129.7, 131.3, 134.0, 135.9, 136.1, 137.2, 158.9, 185.9; IR (KBr)/cm^−1^, 2954, 1668, 1514, 1469, 1246, 1031; HRMS (ESI): calcd for C_18_H_13_Cl_2_NO_2_ (MH^+^) 346.0401; found 346.0408.

#### 2-(3,4-Dichlorophenyl)-1-(4-methoxyphenyl)-1*H*-pyrrole-3-carbaldehyde (5h)

Yellow pasty liquid (80 mg, 73%); ^1^H NMR (400 MHz, CDCl_3_) *δ* 3.70 (s, 3H), 6.74 (d, *J* = 3.7 Hz, 2H), 6.75 (s, 1H), 6.78 (d, *J* = 3.1 Hz, 1H), 6.86–6.92 (m, 3H), 7.14 (s, 1H), 7.23 (t, *J* = 2.2 Hz, 2.0 Hz, 1H), 9.58 (s, 1H); ^13^C NMR (75 MHz, CDCl_3_) *δ* 55.4, 108.2, 114.5 (2C), 124.7, 125.5, 127.1 (2C), 129.3, 129.9, 130.2, 131.0, 132.5, 132.5, 133.0, 138.8, 159.1, 186.2; IR (KBr)/cm^−1^, 2962, 1697, 1514, 1253, 1031; HRMS (ESI): calcd for C_18_H_13_Cl_2_NO_2_ (MH^+^) 346.0401; found 346.0406.

#### 1-(4-Methoxyphenyl)-2-(2-(trifluoromethyl)phenyl)-1*H*-pyrrole-3-carbaldehyde (5i)

Yellow pasty liquid (75 mg, 68%); ^1^H NMR (400 MHz, CDCl_3_) *δ* 3.75 (s, 3H), 6.76 (d, *J* = 8.9 Hz, 2H), 6.85 (d, *J* = 3.1 Hz, 1H), 6.91 (d, *J* = 3.0 Hz, 1H), 7.03 (d, *J* = 8.8 Hz, 2H), 7.37 (t, *J* = 4.5 Hz, 1H), 7.51 (t, *J* = 4.9 Hz, 2H), 7.70 (t, *J* = 4.6 Hz, 1H), 9.41 (s, 1H); ^13^C NMR (75 MHz, CDCl_3_) *δ* 55.3, 107.5, 114.1 (2C), 114.6, 117.6, 120.1, 121.5, 122.0, 124.8, 126.5, 127.0 (2C), 129.5, 131.1, 131.4, 134.0, 158.9, 186.0; IR (KBr)/cm^−1^, 2955, 1666, 1520, 1311, 1250, 1119; HRMS (ESI): calcd for C_19_H_14_F_3_NO_2_ (MH^+^) 346.1055; found 346.1057.

#### 1-(4-Methoxyphenyl)-2-(4-(trifluoromethyl)phenyl)-1*H*-pyrrole-3-carbaldehyde (5j)

Yellow pasty liquid (85 mg, 78%); ^1^H NMR (400 MHz, CDCl_3_) *δ* 3.81 (s, 3H), 6.84 (d, *J* = 9.0 Hz, 2H), 6.89 (d, *J* = 3.1 Hz, 1H), 6.92 (d, *J* = 3.0 Hz, 1H), 7.02 (d, *J* = 8.9 Hz, 2H), 7.33 (d, *J* = 8.1 Hz, 2H), 7.58 (d, *J* = 8.1 Hz, 2H), 9.70 (s, 1H); ^13^C NMR (75 MHz, CDCl_3_) *δ* 55.4, 108.3, 114.5 (2C), 124.9, 125.1, 125.2 (3C), 125.6, 127.1 (2C), 131.1 (2C), 131.2 (2C), 133.0, 159.1, 186.4; IR (KBr)/cm^−1^, 2970, 1666, 1512, 1319, 1234; HRMS (ESI): calcd for C_19_H_14_F_3_NO_2_ (MH^+^) 346.1055; found 346.1053.

#### 3-(3-Formyl-1-(4-methoxyphenyl)-1*H*-pyrrol-2-yl) benzonitrile (5k)

Yellow pasty liquid (67 mg, 74%); ^1^H NMR (400 MHz, CDCl_3_) *δ* 3.81 (s, 3H), 6.84 (d, *J* = 8.8 Hz, 2H), 6.88 (d, *J* = 3.1 Hz, 1H), 6.92 (d, *J* = 3.0 Hz, 1H), 7.01 (d, *J* = 8.9 Hz, 2H), 7.42–7.49 (m, 3H), 7.41 (d, *J* = 7.0 Hz, 1H) 9.69 (s, 1H); ^13^C NMR (75 MHz, CDCl_3_) *δ* 55.4, 108.6, 112.6, 114.6 (2C), 118.0, 124.8, 125.7, 127.1 (2C), 129.1, 130.8, 130.9, 131.9, 134.0, 135.1, 138.4, 159.2, 186.0; IR (KBr)/cm^−1^, 2932, 2230, 1659, 1512, 1443, 1250; HRMS (ESI): calcd for C_19_H_14_N_2_O_2_ (MH^+^) 303.1134; found 303.1134.

#### 4-(3-Formyl-1-(4-methoxyphenyl)-1*H*-pyrrol-2-yl) benzonitrile (5l)

Pink pasty liquid (72 mg, 80%); ^1^H NMR (400 MHz, CDCl_3_) *δ* 3.81 (s, 3H), 6.85 (d, *J* = 8.9 Hz, 2H), 6.89 (d, *J* = 3.0 Hz, 1H), 6.94 (d, *J* = 2.5 Hz, 1H), 7.01 (d, *J* = 8.8 Hz, 2H), 7.32 (d, *J* = 8.3 Hz, 2H), 7.60 (d, *J* = 8.3 Hz, 2H), 9.72 (s, 1H); ^13^C NMR (75 MHz, CDCl_3_) *δ* 55.5, 108.9, 114.6 (2C), 118.2, 124.9, 125.0, 125.9, 127.1 (2C), 131.4 (2C), 131.9 (2C), 134.1, 138.8, 141.7, 159.3, 186.0; IR (KBr)/cm^−1^, 2962, 2229, 1712, 1519, 1242; HRMS (ESI): calcd for C_19_H_14_N_2_O_2_ (MH^+^) 303.1134; found 303.1134.

#### 1-(4-Methoxyphenyl)-2-phenyl-1*H*-pyrrole-3-carbaldehyde (5m)

Yellow pasty liquid (42 mg, 50%); ^1^H NMR (400 MHz, CDCl_3_) *δ* 3.77 (s, 3H), 6.80 (d, *J* = 8.8 Hz, 2H), 6.85 (d, *J* = 3.2 Hz, 1H), 6.87 (d, *J* = 3.2 Hz, 1H), 7.00 (d, *J* = 8.8 Hz, 2H), 7.18–7.20 (m, 2H), 7.28–7.32 (m, 3H), 9.67 (s, 1H); ^13^C NMR (75 MHz, CDCl_3_) *δ* 55.3, 107.6, 114.2 (2C), 124.3, 124.9, 127.0 (2C), 128.1 (2C), 128.4, 129.1, 130.9 (2C), 131.6, 142.4, 158.7, 187.0; IR (KBr)/cm^−1^, 2912, 1710, 1672, 1244, 1174; HRMS (ESI): calcd for C_18_H_15_NO_2_ (MH^+^) 278.1181; found 278.1189.

#### 1-(4-Methoxyphenyl)-2-(pyridin-3-yl)-1*H*-pyrrole-3-carbaldehyde (5n)

Red oily liquid (58 mg, 70%); ^1^H NMR (400 MHz, CDCl_3_) *δ* 3.81 (s, 3H), 6.85 (d, *J* = 8.9 Hz, 2H), 6.91 (d, *J* = 3.0 Hz, 1H), 6.96 (d, *J* = 2.9 Hz, 1H), 7.04 (d, *J* = 8.9 Hz, 2H), 7.28 (t, *J* = 3.7 Hz, 1H), 7.54 (d, *J* = 7.8 Hz, 1H), 8.5 (bs, 1H), 8.58 (d, *J* = 4.7 Hz, 1H), 9.72 (s, 1H); ^13^C NMR (75 MHz, CDCl_3_) *δ* 55.4, 108.4, 114.5 (2C), 122.9, 125.1, 125.7, 125.7, 127.3 (2C), 131.0, 137.8, 137.9, 149.4, 151.0, 159.2, 186.0; IR (KBr)/cm^−1^, 2954, 1666, 1512, 1242, 1033; HRMS (ESI): calcd for C_17_H_14_N_2_O_2_ (MH^+^) 279.1133; found 279.1140.

#### 2-(Furan-2-yl)-1-(4-methoxyphenyl)-1*H*-pyrrole-3-carbaldehyde (5o)

Red oily liquid (51 mg, 64%); ^1^H NMR (400 MHz, CDCl_3_) *δ* 3.85 (s, 3H), 6.05 (d, *J* = 3.3 Hz, 1H), 6.37 (d, *J* = 3.2 Hz, 1H), 6.93 (d, *J* = 8.8 Hz, 2H), 7.18 (d, *J* = 8.8 Hz, 3H), 7.99 (d, *J* = 7.7 Hz, 1H), 8.05 (d, *J* = 7.9 Hz, 1H), 10.08 (s, 1H); ^13^C NMR (75 MHz, CDCl_3_) *δ* 55.5, 108.1, 111.2, 111.7, 114.3 (2C), 126.0, 127.2 (2C), 127.9, 131.8, 133.1, 141.7, 143.4, 159.4, 187.4; IR (KBr)/cm^−1^, 2970, 1682, 1582, 1466, 1265, 1011; HRMS (ESI): calcd for C_16_H_13_NO_3_ (MH^+^) 268.0974; found 268.0980.

#### 1-(4-Methoxyphenyl)-2-(1-tosyl-1*H*-indol-3-yl)-1*H*-pyrrole-3-carbaldehyde (7a)

Yellow solid (107 mg, 76%, mp = 119–121 °C); ^1^H NMR (400 MHz, CDCl_3_) *δ* 2.37 (s, 3H), 3.78 (s, 3H), 6.71 (d, *J* = 8.9 Hz, 2H), 6.9 (d, *J* = 3.1 Hz, 1H), 6.97 (d, *J* = 2.4 Hz, 1H), 7.02 (d, *J* = 8.9 Hz, 2H), 7.12–7.16 (m, 1H), 7.23 (d, *J* = 8 Hz, 3H), 7.28–7.32 (m, 1H), 7.44 (s, 1H), 7.63 (d, *J* = 8.3 Hz, 2H), 7.95 (d, *J* = 8.3 Hz, 1H), 9.58 (s, 1H); ^13^C NMR (75 MHz, CDCl_3_) *δ* 29.6, 55.4, 108.1, 111.5, 113.5, 114.3 (2C), 120.3, 123.9, 125.2, 125.7, 125.8, 126.5 (2C), 126.8 (2C), 127.1, 129.9 (2C), 130.2, 131.7, 133.3, 134.4, 134.7, 145.3, 158.9, 186.1; IR (KBr)/cm^−1^, 2924, 2854, 1659, 1597, 1512, 1173; HRMS (ESI): calcd for C_27_H_22_N_2_O_4_S (MH^+^) 471.1378; found 471.1382.

#### 3-(3-Formyl-1-(4-methoxyphenyl)-1*H*-pyrrol-2-yl)-1-tosyl-1*H*-indole-5-carbonitrile (7b)

Brown solid (101 mg, 73%, mp = 123–125 °C); ^1^H NMR (400 MHz, CDCl_3_) *δ* 2.40 (s, 3H), 3.79 (s, 3H), 6.73 (d, *J* = 8.9 Hz, 2H), 6.90 (d, *J* = 3.1 Hz, 1H), 7.00–703 (m, 3H), 7.28 (d, *J* = 8.0 Hz, 2H), 7.45 (d, *J* = 8.8 Hz, 1H), 7.52 (dd, *J* = 7.1 Hz, 1H), 7.64 (s, 1H), 7.67 (d, *J* = 8.4 Hz, 2H), 8.05 (d, *J* = 8.1 Hz, 1H), 9.60 (s, 1H); ^13^C-NMR (75 MHz, CDCl_3_) *δ* 29.6, 55.5, 109.1, 111.4, 114.4, 114.5 (2C), 125.4, 126.0, 126.5 (2C), 126.9 (2C), 128.1, 128.1, 129.1, 129.2, 129.8, 130.2, 130.3 (2C), 130.9, 131.3, 134.1, 136.0, 146.2, 159.1, 185.5; IR (KBr)/cm^−1^, 2924, 2854, 2230, 1720, 1666, 1512, 1173; HRMS (ESI): calcd for C_28_H_21_N_3_O_4_S (MH^+^) 496.1331; found 496.1336.

#### 2-(5-Methoxy-1-tosyl-1*H*-indol-3-yl)-1-(4-methoxyphenyl)-1*H*-pyrrole-3-carbaldehyde (7c)

Red solid (105 mg, 70%, mp = 127–129 °C); ^1^H NMR (400 MHz, CDCl_3_) *δ* 2.37 (s, 3H), 3.64 (s, 3H), 3.78 (s, 3H), 6.57 (d, *J* = 2.4 Hz, 1H), 6.71 (d, *J* = 8.9 Hz, 2H), 6.88–6.9 (m, 2H), 6.98 (d, *J* = 2.7 Hz, 1H), 7.02 (d, *J* = 8.9 Hz, 2H), 7.22 (d, *J* = 8.2 Hz, 2H), 7.43 (s, 1H), 7.62 (d, *J* = 8.4 Hz, 2H), 7.82 (d, *J* = 9.1 Hz, 1H), 9.58 (s, 1H); ^13^C-NMR (75 MHz, CDCl_3_) *δ* 21.5, 55.4, 55.5, 102.0, 108.2, 111.5, 114.3 (2C), 114.5, 115.0, 125.6, 125.8, 126.3 (2C), 126.7 (2C), 127.9, 129.1, 129.9 (2C), 131.1, 131.8, 132.4, 134.7, 145.2, 156.8, 158.9, 186.1; IR (KBr)/cm^−1^, 2924, 2854, 1720, 1659, 1512, 1173; HRMS (ESI): calcd forC_28_H_24_N_2_O_5_S (MH^+^) 501.1484; found 501.1488.

#### 2-(5-Bromo-1-tosyl-1*H*-indol-3-yl)-1-(4-methoxyphenyl)-1*H*-pyrrole-3-carbaldehyde (7d)

Yellow solid (115 mg, 70%, mp = 139–141 °C); ^1^H NMR (400 MHz, CDCl_3_) *δ* 2.38 (s, 3H), 3.79 (s, 3H), 6.72 (d, *J* = 8.9 Hz, 2H), 6.89 (d, *J* = 3.2 Hz, 1H), 6.97 (d, *J* = 2.5 Hz, 1H), 7.00 (d, *J* = 8.9 Hz, 2H), 7.24 (d, *J* = 8.0 Hz, 2H), 7.30 (d, *J* = 1.7 Hz, 2H), 7.37 (d, *J* = 7.0 Hz, 1H), 7.45 (s, 1H), 7.60 (d, *J* = 8.4 Hz, 2H), 7.81 (d, *J* = 8.8 Hz, 1H), 9.57 (s, 1H); ^13^C NMR (400 MHz, CDCl_3_) *δ* 29.6, 55.5, 108.5, 110.9, 114.4 (2C), 115.0, 117.5, 123.1, 125.8, 125.9, 126.6 (2C), 126.8 (2C), 128.3, 128.3, 130.1 (2C), 131.5, 131.8, 132.3, 133.1, 134.4, 145.7, 159.1, 185.8; IR (KBr)/cm^−1^, 2924, 2854, 1720, 1666, 1512, 1250, 1119; HRMS (ESI): calcd for C_27_H_21_BrN_2_O_4_S (MH^+^) 549.0483; found 549.0488.

#### 2-(5-Chloro-1-tosyl-1*H*-indol-3-yl)-1-(4-methoxyphenyl)-1*H*-pyrrole-3-carbaldehyde (7e)

Brown solid (102 mg, 68%, mp = 134–136 °C); ^1^H NMR (400 MHz, CDCl_3_) *δ* 2.17 (s, 3H), 3.58 (s, 3H), 6.52 (d, *J* = 8.9 Hz, 2H), 6.67 (d, *J* = 3.1 Hz, 1H), 6.76 (dd, *J* = 2.4 Hz, 1H), 6.80 (d, *J* = 8.9 Hz, 2H), 6.95 (d, *J* = 1.9 Hz, 1H), 7.02–7.05 (m, 4H), 7.40 (d, *J* = 8.4 Hz, 2H), 7.65 (d, *J* = 8.8 Hz, 1H), 9.36 (s, 1H); ^13^C NMR (75 MHz, CDCl_3_) *δ* 29.7, 55.5, 108.4, 111.0, 114.4 (2C), 114.6, 120.0, 125.6, 125.8, 125.9, 126.6 (2C), 126.8 (2C), 128.5, 130.0, 130.1 (2C), 131.4, 131.5, 132.5, 132.8, 134.5, 145.6, 159.1, 185.8; IR (KBr)/cm^−1^, 2924, 2854, 1666, 1512, 1250, 1173; HRMS (ESI): calcd for C_27_H_21_ClN_2_O_4_S (MH^+^) 505.0911; found 505.0916.

#### 1-(4-Methoxyphenyl)-2-(1-(phenylsulfonyl)-1*H*-indol-3-yl)-1*H*-pyrrole-3-carbaldehyde (7f)

Brown solid (96 mg, 70%, mp = 117–119 °C); ^1^H NMR (400 MHz, CDCl_3_), *δ* 3.77 (s, 3H), 6.69 (d, *J* = 8.93 Hz, 2H), 6.89 (d, *J* = 3.1 Hz, 1H), 6.96 (d, *J* = 3.3 Hz, 1H), 7.00 (d, *J* = 8.9 Hz, 2H), 7.12–7.16 (m, 1H), 7.23 (d, *J* = 7.6 Hz, 1H), 7.28–7.32 (m, 1H), 7.42–7.46 (m, 3H), 7.54–7.59 (m, 1H), 7.72–7.74 (m, 2H), 7.96 (d, *J* = 8.3 Hz, 1H), 9.58 (s, 1H); ^13^C NMR (75 MHz, CDCl_3_), *δ* 55.4, 108.1, 111.7, 113.4, 114.3 (2C), 120.3, 123.9, 125.3, 125.7, 126.5 (2C), 126.6 (2C), 127.1, 129.3 (2C), 130.2, 131.6, 132.2, 134.0, 134.4 (2C), 137.6, 158.8, 186.1; IR (KBr)/cm^−1^, 2932, 2839, 1720, 1666, 1512, 1225, 1180; HRMS (ESI): calcd for C_26_H_20_N_2_O_4_S (MH^+^) 457.1222; found 457.1226.

#### 2-(5-Methoxy-1-(phenylsulfonyl)-1*H*-indol-3-yl)-1-(4-methoxyphenyl)-1*H*-pyrrole-3-carbaldehyde (7g)

Yellow viscous liquid (99 mg, 68%); ^1^H NMR (400 MHz, CDCl_3_) *δ* 3.64 (s, 3H), 3.77 (s, 3H), 6.56 (d, *J* = 2.4 Hz, 1H), 6.70 (d, *J* = 8.9 Hz, 2H), 6.88–6.91 (m, 2H), 6.98 (d, *J* = 2.4 Hz, 1H), 7.00 (d, *J* = 8.9 Hz, 2H), 7.42–7.45 (m, 3H), 7.54–7.59 (m, 1H), 7.71–7.73 (m, 2H), 7.84 (d, *J* = 9.1 Hz, 1H), 9.58 (s, 1H); ^13^C NMR (75 MHz, CDCl_3_) *δ* 55.4, 55.5, 102.0, 108.2, 111.8, 114.3 (2C), 114.5, 115.0, 125.6, 125.8, 126.3 (2C), 126.6 (2C), 127.8, 129.1, 129.3 (2C), 131.1, 131.7, 133.3, 134.0, 137.6, 156.9, 158.9, 186.1; IR (KBr)/cm^−1^, 2924, 2854, 1728, 1666, 1512, 1466, 1250, 1180; HRMS (ESI): calcd for C_27_H_22_N_2_O_5_S (MH^+^) 487.1327; found 487.1332.

#### 2-(5-Bromo-1-(phenylsulfonyl)-1*H*-indol-3-yl)-1-(4-methoxyphenyl)-1*H*-pyrrole-3-carbaldehyde (7h)

Brownish yellow gummy liquid (107 mg, 67%); ^1^H NMR (400 MHz, CDCl_3_) *δ* 3.82 (s, 3H), 3.76 (d, *J* = 8.9 Hz, 2H), 6.91 (d, *J* = 3.1 Hz, 1H), 7.00 (d, *J* = 3.1 Hz, 1H), 7.3 (d, *J* = 8.9 Hz, 2H), 7.35 (d, *J* = 1.5 Hz, 1H), 7.42 (d, *J* = 6.9 Hz, 1H), 7.48–7.51 (m, 3H), 7.60–7.65 (m, 1H), 7.74 (d, *J* = 7.3 Hz, 2H), 7.86 (d, *J* = 8.8 Hz, 1H), 9.60 (s, 1H); ^13^C NMR (75 MHz, CDCl_3_) *δ* 55.5, 108.4, 111.1, 114.4 (2C), 114.9, 117.6, 123.1, 123.9, 125.8, 126.6 (2C), 126.7 (2C) 128.2, 128.4, 129.5 (2C), 131.4, 131.8, 132.1, 133.1, 134.3, 137.3, 159.0, 185.8; IR (KBr)/cm^−1^, 2924, 2854, 1666, 1572, 1443, 1250, 1180; HRMS (ESI): calcd for C_26_H_19_BrN_2_O_4_S (MH^+^) 535.0319; found 535.0325.

#### 1-(4-Methoxyphenyl)-2-(1-(methylsulfonyl)-1*H*-indol-3-yl)-1*H*-pyrrole-3-carbaldehyde (7i)

Yellow pasty liquid (82 mg, 70%); ^1^H NMR (400 MHz, CDCl_3_) *δ* 3.07 (s, 3H), 3.75 (s, 3H), 6.77 (d, *J* = 9.0 Hz, 2H), 6.91 (d, *J* = 3.1 Hz, 1H), 7.01 (d, *J* = 2.4 Hz, 1H), 7.08 (d, *J* = 9.0 Hz, 2H), 7.21–7.25 (m, 1H), 7.31–7.34 (m, 2H), 7.35–7.39 (m, 1H), 7.90 (d, *J* = 8.4 Hz, 1H), 9.70 (s, 1H); ^13^C NMR (75 MHz, CDCl_3_) *δ* 40.9, 55.4, 108.4, 113.0, 114.3 (2C), 120.7, 124.2, 125.3, 125.6, 125.7, 125.9, 126.5 (2C), 126.9, 130.2, 131.7, 132.9, 134.6, 159.1, 186.1; IR (KBr)/cm^−1^ 2924, 2854, 1659, 1443, 1373, 1134; HRMS (ESI): calcd for C_21_H_18_N_2_O_4_S (MH^+^) 395.1065; found 395.1070.

#### 3-(3-Formyl-1-(4-methoxyphenyl)-1*H*-pyrrol-2-yl)-1-(methylsulfonyl)-1*H*-indole-5-carbonitrile (7j)

Brownish solid (85 mg, 68%, mp = 114–116 °C); ^1^H NMR (400 MHz, CDCl_3_) *δ* 3.17 (s, 3H), 3.77 (s, 3H), 6.8 (d, *J* = 8.9 Hz, 2H), 6.93 (d, *J* = 3.1 Hz, 1H), 7.03 (d, *J* = 2.6 Hz, 1H), 7.09 (d, *J* = 9.0 Hz, 2H), 7.53 (m, 1H), 7.58 (d, *J* = 7.1 Hz, 1H), 7.99–8.03 (m, 2H), 9.73 (s, 1H); ^13^C NMR (75 MHz, CDCl_3_) *δ* 41.8, 55.5, 107.9, 109.6, 111.5, 114.1, 114.6 (2C), 118.6, 121.5, 125.8, 126.0, 126.5 (2C), 128.0, 128.4, 129.0, 131.3, 137.1, 141.7, 159.4, 185.6; IR (KBr)/cm^−1^, 2924, 2854, 2230, 1659, 1512, 1381, 1180; HRMS (ESI): calcd for C_22_H_17_N_3_O_4_S (MH^+^) 419.0940; found 419.0946.

#### 2-(5-Chloro-1-(methylsulfonyl)-1*H*-indol-3-yl)-1-(4-methoxyphenyl)-1*H*-pyrrole-3-carbaldehyde (7k)

Yellowish solid (85 mg, 66%, mp = 126–128 °C); ^1^H NMR (400 MHz, CDCl_3_) *δ* 3.07 (s, 3H), 3.77 (s, 3H), 6.79 (d, *J* = 8.9 Hz, 2H), 6.91 (d, *J* = 3.1 Hz, 1H), 7.01 (d, *J* = 2.8 Hz, 1H), 7.08 (d, *J* = 8.9 Hz, 2H), 7.25 (d, *J* = 1.9 Hz, 1H), 7.31 (d, *J* = 6.8 Hz, 1H), 7.36 (s, 1H), 7.82 (d, *J* = 8.8 Hz, 1H), 9.70 (s, 1H); ^13^C NMR (75 MHz, CDCl_3_) *δ* 40.1, 54.5, 107.8, 113.2, 113.4 (2C), 119.3, 121.6, 124.8, 124.9, 125.6 (2C), 126.0, 127.2, 129.2, 130.2, 130.4, 131.9, 135.6, 158.2, 184.8; IR (KBr)/cm^−1^, 2924, 2854, 1666, 1572, 1443, 1250, 1180; HRMS (ESI): calcd for C_21_H_17_ClN_2_O_4_S (MH^+^) 429.0676; found 429.0682.

#### *tert*-Butyl-3-(3-formyl-1-(4-methoxyphenyl)-1*H*-pyrrol-2-yl)-1*H*-indole-1-carboxylate (7l)

White solid, (92 mg, 74%, mp = 117–119 °C); ^1^H NMR (400 MHz, CdCl_3_), *δ* 1.67 (s, 9H), 3.74 (s, 3H), 6.66 (d, *J* = 9.0 Hz, 2H), 6.92 (d, *J* = 3.2 Hz, 1H), 6.98 (d, *J* = 2.5 Hz, 1H), 7.07–7.16 (m, 5H), 7.28–7.30 (m, 1H), 7.57 (s, 1H), 8.12 (d, *J* = 8.1 Hz, 1H), 9.36 (s, 1H); ^13^C NMR (75 MHz, CDCl_3_) *δ* 28.1 (3C), 29.7, 55.44, 84.5, 108.0, 109.7, 114.3 (2C), 115.1, 120.1, 123.2, 124.9, 125.5, 125.9, 126.3 (2C), 127.2, 132.0, 134.7, 139.3, 149.2, 158.8, 186.7; IR (KBr)/cm^−1^, 2934, 2860, 1726, 1666, 1512, 1250, 1157; HRMS (ESI): calcd for C_25_H_24_N_2_O_4_ (MH^+^) 417.1814; found 417.1820.

#### *tert*-Butyl-5-cyano-3-(3-formyl-1-(4-methoxyphenyl)-1*H*-pyrrol-2-yl)-1*H*-indole-1-carboxylate (7m)

Reddish brown solid (95 mg, 72%, mp = 120–122 °C); ^1^H NMR (400 MHz, CDCl_3_) *δ* 1.05 (s, 9H), 3.59 (s, 3H), 6.53 (d, *J* = 8.9 Hz, 2H), 6.70 (d, *J* = 3.1 Hz, 1H), 6.82 (d, *J* = 9.0 Hz, 3H), 7.07 (d, *J* = 3.7 Hz, 1H), 7.09 (s, 1H), 7.32 (d, *J* = 1.4 Hz, 1H), 7.84 (d, *J* = 8.7 Hz, 1H), 9.40 (s, 1H); ^13^C NMR (75 MHz, CDCl_3_) *δ* 29.7 (3C), 31.3, 55.5, 107.5, 109.1, 111.3, 114.5 (2C), 118.7, 125.5, 126.0, 126.5 (2C), 126.9, 128.1, 129.2, 129.9, 130.3, 131.0, 131.3, 134.1, 136.0, 146.2, 159.1, 185.6; IR (KBr)/cm^−1^, 2932, 2862, 2230, 1666, 1512, 1173, 1250; HRMS (ESI): calcd for C_26_H_23_N_3_O_4_ (MH^+^) 442.1767; found 442.1774.

#### *tert*-Butyl-5-fluoro-3-(3-formyl-1-(4-methoxyphenyl)-1*H*-pyrrol-2-yl)-1*H*-indole-1-carboxylate (7n)

Yellow solid (87 mg, 67%, mp = 119–121 °C); ^1^H NMR (400 MHz, CDCl_3_) *δ* 1.66 (s, 9H), 3.75 (s, 3H), 6.74–6.79 (m, 3H), 6.90 (d, *J* = 3.1 Hz, 1H), 6.97–7.00 (m, 2H), 7.13 (d, *J* = 8.9 Hz, 2H), 7.62 (s, 1H), 8.07 (d, *J* = 5.9 Hz, 1H), 9.68 (s, 1H), ^13^C-NMR (75 MHz, CDCl_3_), *δ* 28.1 (3C), 29.7, 55.4, 84.8, 105.6, 105.8, 108.2, 109.6, 112.8, 113.0, 114.4 (2C), 116.2, 125.6, 126.3 (2C), 128.6, 131.9, 134.0, 149.0, 158.9, 160.3, 186.5; IR (KBr)/cm^−1^, 2924, 2854, 1736, 1666, 1450, 1366, 1250, 1172; HRMS (ESI): calcd for C_25_H_23_FN_2_O_4_ (MH^+^) 435.1720; found 435.1726.

#### *tert*-Butyl-5-bromo-3-(3-formyl-1-(4-methoxyphenyl)-1*H*-pyrrol-2-yl)-1*H*-indole-1-carboxylate (7o)

White solid (82 mg, 68%, mp = 129–131 °C); ^1^H NMR (400 MHz, CDCl_3_) *δ* 1.66 (s, 9H), 3.02 (s, 3H), 6.79 (d, *J* = 8.98 Hz, 2H), 6.90 (d, *J* = 3.12 Hz, 1H), 6.98 (d, *J* = 3.9 Hz, 1H), 7.12 (d, *J* = 8.9 Hz, 2H), 7.22 (d, *J* = 1.7 Hz, 1H), 7.36 (d, *J* = 6.9 Hz, 1H), 7.75 (s, 1H), 7.99 (d, *J* = 8.4 Hz, 1H), 9.68 (s, 1H), ^13^C NMR (75 MHz, CDCl_3_), *δ* 28.1 (3C), 29.7, 55.5, 85.8, 108.2, 109.1, 114.4 (2C), 116.6, 122.8, 125.6, 125.9, 126.4 (2C) 127.8, 128.2, 131.1, 131.8, 133.7, 133.8, 148.8, 159.0, 186.3; IR (KBr)/cm^−1^, 2932, 2862, 1736, 1680, 1458, 1373, 1157; HRMS (ESI): calcd for C_25_H_23_BrN_2_O_4_ (MH^+^) 495.0919; found 405.0924.

#### *tert*-Butyl-3-(3-formyl-1-(4-methoxyphenyl)-1*H*-pyrrol-2-yl)-5-methoxy-1*H*-indole-1-carboxylate (7p)

Dark brown solid (89 mg, 70%, mp = 124–126 °C); (400 MHz, CDCl_3_), *δ* 1.66 (s, 9H), 3.64 (s, 3H), 3.75 (s, 3H), 6.51 (d, *J* = 2.4 Hz, 1H), 6.78 (d, *J* = 8.9 Hz, 2H), 6.87 (d, *J* = 6.6 Hz, 1H), 6.93 (d, *J* = 3.2 Hz, 1H), 7.00 (d, *J* = 2.4 Hz, 1H), 7.15 (d, *J* = 9 Hz, 2H), 7.57 (s, 1H), 7.99 (d, *J* = 8.7 Hz, 1H), 9.71 (s, 1H); ^13^C-NMR (100 MHz, CDCl_3_) *δ* 28.1 (3C), 29.6, 55.4, 55.5, 84.3, 102.0, 108.0, 109.5, 114.3 (2C), 115.9, 125.4, 125.8, 126.1 (2C) 127.7, 129.6, 130.2, 132.2, 134.8, 149.1, 156.1, 158.8, 186.6; IR (KBr)/cm^−1^, 2932, 2862, 1736, 1666, 1512, 1250, 1157; HRMS (ESI): calcd for C_26_H_26_N_3_O_5_ (MH^+^) 425.1171; found 425.1176.

#### 1-(2-Hydroxyphenyl)-2-(1-tosyl-1*H*-indol-3-yl)-1*H*-pyrrole-3-carbaldehyde (7q)

Light yellow solid (82 mg, 60%, mp = 118–120 °C); ^1^H NMR (400 MHz, CDCl_3_) *δ* 2.42 (s, 3H), 6.95 (d, *J* = 3.2 Hz, 1H), 7.07–7.01 (m, 3H), 7.22–7.15 (m, 3H), 7.25 (d, *J* = 7.4 Hz, 1H), 7.30 (d, *J* = 8.5 Hz, 2H), 7.36–7.38 (m, 1H), 7.46 (s, 1H), 7.67 (d, *J* = 8.4 Hz, 2H), 8.02 (d, *J* = 8.4 Hz, 1H), 9.63 (s, 1H); ^13^C NMR (75 MHz, CDCl_3_) *δ* 21.6, 108.7, 111.0, 113.6, 120.1, 124.02, 125.2, 125.4, 126.3, 126.4 (2C), 126.7 (2C), 126.9, 127.0, 129.3 (2C), 130.0 (2C), 132.8, 133.5, 134.4, 134.6, 137.1, 145.5, 186.0; IR (KBr)/cm^−1^, 3458, 2924, 2854, 1659, 1497, 1443, 1088; HRMS (ESI): calcd for C_26_H_20_N_2_O_4_S (MH^+^) 457.1222; found 457.1227.

#### 1-(4-Chlorophenyl)-2-(1-tosyl-1*H*-indol-3-yl)-1*H*-pyrrole-3-carbaldehyde (7r)

Light pinkish solid (91 mg, 64%, mp = 132–134 °C) ^1^H NMR (400 MHz, CDCl_3_) *δ* 2.35 (s, 3H), 6.70 (m, 1H), 6.78 (s, 1H), 6.87 (m, 2H), 6.97 (d, *J* = 7.9 Hz, 1H), 7.25–7.10 (m, 4H), 7.37–7.28 (m, 2H), 7.40 (d, *J* = 7.9 Hz, 1H), 7.56–7.50 (m, 2H), 7.90 (d, *J* = 8.4 Hz, 1H), 9.43 (s, 1H), ^13^C NMR (75 MHz, CDCl_3_) *δ* 21.6, 108.4, 110.7, 113.4, 117.3, 120.0, 120.4, 123.9, 125.2, 125.2, 125.6, 126.1, 126.7 (2C), 127.0, 128.2, 129.9 (2C), 130.1, 130.3, 134.2, 134.6, 135.1, 145.1, 151.8, 186.0; IR (KBr)/cm^−1^, 2924, 2854, 1651, 1504, 1443, 1173; HRMS (ESI): calcd for C_26_H_19_ClN_2_O_3_S (MH^+^) 475.0883; found 475.0888.

#### 1-(4-Methoxyphenyl)-1*H*-pyrrolo[3,2-*c*] quinoline (8)

To a stirred solution of 5a (50 mg, 0.15 mmol) in EtOH : H_2_O (5 mL, 4 : 1) was added Fe-powder (86.9 mg, 1.55 mmol, 10.0 equiv.) and NH_4_Cl (100 mg, 1.8 mmol, 12.0 equiv.) and combined mixture was heated at 80 °C for 10 h. The reaction progress was monitored by TLC, cooled and concentrated under reduced pressure once completed. The crude residue was extracted between EtOAc/NaHCO_3_ solutions. Organic layer dried over Na_2_SO_4_ and evaporated under reduced pressure followed by silica-gel chromatography purification by eluting the mixture of hexane/EtOAc, gave pure product 8 as pasty yellow liquid (28 mg, 67% yield). ^1^H NMR (400 MHz, CDCl_3_) *δ* 3.95 (s, 3H), 6.87 (d, *J* = 3.1 Hz, 1H), 7.09 (d, *J* = 8.6 Hz, 2H), 7.21 (d, *J* = 3.1 Hz, 1H), 7.23 (m, 1H), 7.38 (d, *J* = 9.1 Hz, 1H), 7.43 (d, *J* = 8.8 Hz, 2H), 7.53 (t, *J* = 7.0 Hz, 1H), 8.19 (d, *J* = 8.3 Hz, 1H), 9.21 (s, 1H); ^13^C NMR (75 MHz, CDCl_3_) *δ* 55.6, 103.3, 114.8 (2C), 118.2, 120.6, 121.6, 125.3, 126.4, 128.5 (2C), 129.9, 130.2, 133.2, 134.6, 144.2, 146.1, 159.9; IR (KBr)/cm^−1^, 2924, 1713, 1512, 1366, 1250, 1034; HRMS (ESI): calcd for C_18_H_14_N_2_O (MH^+^) 275.1185; found 275.1190.

#### 1,5-Bis(4-methoxyphenyl)-4,5-dihydro-1*H*-pyrrolo[3,2-*c*] quinoline (10)

A mixture of 5e (0.1 g, 0.28 mmol, 1.0 equiv.) and *p*-anisidine 3 (0.030 g, 0.28 mmol, 1.0 equiv.) in methanol (3 mL) was refluxed for 2 h at 80 °C and followed by reductive amination in the presence of NaBH_4_ at 0 °C to obtain intermediate product. This crude intermediate 9 (0.1 g, 0.22 mmol, 1.0 equiv.) was taken in oven-dried round-bottom flask dissolved in DMF (2 mL), followed by the addition of base K_2_CO_3_ (61 mg, 0.44 mmol, 2.0 equiv.), CuI (9 mg, 20 mol%), l-proline as ligand (10 mg, 40 mol%). The resulting solution was stirred at 110 °C for 3 h under an N_2_ atmosphere. On completion, the residue was cooled to ambient temperature and then diluted with water (5 mL) and extracted with EtOAc (2 × 5 mL). The combined organic layers were dried over anhydrous Na_2_SO_4_ and evaporated to dryness. The crude residue was purified by column chromatography by eluting the mixture of hexane/EtOAc, afford 10 as yellow pasty liquid (70 mg, 87% yield). ^1^H NMR (400 MHz, CDCl_3_) *δ* 7.67 (dd, *J* = 7.8, 1.7 Hz, 1H), 7.56 (dd, *J* = 7.9, 1.2 Hz, 1H), 7.28–7.33 (m, 1H), 7.23 (d, *J* = 9.0 Hz, 2H), 7.08–7.14 (m, 1H), 6.93 (d, *J* = 2.4 Hz, 1H), 6.90 (d, *J* = 9.0 Hz, 2H), 6.74 (d, *J* = 1.8 Hz, 1H), 6.72 (d, *J* = 9.0 Hz, 2H), 6.47 (d, *J* = 8.9 Hz, 2H), 6.25 (d, *J* = 1.8 Hz, 1H), 5.80 (s, 1H), 3.81 (s, 3H), 3.70 (s, 3H); ^13^C NMR (75 MHz, CDCl_3_) *δ* 55.5, 55.7, 56.2, 109.2, 114.2 (2C), 114.5 (2C), 114.7 (2C), 118.1, 119.9, 121.9 (2C), 123.5, 126.7, 127.8, 128.1, 128.4, 132.9, 134.1, 141.3, 142.2, 151.9, 157.6; HRMS (ESI): calcd for C_25_H_22_N_2_O_2_ (MH^+^) 383.1759; found 383.1765.

#### 2-(1-(4-Methoxyphenyl)-2-(4-nitrophenyl)-1*H*-pyrrol-3-yl)-5-(4-nitrophenyl)-1,3,4-oxadiazole (11)

A mixture of 5c (0.08 g, 0.24 mmol, 1.0 equiv.) and 4-nitrobenzohydrazide (0.044 g, 0.24 mmol, 1.0 equiv.) in methanol (3.0 mL) was stirred for 2 hours under 80 °C and then concentrated *in vacuo*. The crude material was taken in CH_3_CN (3.0 mL) and IBD (0.08 g, 0.24 mmol, 1.0 equiv.) was added stirred at room temperature for one hour. The mixture was then concentrated *in vacuo*. To the residue was added H_2_O (10 mL) and the resulting mixture was extracted with ethylacetate (10 mL × 3). The organic layer was dried over Na_2_SO_4_ and concentrated. The residue was purified by column chromatography on silica gel by eluting the mixture of hexane/EtOAc, afford product 11 as yellow solid (92 mg, 76% yield). ^1^H NMR (400 MHz, CDCl_3_) (mp = 112–115 °C) *δ* 8.33 (d, *J* = 9.0 Hz, 2H), 8.17 (d, *J* = 8.9 Hz, 2H), 8.08 (d, *J* = 9.0 Hz, 2H), 7.52 (d, *J* = 8.9 Hz, 2H), 7.07 (d, *J* = 3.0 Hz, 1H), 7.04 (d, *J* = 9.0 Hz, 2H), 7.01 (d, *J* = 3.0 Hz, 1H), 6.84 (d, *J* = 9.0 Hz, 2H), 3.81 (s, 3H); ^13^C NMR (75 MHz, CDCl_3_) *δ* 55.5, 108.0, 109.8, 114.5 (2C), 123.0 (2C), 124.3 (2C), 125.9, 127.1 (2C), 127.3 (2C), 129.4, 131.3, 132.0 (2C), 132.4, 137.0, 147.3, 149.2, 159.2, 161.6, 162.7; HRMS (ESI): calcd for C_25_H_17_N_5_O_6_ (MH^+^) 484.1258; found 484.1263.

#### 7-Methoxypyrrolo [1,2-*f*] phenanthridine-1-carbaldehyde (13)

A clean oven-dried 10 mL round-bottom flask was charged with 5e (70 mg, 0.19 mmol, 1.0 equiv.) in DMF (2 mL), K_2_CO_3_ (54 mg, 0.39 mmol, 2.0 equiv.), ligand PPh_3_ (10 mg, 20 mol%), and Pd(OAc)_2_ (5 mg, 10 mol%). The resulting solution was stirred at 130 °C for 3 h under an N_2_ atmosphere. On completion, the reaction mass was cooled to ambient temperature and then diluted with water (5 mL) and extracted with EtOAc (2 × 5 mL). The combined organic layers were dried over anhydrous Na_2_SO_4_ and evaporated to dryness. The crude residue so obtained was purified by column chromatography by eluting the mixture of hexane/EtOAc, afford 13 as white solid (43 mg, 78% yield). ^1^H NMR (400 MHz, CDCl_3_) *δ* 10.26 (s, 1H), 9.49 (dd, *J* = 7.1, 2.3 Hz, 1H), 8.33 (dd, *J* = 7.3, 2.2 Hz, 1H), 7.88 (d, *J* = 9.2 Hz, 1H), 7.85 (d, *J* = 2.7 Hz, 1H), 7.80 (d, *J* = 3.3 Hz, 1H), 7.62–7.70 (m, 2H), 7.24 (d, *J* = 3.3 Hz, 1H), 7.22 (dd, *J* = 9.1, 2.8 Hz, 1H), 3.99 (s, 3H); ^13^C NMR (75 MHz, CDCl_3_) *δ* 55.7, 106.6, 114.0, 115.1, 117.0, 117.2, 117.7, 119.0, 120.5, 122.2, 123.9, 125.3, 127.2, 127.4, 128.5, 128.7, 157.2, 185.1; HRMS (ESI): calcd for C_18_H_13_BrNO_2_ (MH^+^) 276.1024; found 276.1029.

## Conflicts of interest

There are no conflicts to declare.

## Supplementary Material

RA-008-C8RA01637B-s001

RA-008-C8RA01637B-s002
