# Supplementary material for: One-pot sequential multicomponent reaction between in situ generated aldimines and succinaldehyde: facile synthesis of substituted pyrrole-3-carbaldehydes and applications towards medicinally important fused heterocycles
Source: RSC Adv. 2018 Apr 24;8(28):15448–58. doi: 10.1039/c8ra01637b (PMC9080005; doi:10.1039/c8ra01637b)
Supplement: RA-008-C8RA01637B-s001 [file RA-008-C8RA01637B-s001.pdf]

Electronic Supplementary Material (ESI) for RSC Advances.
This journal is © The Royal Society of Chemistry 2018

Supporting Information

One-pot sequential multicomponent reaction between in situ generated aldimines and succinaldehyde: Facile synthesis of substituted pyrrole-3-carbaldehydes and applications towards medicinally important fused heterocycles

Anoop Singh,[a] Nisar A. Mir, [a] Sachin Choudhary,[a] Deepika Singh,[b] Preetika Sharma,[c] Rajni Kant[c] and Indresh Kumar*[a]

Table of Contents:

Copies of ^{1}H and ^{13}C-NMR Spectra for all compounds..S2- S38
Single Crystal X-ray and data for **5c** and **7e**………………………………….........S39-S45

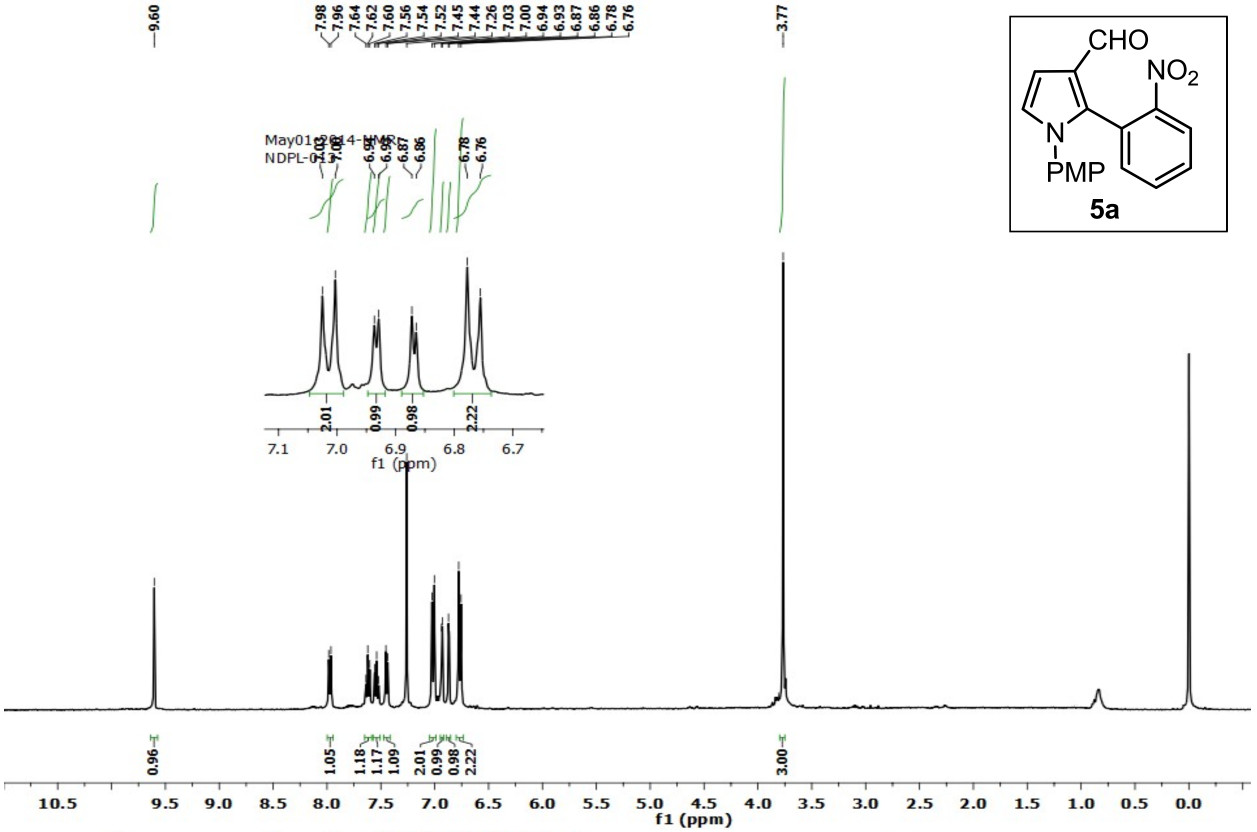
9.60
7.98
7.96
7.64
7.62
7.60
7.56
7.54
7.52
7.45
7.44
7.26
7.03
7.00
6.94
6.93
6.87
6.86
6.78
6.76
3.77
CHO
NO_2
N
PMP
5a
f1 (ppm)

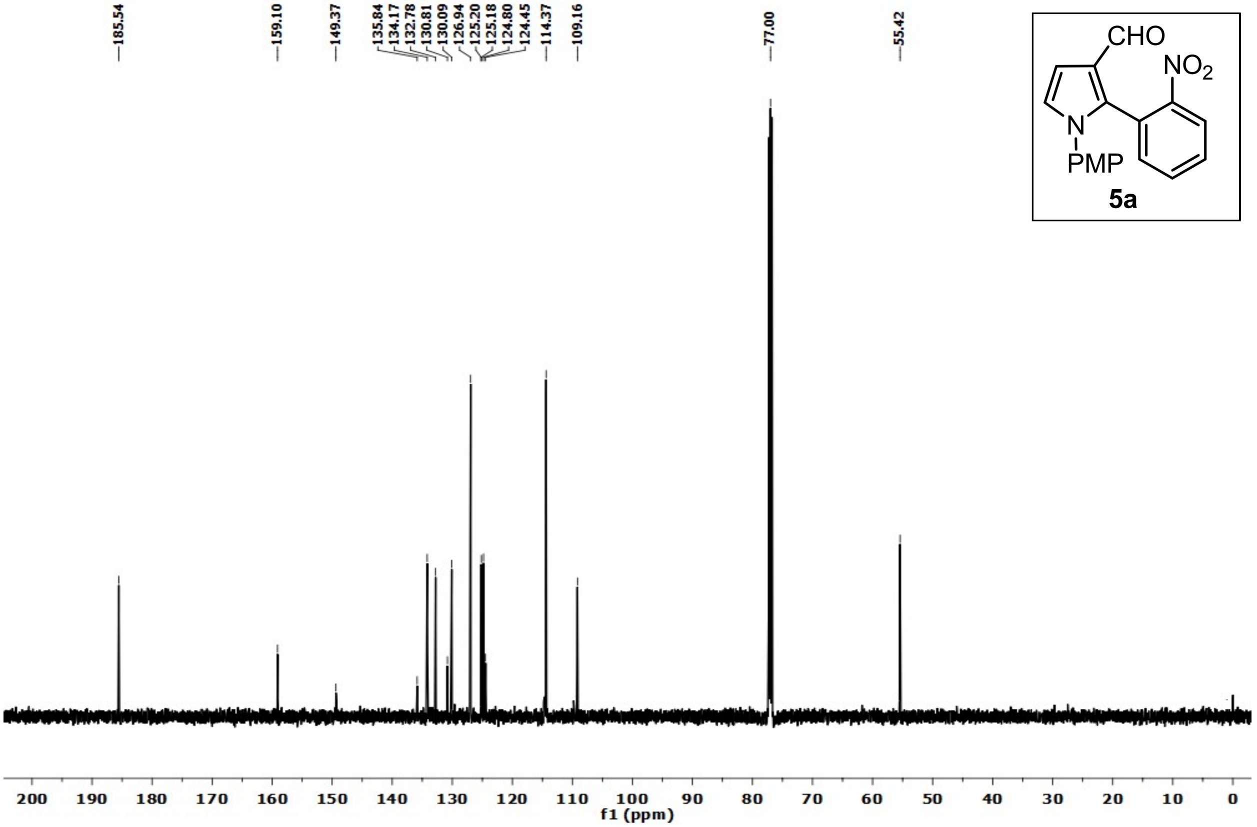
185.54
159.10
149.37
135.84
134.17
132.78
130.81
130.09
126.94
125.20
125.18
124.80
124.45
114.37
109.16
77.00
55.42
CHO
NO_2
N
PMP
5a
f1 (ppm)

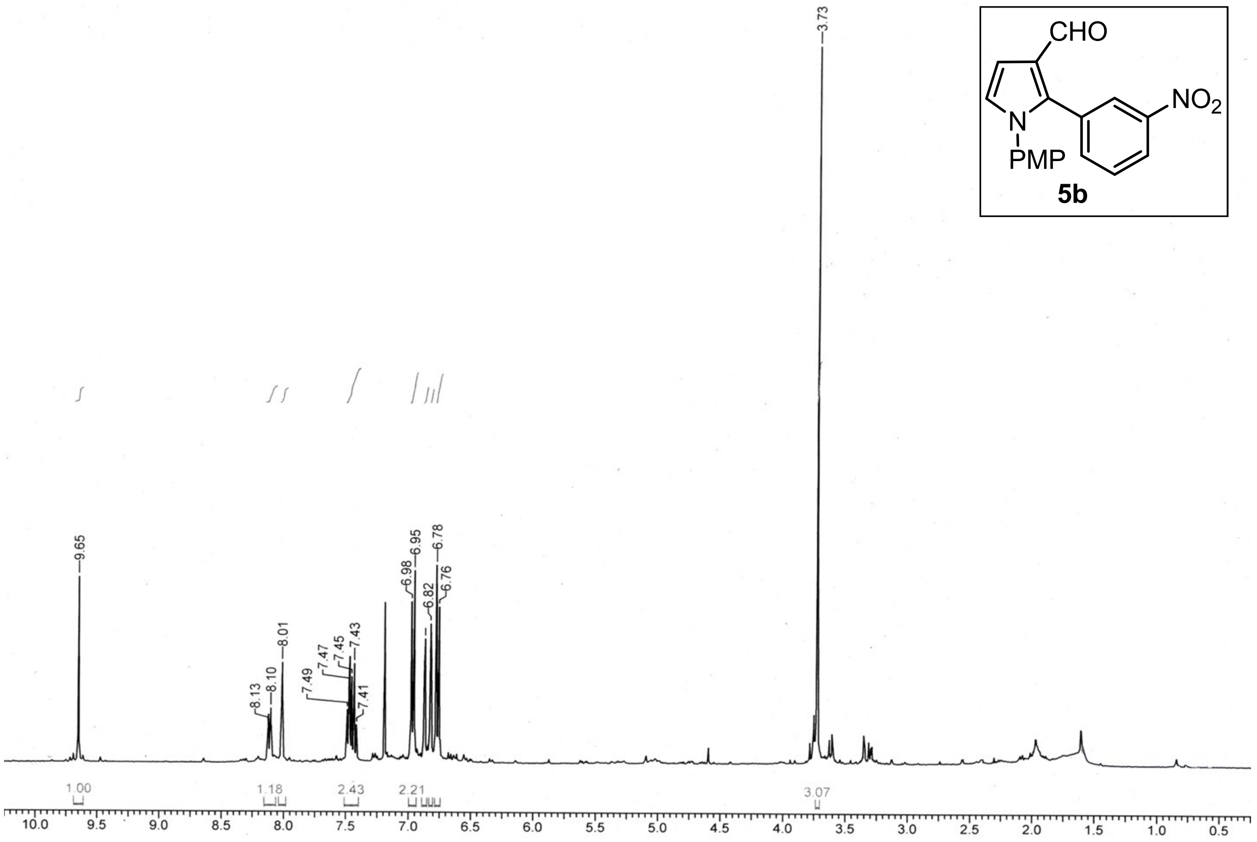
CHO
NO2
N
PMP
5b
3.73
9.65
8.13
8.10
8.01
7.49
7.47
7.45
7.43
7.41
6.98
6.95
6.82
6.78
6.76
1.00
1.18
2.43
2.21
3.07
10.0 9.5 9.0 8.5 8.0 7.5 7.0 6.5 6.0 5.5 5.0 4.5 4.0 3.5 3.0 2.5 2.0 1.5 1.0 0.5

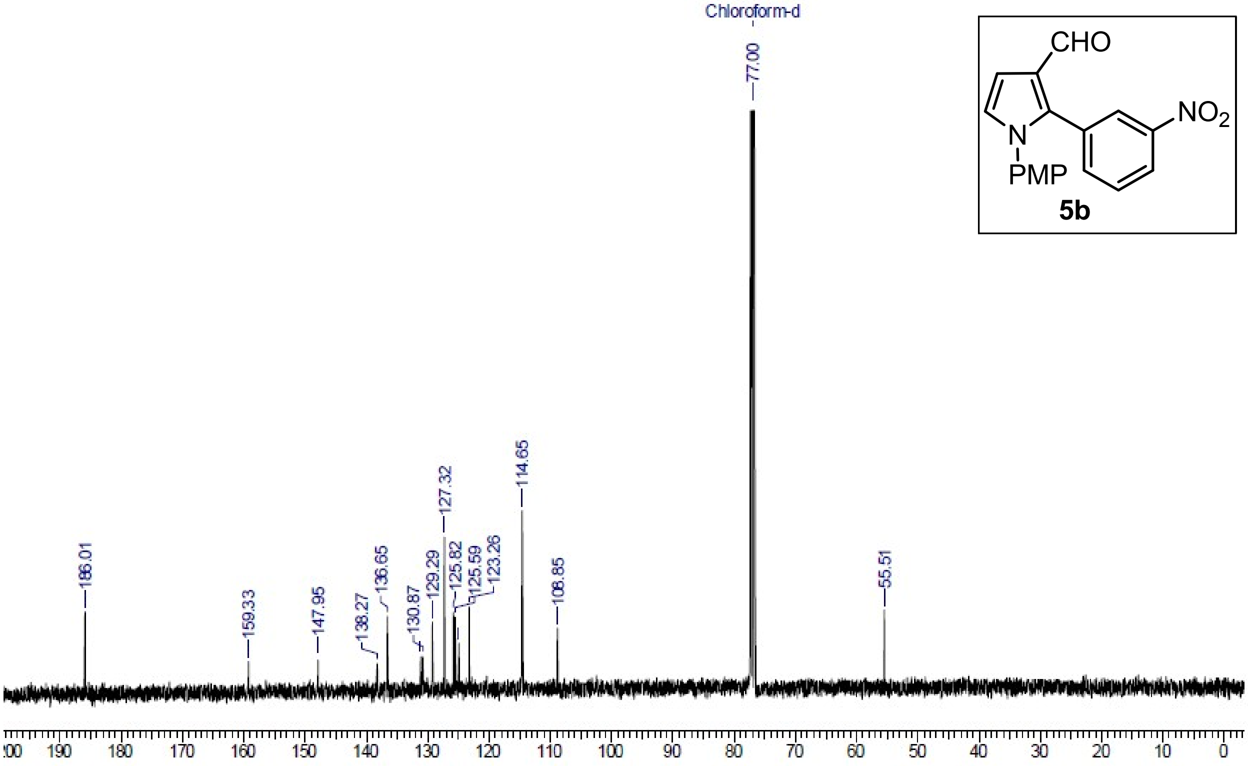
Chloroform-d
77.00
CHO
NO2
N
PMP
5b
186.01
159.33
147.95
138.27
136.65
130.87
129.29
127.32
125.82
125.59
123.26
114.65
108.85
55.51
200 190 180 170 160 150 140 130 120 110 100 90 80 70 60 50 40 30 20 10 0

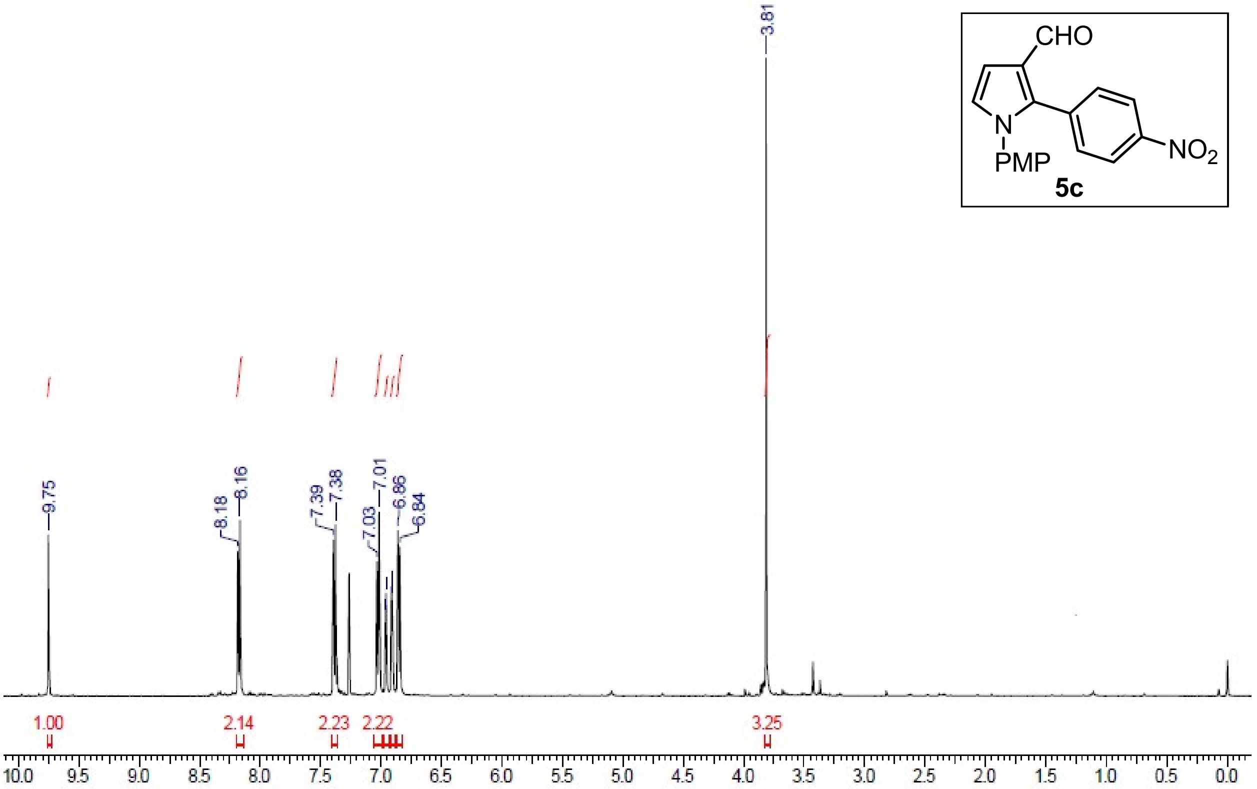

CHO
N
PMP
NO_2
5c
9.75
8.18
8.16
7.39
7.38
7.03
7.01
6.86
6.84
3.81
1.00
2.14
2.23
2.22
3.25
10.0 9.5 9.0 8.5 8.0 7.5 7.0 6.5 6.0 5.5 5.0 4.5 4.0 3.5 3.0 2.5 2.0 1.5 1.0 0.5 0.0

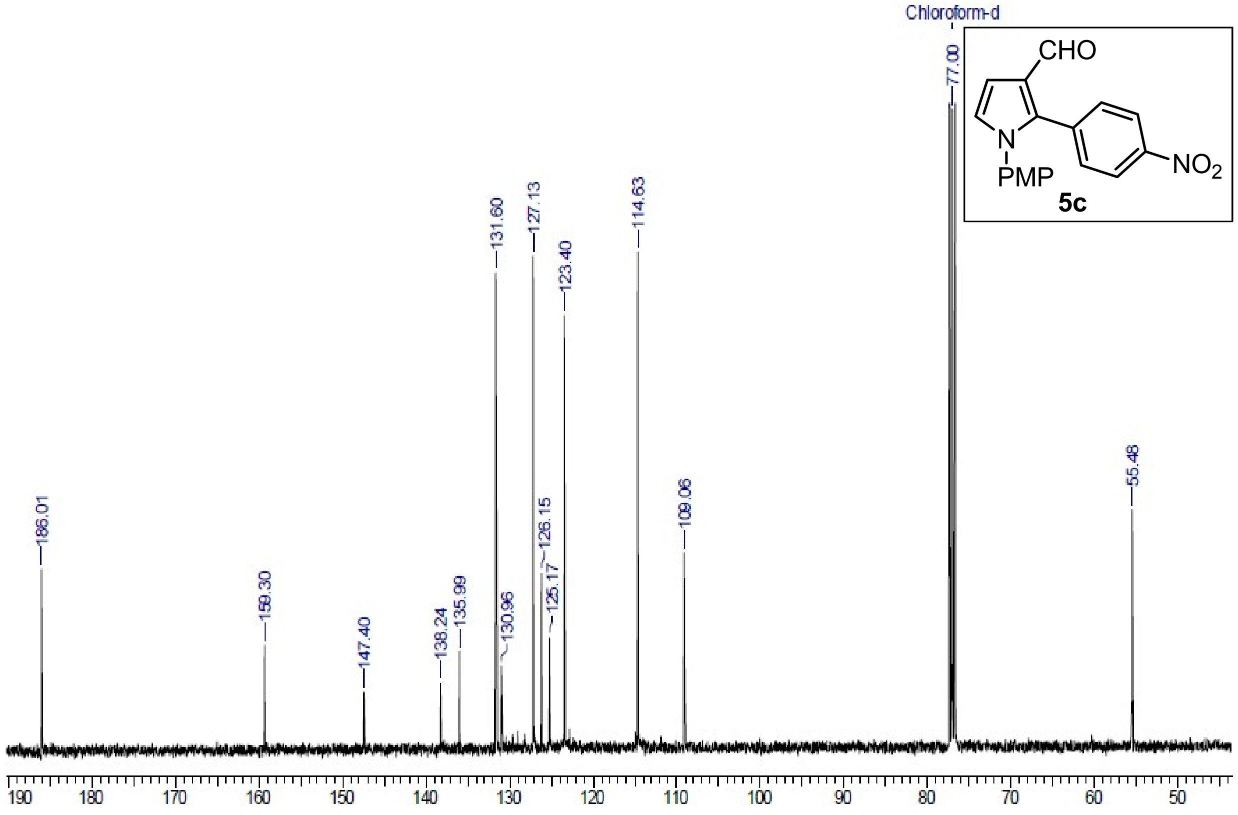

Chloroform-d
77.00
CHO
N
PMP
NO_2
5c
186.01
159.30
147.40
138.24
135.99
131.60
130.96
127.13
126.15
125.17
123.40
114.63
109.06
55.48
190 180 170 160 150 140 130 120 110 100 90 80 70 60 50

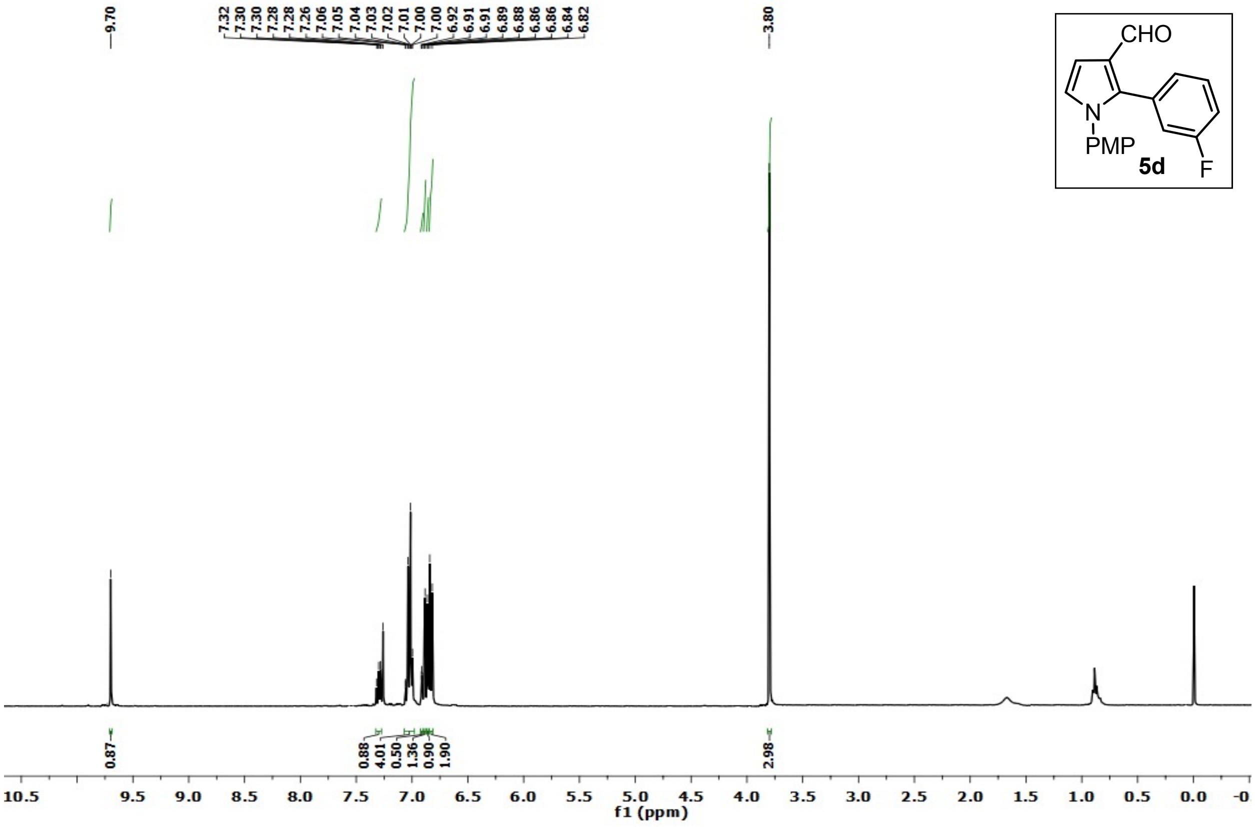
9.70
7.32
7.30
7.30
7.28
7.28
7.26
7.06
7.05
7.04
7.03
7.02
7.01
7.00
7.00
6.92
6.91
6.91
6.89
6.88
6.86
6.86
6.84
6.82
3.80
CHO
N
PMP
5d
F
0.87
0.88
4.01
0.50
1.36
0.90
1.90
2.98
f1 (ppm)

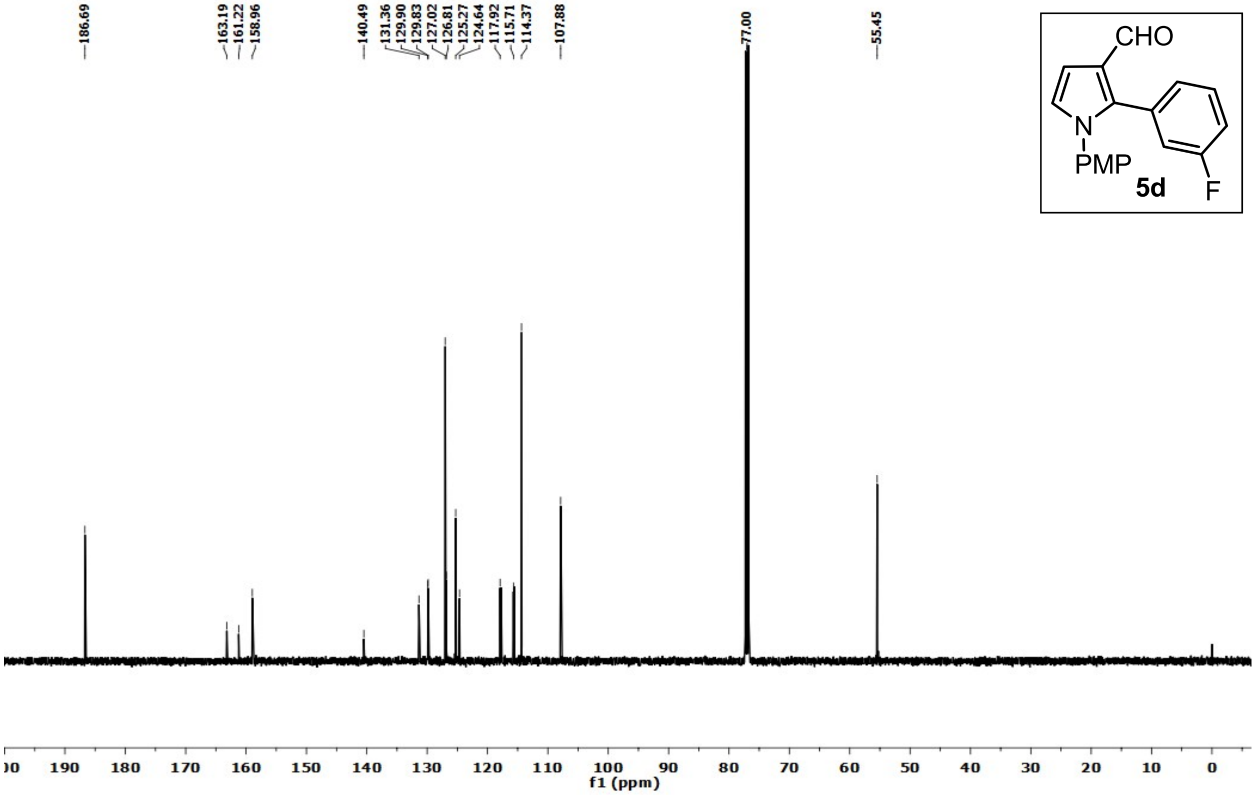
186.69
163.19
161.22
158.96
140.49
131.36
129.90
129.83
127.02
126.81
125.27
124.64
117.92
115.71
114.37
107.88
77.00
55.45
CHO
N
PMP
5d
F
f1 (ppm)

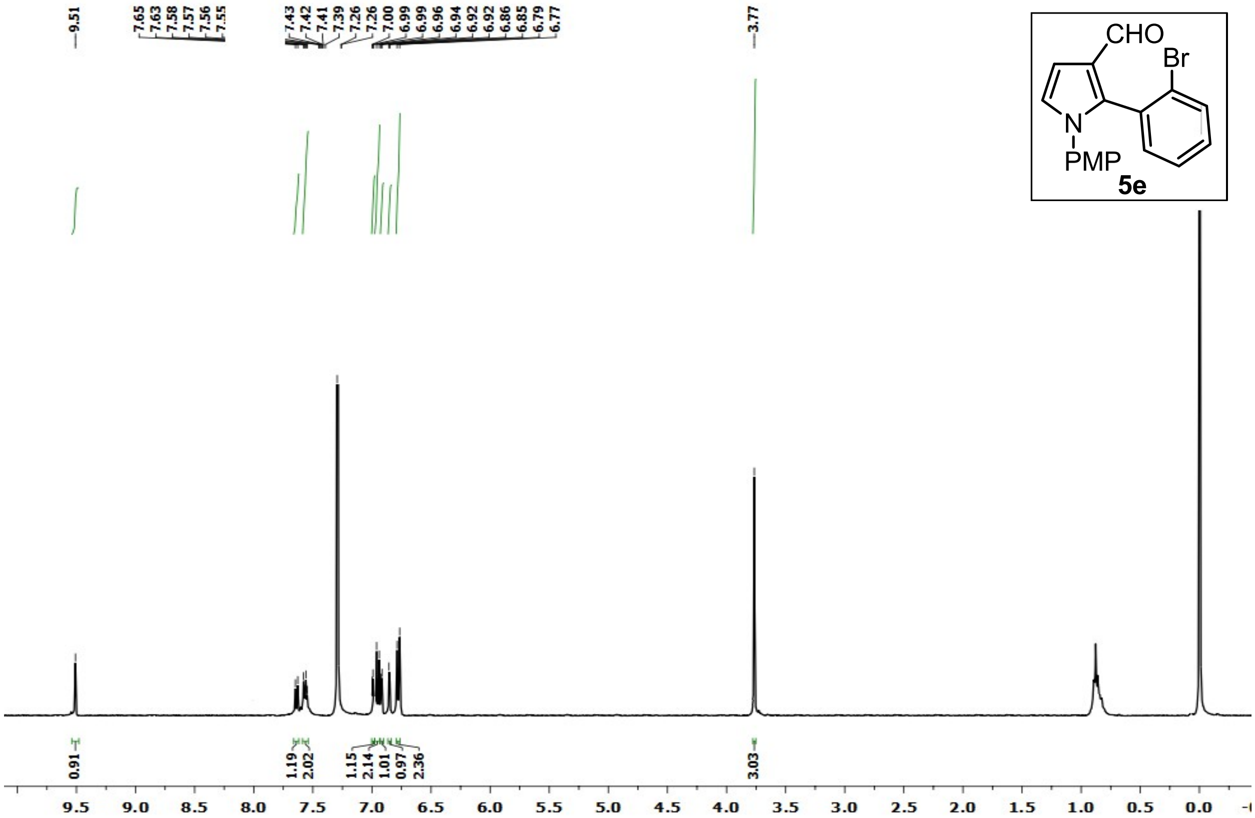
CHO
Br
N
PMP
5e

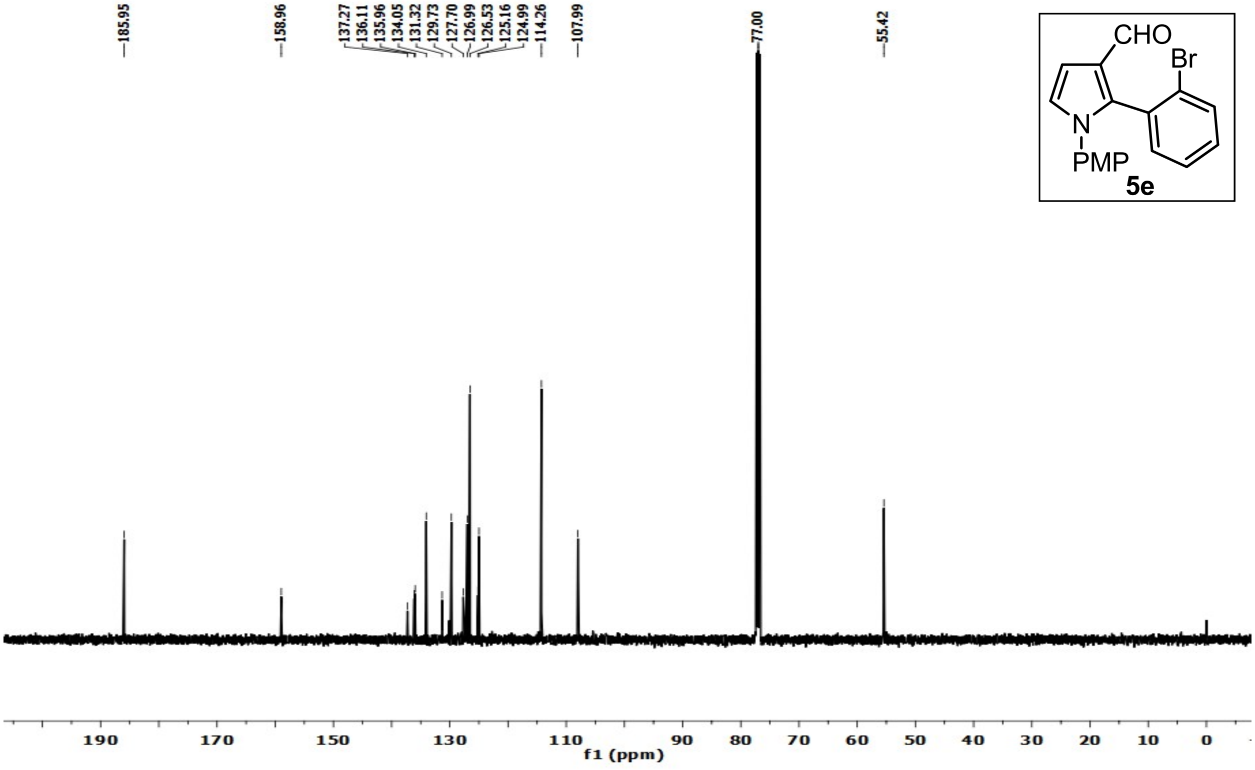
CHO
Br
N
PMP
5e
f1 (ppm)

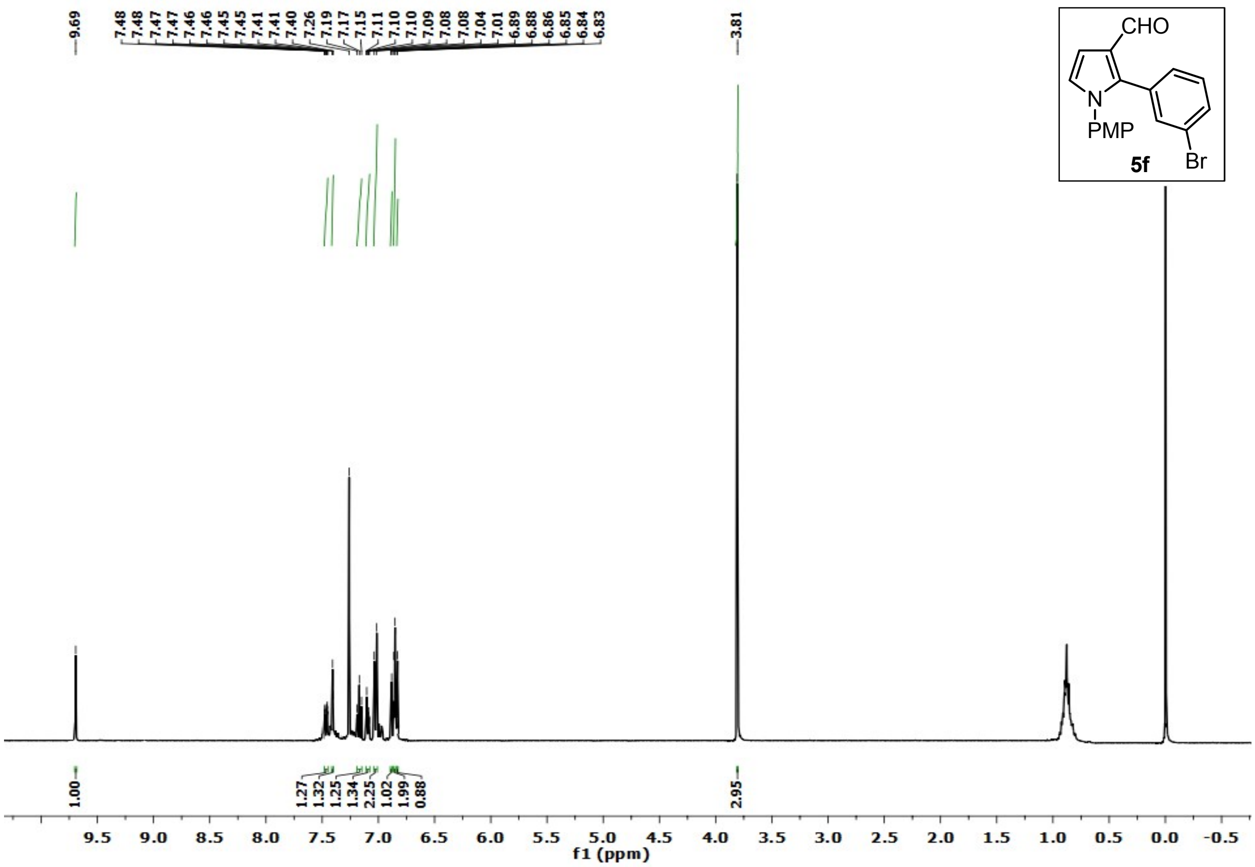
9.69
7.48 7.48 7.47 7.46 7.46 7.45 7.45 7.41 7.41 7.40 7.26 7.19 7.17 7.15 7.11 7.10 7.10 7.09 7.08 7.08 7.04 7.01 6.89 6.88 6.86 6.85 6.84 6.83
3.81
1.00
1.27 1.32 1.25 1.34 2.25 1.02 1.99 0.88
2.95
f1 (ppm)
CHO
N
PMP
Br
5f

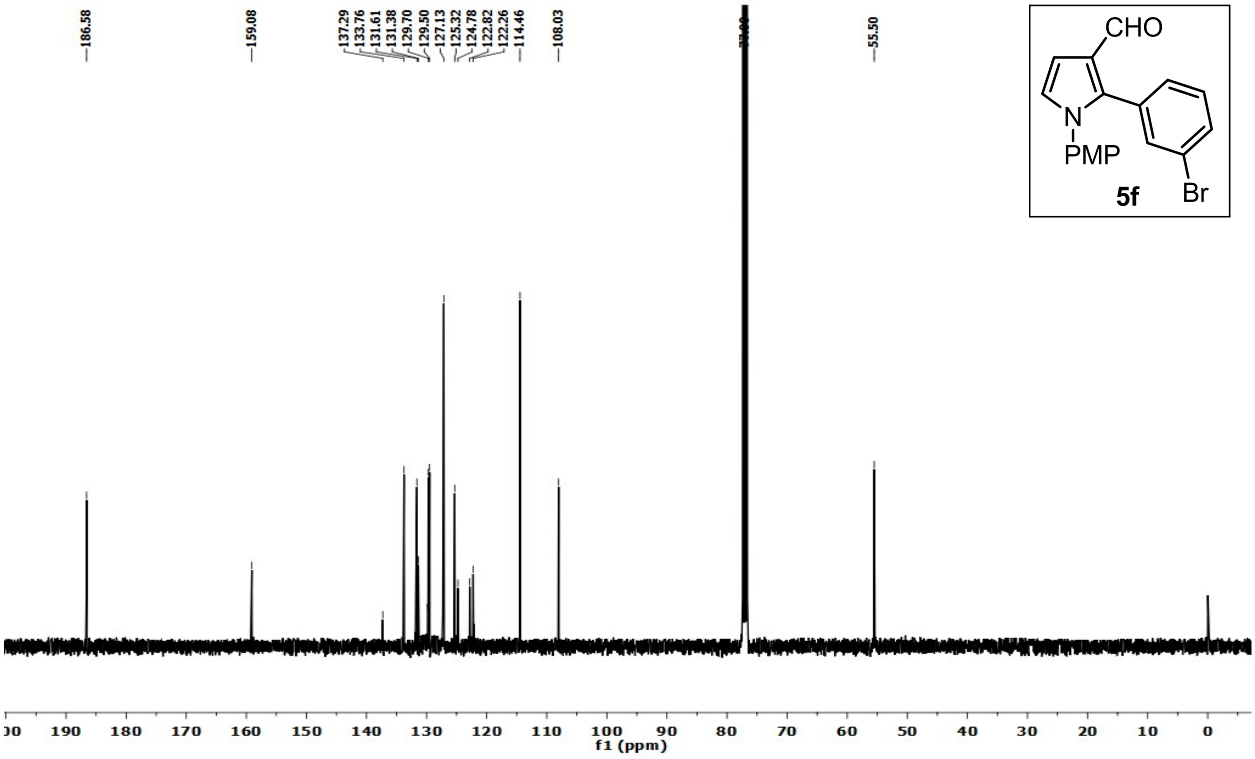
186.58
159.08
137.29 133.76 131.61 131.38 129.70 129.50 127.13 125.32 124.78 122.82 122.26 114.46
108.03
55.50
f1 (ppm)
CHO
N
PMP
Br
5f

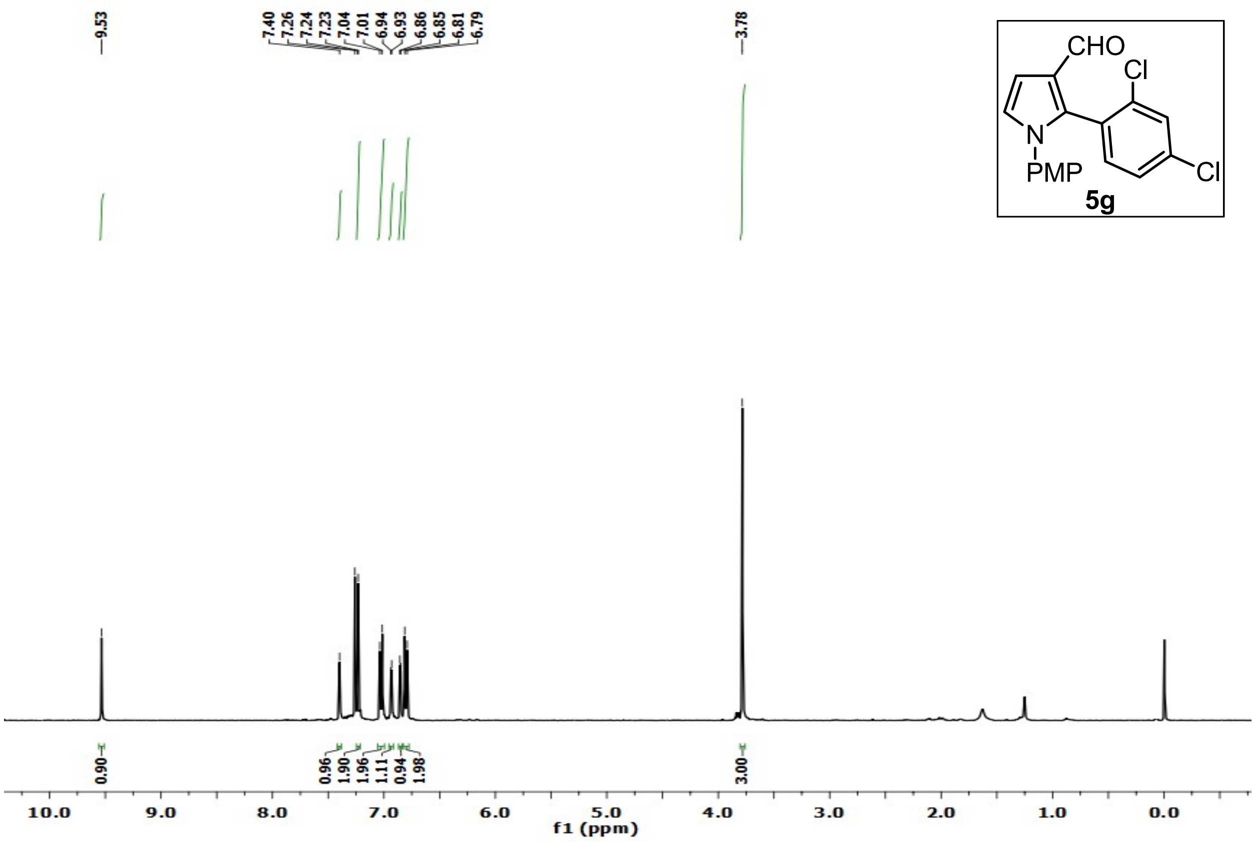
9.53
7.40
7.26
7.24
7.23
7.04
7.01
6.94
6.93
6.86
6.85
6.81
6.79
3.78
CHO
Cl
N
PMP
Cl
5g
0.90
0.96
1.90
1.96
1.11
0.94
1.98
3.00
10.0 9.0 8.0 7.0 6.0 5.0 4.0 3.0 2.0 1.0 0.0
f1 (ppm)

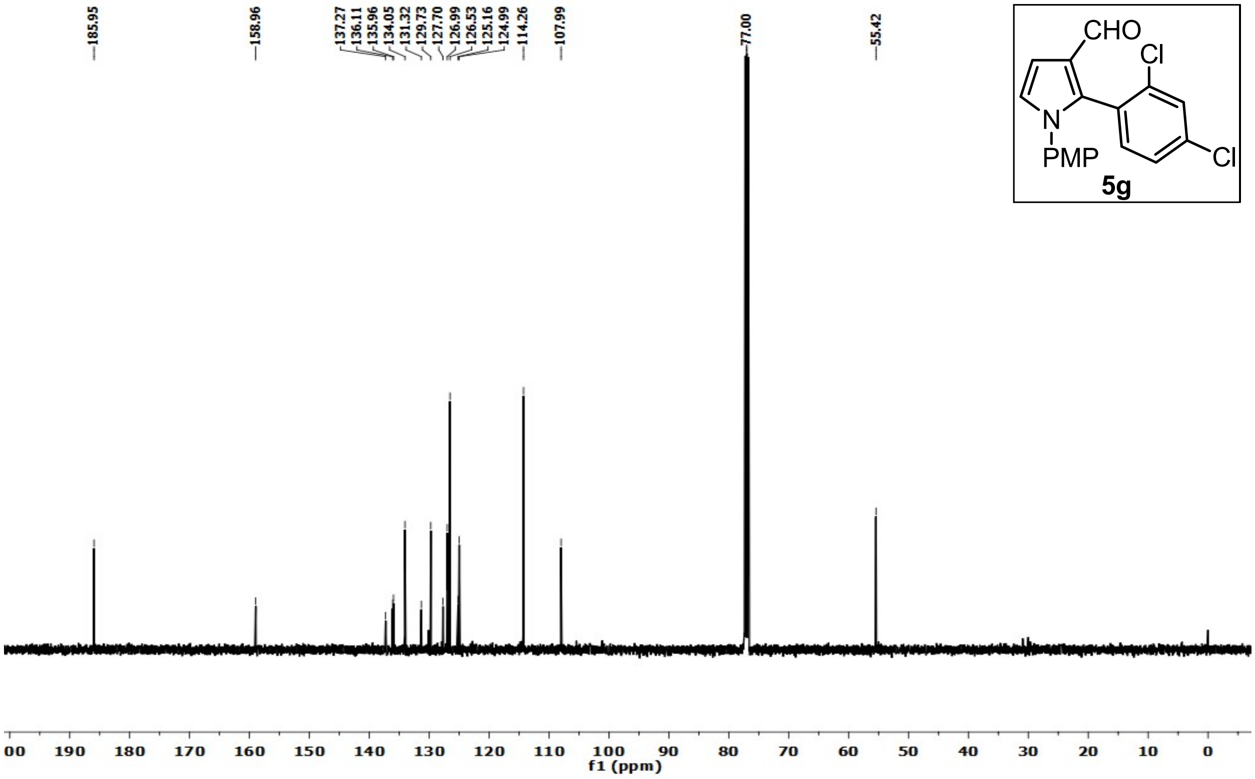
185.95
158.96
137.27
136.11
135.96
134.05
131.32
129.73
127.70
126.99
126.53
125.16
124.99
114.26
107.99
77.00
55.42
CHO
Cl
N
PMP
Cl
5g
00 190 180 170 160 150 140 130 120 110 100 90 80 70 60 50 40 30 20 10 0
f1 (ppm)

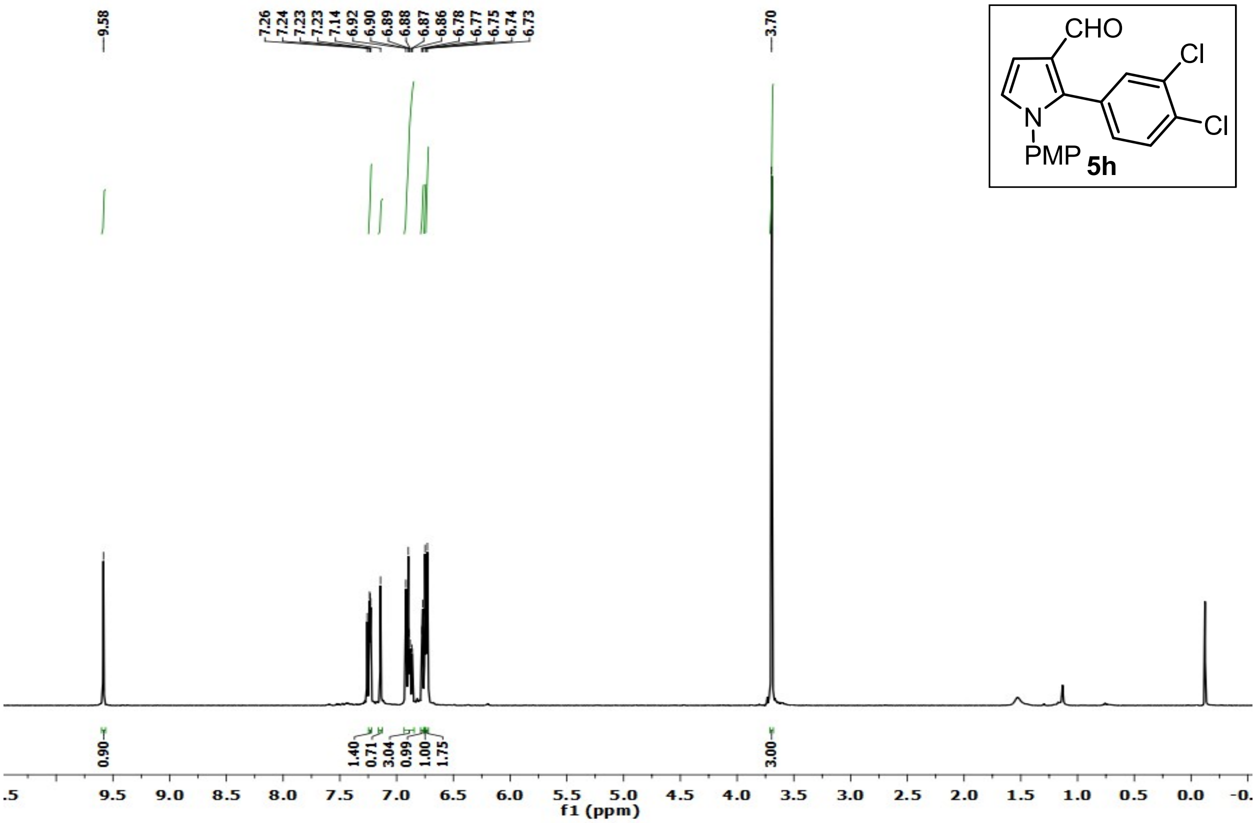
9.58
7.26
7.24
7.23
7.23
7.14
6.92
6.90
6.89
6.88
6.87
6.86
6.78
6.77
6.75
6.74
6.73
3.70
0.90
1.40
0.71
3.04
0.99
1.00
1.75
3.00
.5 9.5 9.0 8.5 8.0 7.5 7.0 6.5 6.0 5.5 5.0 4.5 4.0 3.5 3.0 2.5 2.0 1.5 1.0 0.5 0.0 -0.
f1 (ppm)
CHO
Cl
Cl
N
PMP
5h

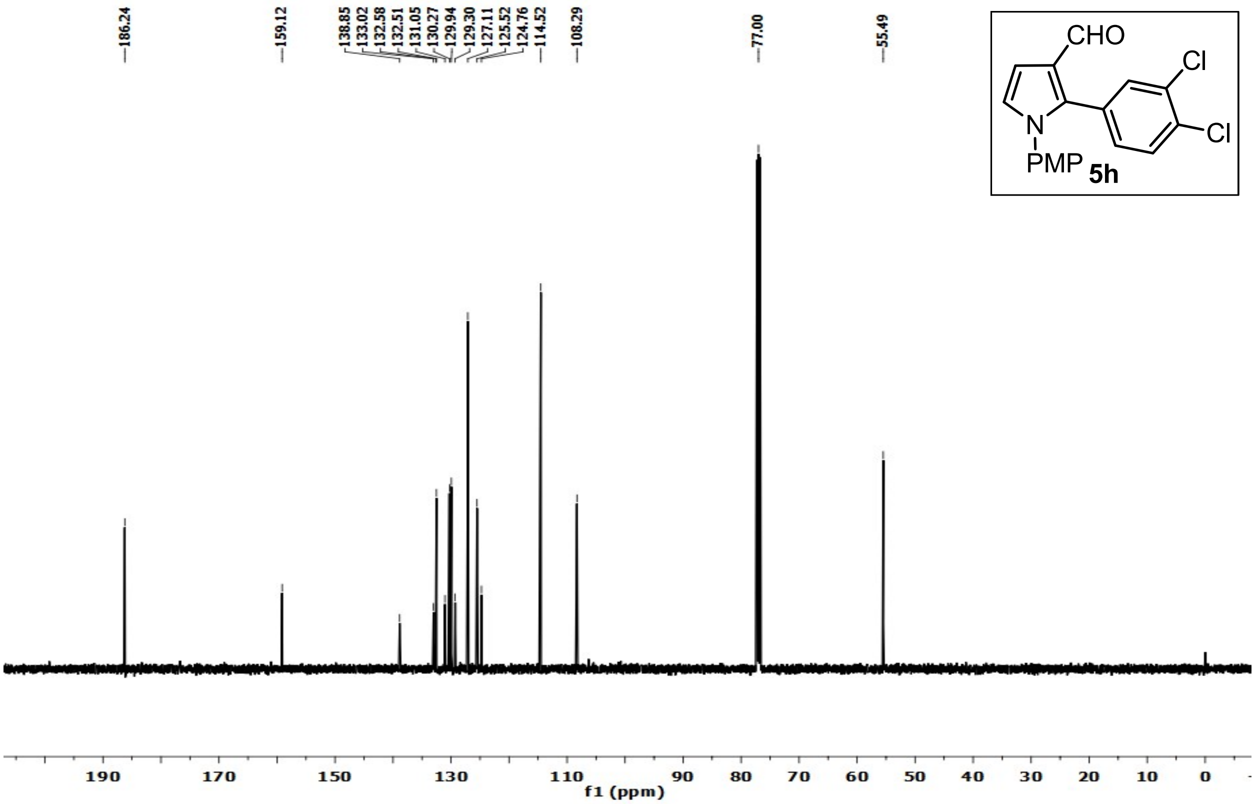
186.24
159.12
138.85
133.02
132.58
132.51
131.05
130.27
129.94
129.30
127.11
125.52
124.76
114.52
108.29
77.00
55.49
190 170 150 130 110 90 80 70 60 50 40 30 20 10 0
f1 (ppm)
CHO
Cl
Cl
N
PMP
5h

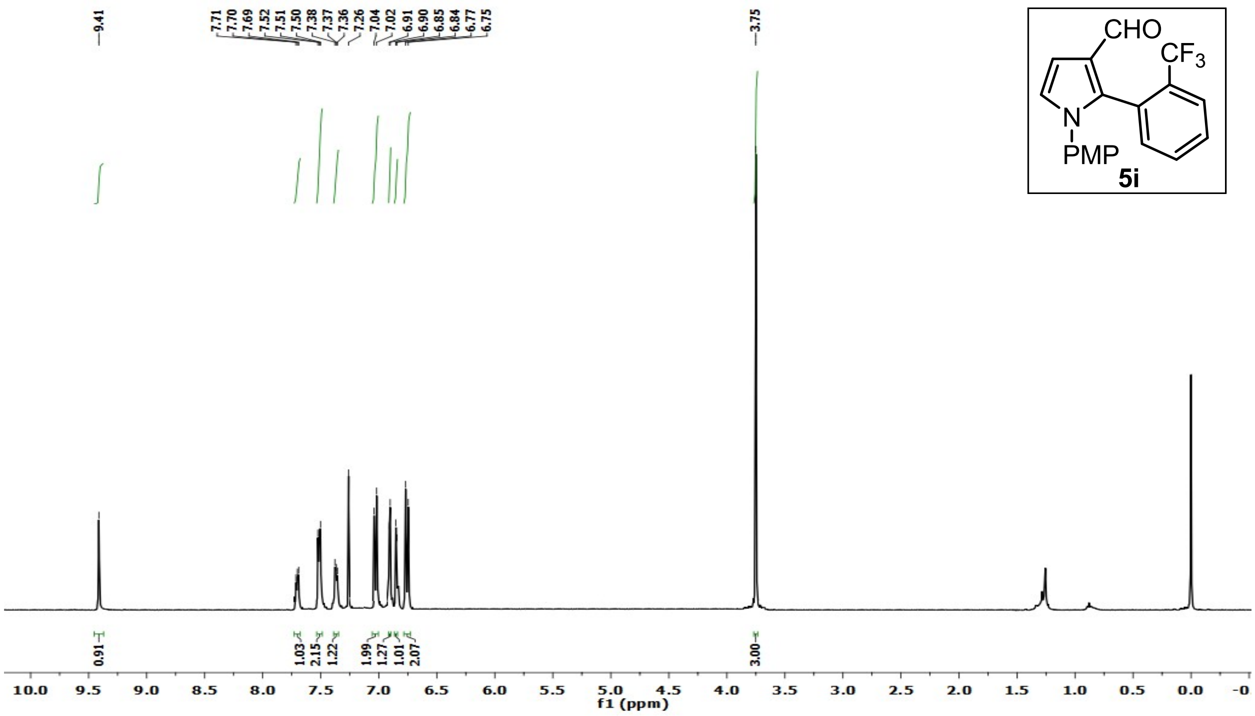
9.41
7.71
7.70
7.69
7.52
7.51
7.50
7.38
7.37
7.36
7.26
7.04
7.02
6.91
6.90
6.85
6.84
6.77
6.75
3.75
0.91
1.03
2.15
1.22
1.99
1.27
1.01
2.07
3.00
f1 (ppm)
CHO
CF_3
N
PMP
5i

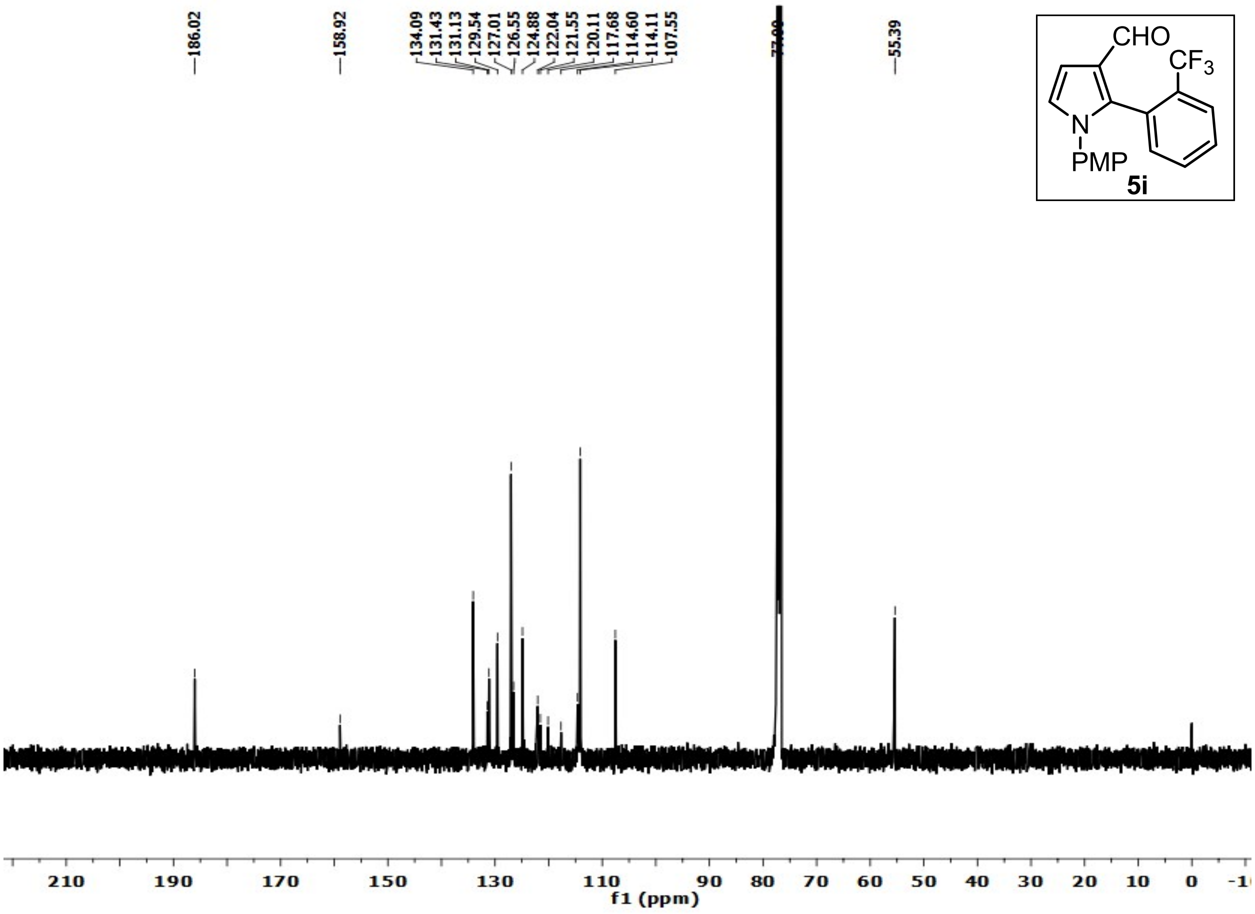
186.02
158.92
134.09
131.43
131.13
129.54
127.01
126.55
124.88
122.04
121.55
120.11
117.68
114.60
114.11
107.55
55.39
f1 (ppm)
CHO
CF_3
N
PMP
5i

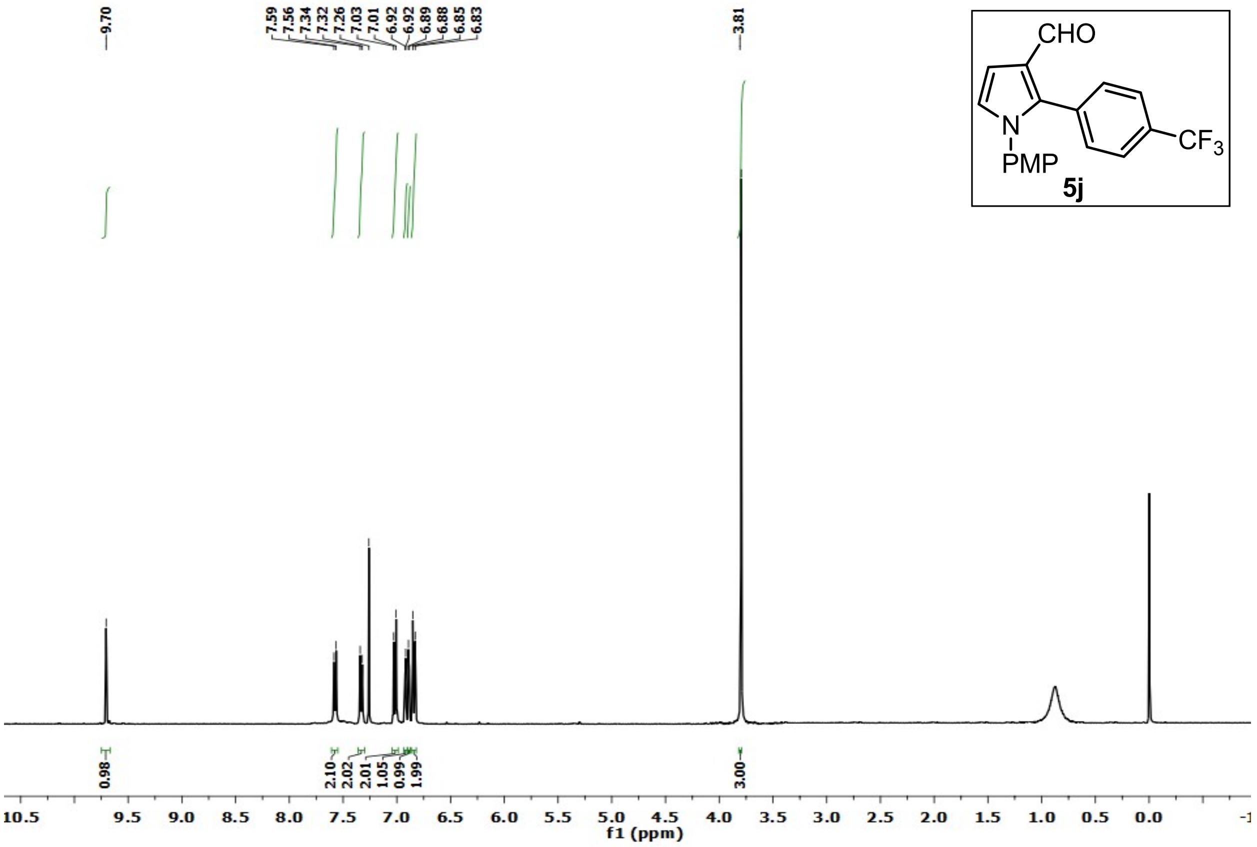
9.70
7.59 7.56 7.34 7.32 7.26 7.03 7.01 6.92 6.92 6.89 6.88 6.85 6.83
3.81
CHO
N
PMP
CF3
5j
0.98
2.10 2.02 2.01 1.05 0.99 1.99
3.00
10.5 9.5 9.0 8.5 8.0 7.5 7.0 6.5 6.0 5.5 5.0 4.5 4.0 3.5 3.0 2.5 2.0 1.5 1.0 0.5 0.0
f1 (ppm)

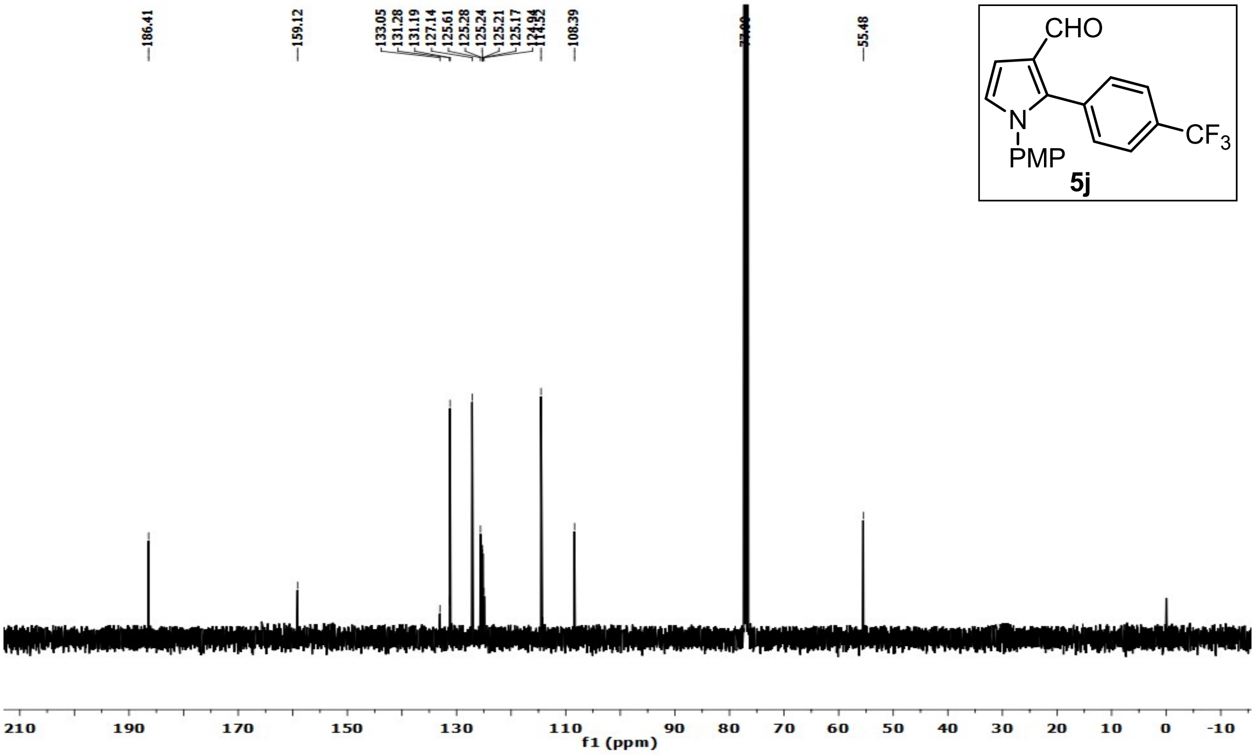
186.41
159.12
133.05 131.28 131.19 127.14 125.61 125.28 125.24 125.21 125.17 114.52
108.39
55.48
CHO
N
PMP
CF3
5j
210 190 170 150 130 110 90 80 70 60 50 40 30 20 10 0 -10
f1 (ppm)

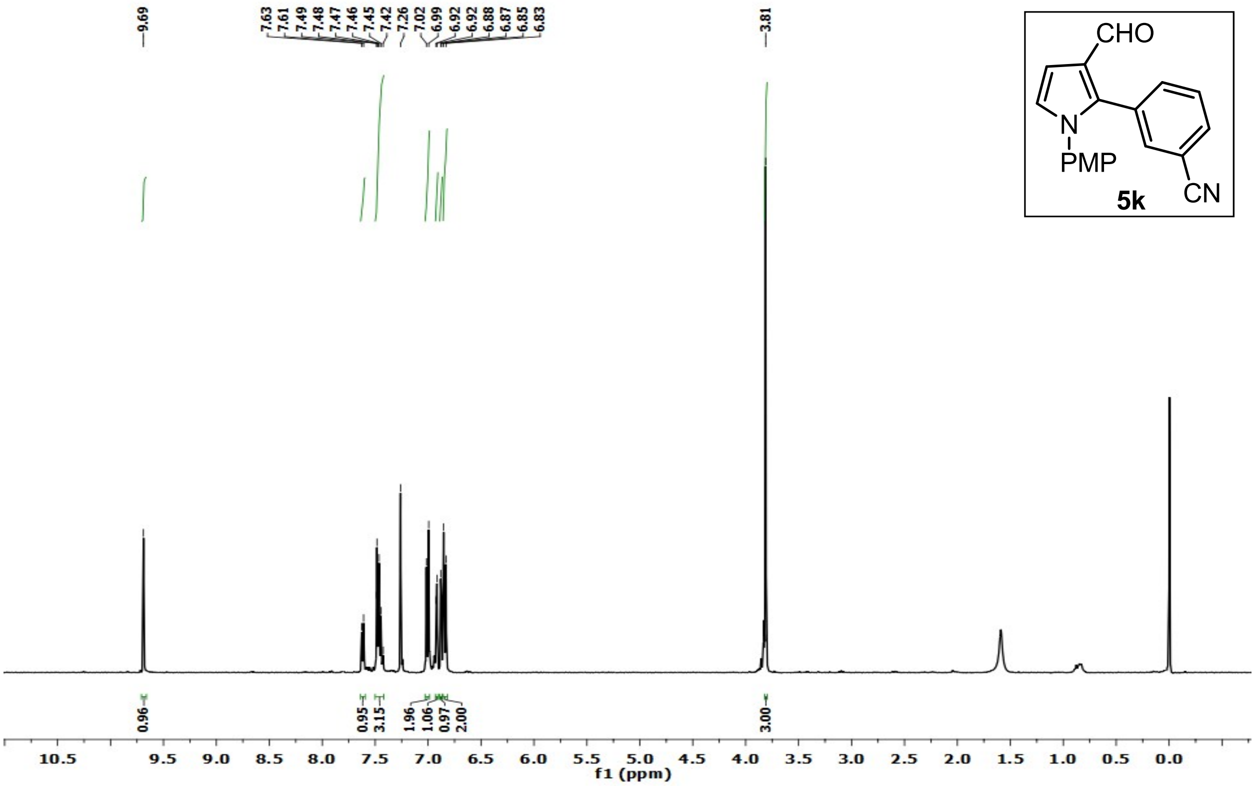
9.69
7.63 7.61 7.49 7.48 7.47 7.46 7.45 7.42 7.26 7.02 6.99 6.92 6.92 6.88 6.87 6.85 6.83
3.81
CHO
N
PMP
CN
5k
0.96
0.95 3.15
1.96 1.06 0.97 2.00
3.00
10.5 9.5 9.0 8.5 8.0 7.5 7.0 6.5 6.0 5.5 5.0 4.5 4.0 3.5 3.0 2.5 2.0 1.5 1.0 0.5 0.0
f1 (ppm)

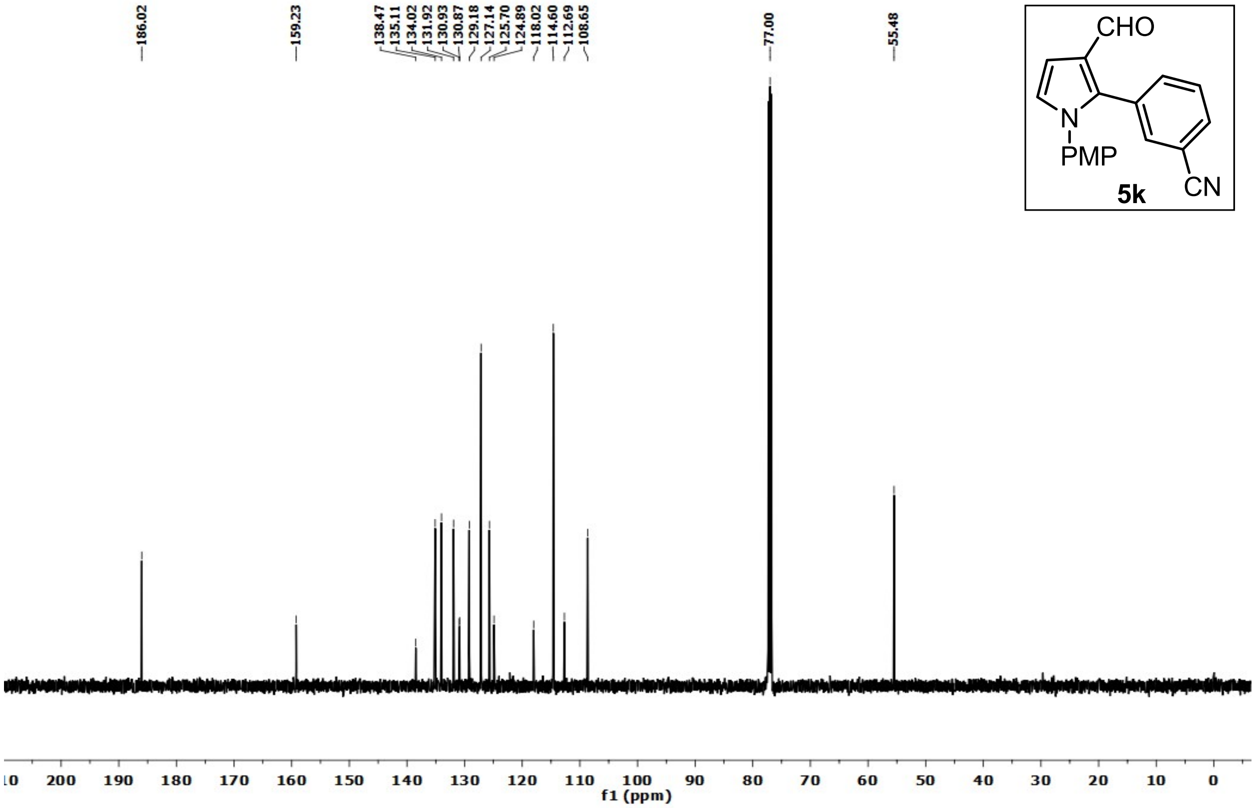
186.02
159.23
138.47 135.11 134.02 131.92 130.93 130.87 129.18 127.14 125.70 124.89 118.02 114.60 112.69 108.65
77.00
55.48
CHO
N
PMP
CN
5k
10 200 190 180 170 160 150 140 130 120 110 100 90 80 70 60 50 40 30 20 10 0
f1 (ppm)

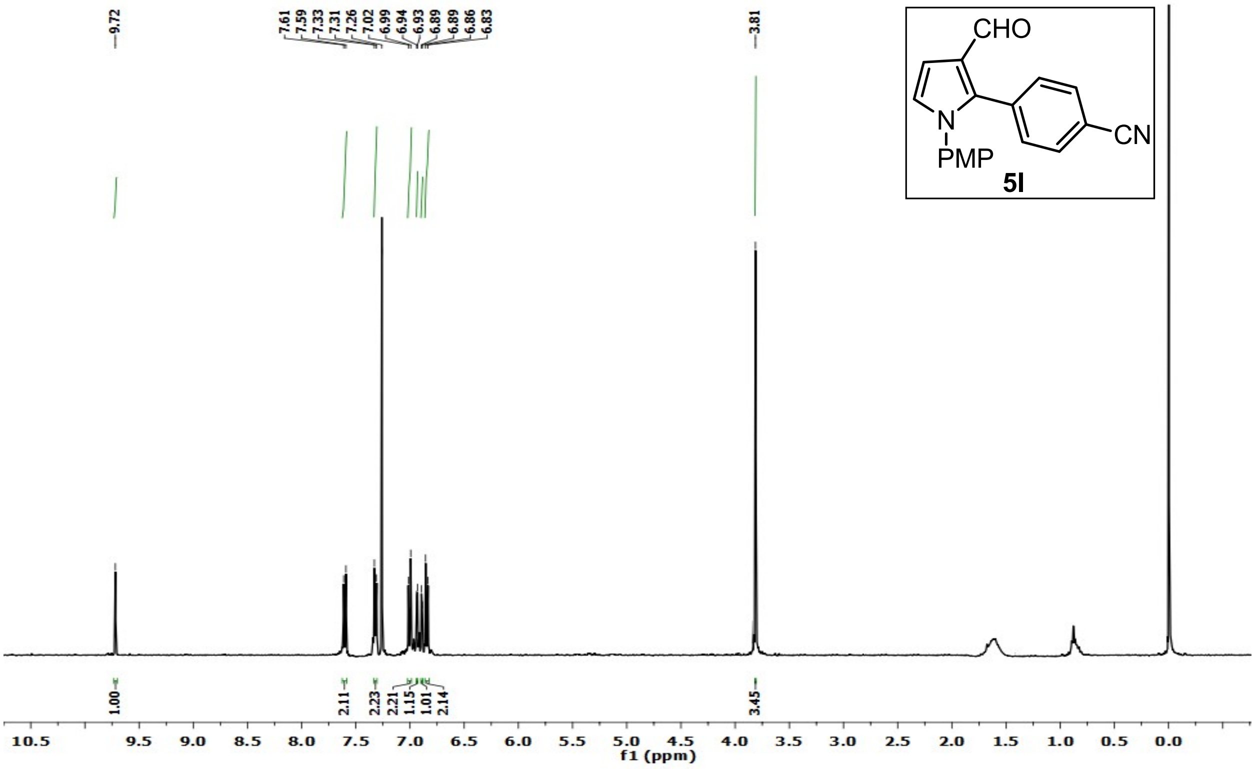
9.72
7.61
7.59
7.33
7.31
7.26
7.02
6.99
6.94
6.93
6.89
6.89
6.86
6.83
3.81
CHO
N
PMP
CN
5l
1.00
2.11
2.23
2.21
1.15
1.01
2.14
3.45
f1 (ppm)

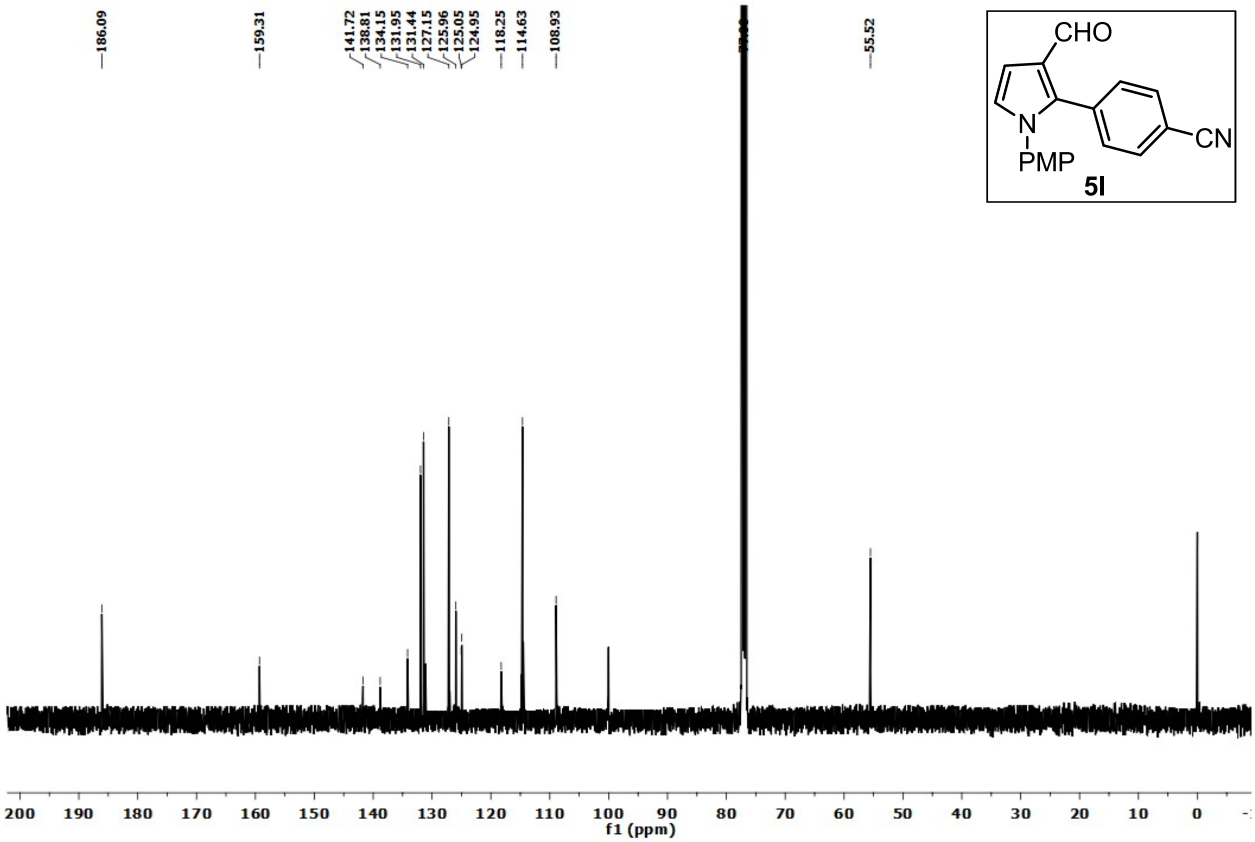
186.09
159.31
141.72
138.81
134.15
131.95
131.44
127.15
125.96
125.05
124.95
118.25
114.63
108.93
55.52
CHO
N
PMP
CN
5l
f1 (ppm)

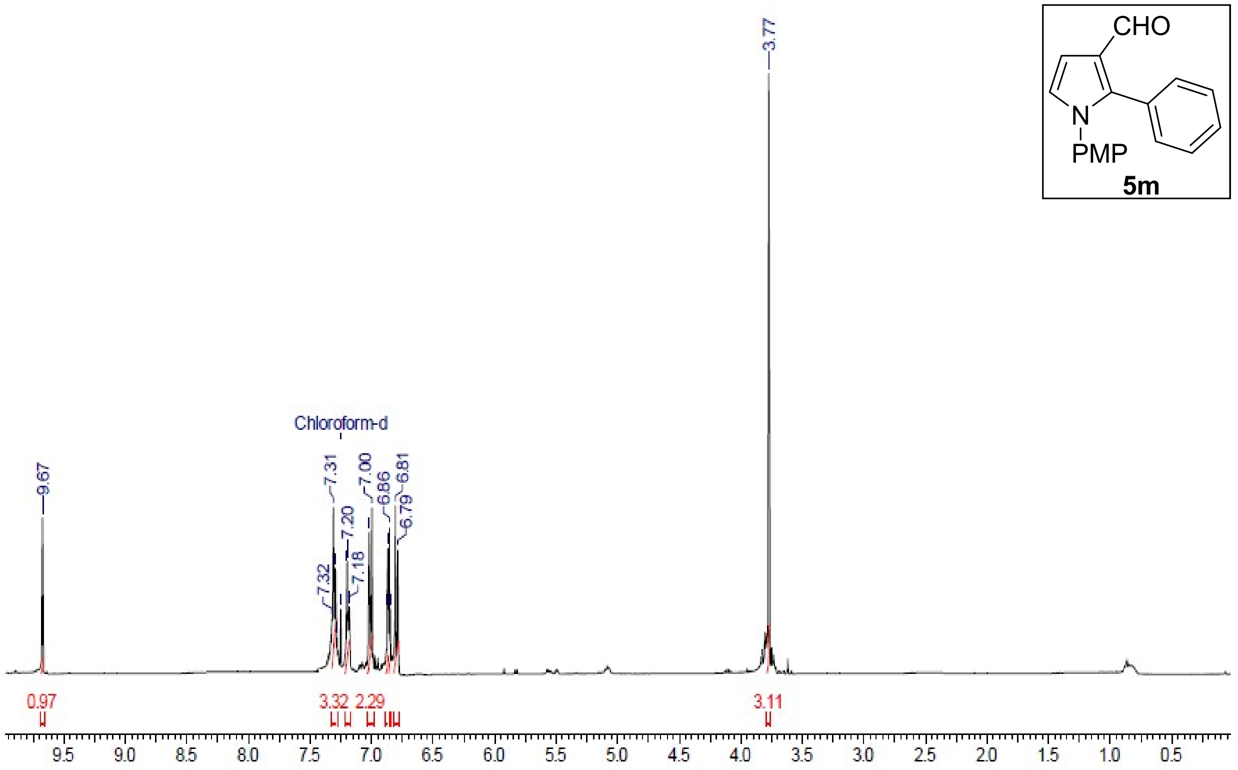
CHO
N
PMP
5m
Chloroform-d
9.67
7.32
7.31
7.20
7.18
7.00
6.86
6.81
6.79
3.77
0.97
3.32
2.29
3.11
9.5 9.0 8.5 8.0 7.5 7.0 6.5 6.0 5.5 5.0 4.5 4.0 3.5 3.0 2.5 2.0 1.5 1.0 0.5

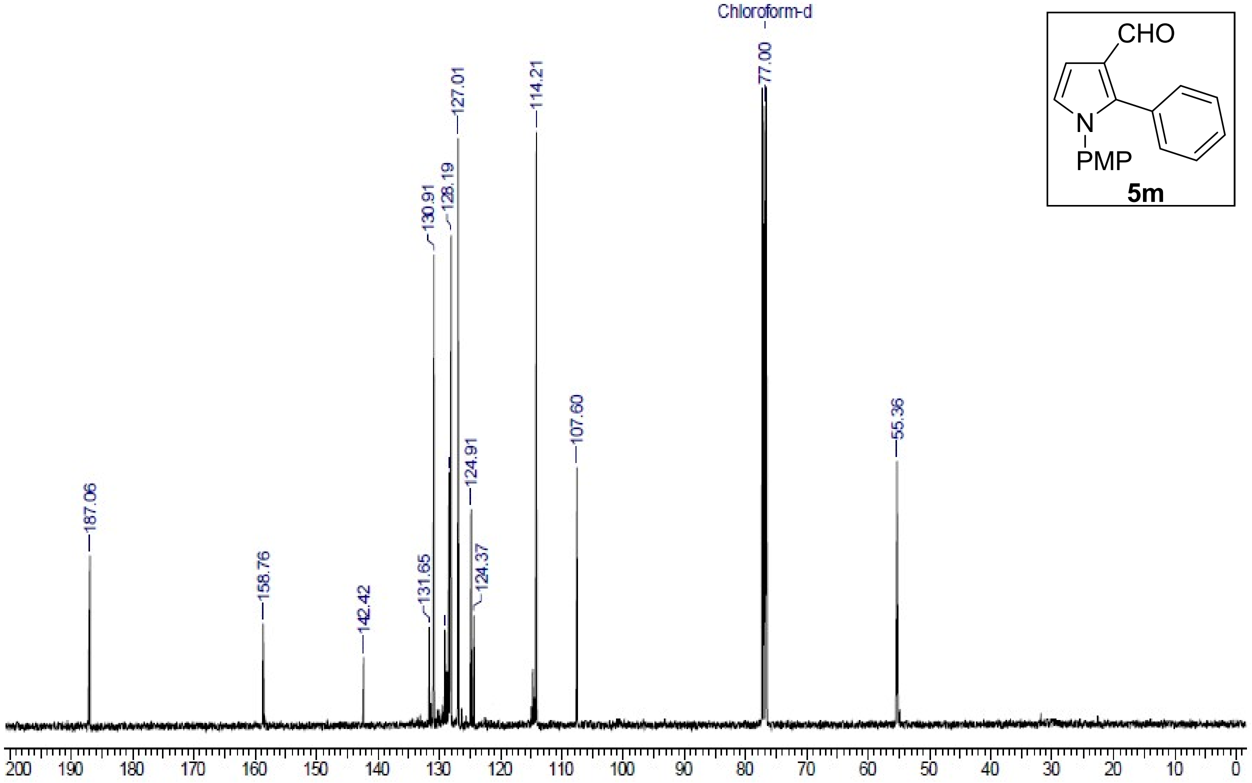
CHO
N
PMP
5m
Chloroform-d
77.00
187.06
158.76
142.42
131.65
130.91
128.19
127.01
124.91
124.37
114.21
107.60
55.36
200 190 180 170 160 150 140 130 120 110 100 90 80 70 60 50 40 30 20 10 0

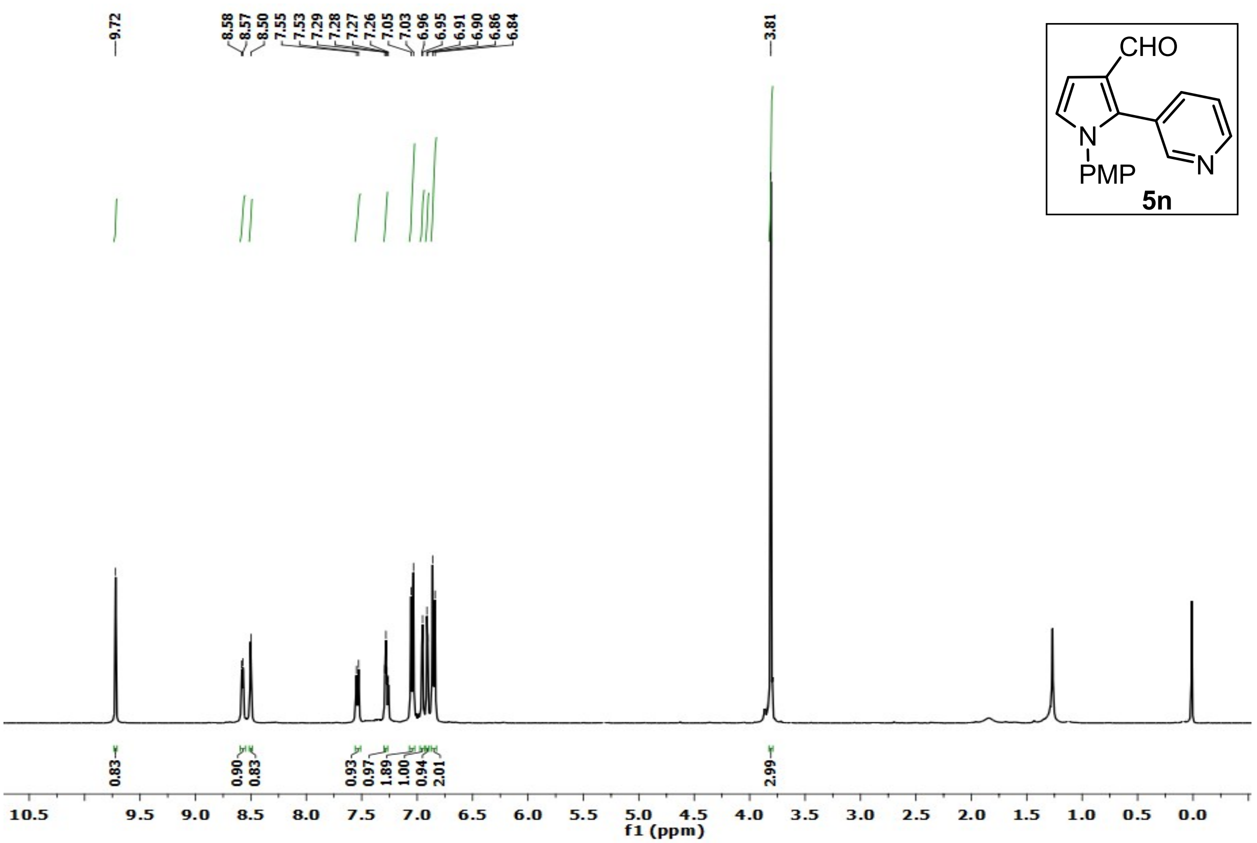
9.72
8.58
8.57
8.50
7.55
7.53
7.29
7.28
7.27
7.26
7.05
7.03
6.96
6.95
6.91
6.90
6.86
6.84
3.81
0.83
0.90
0.83
0.93
0.97
1.89
1.00
0.94
2.01
2.99
f1 (ppm)
CHO
N
PMP
N
5n

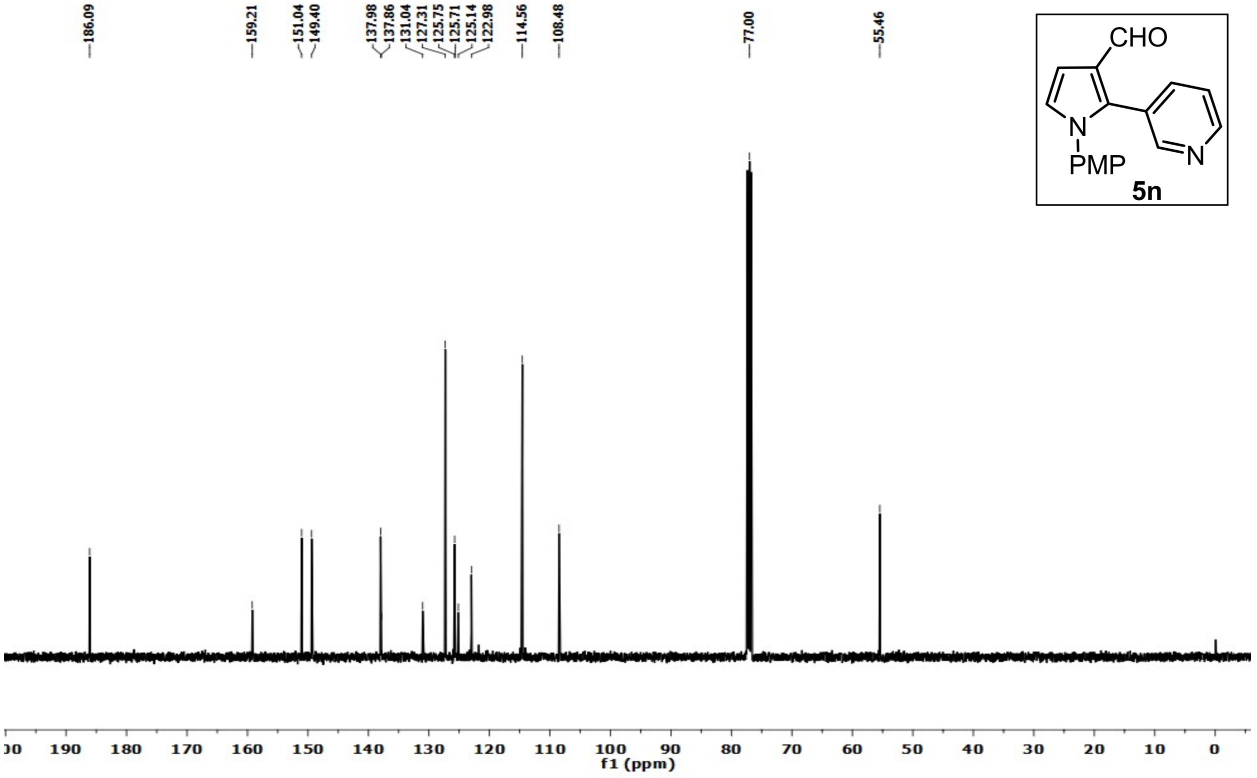
186.09
159.21
151.04
149.40
137.98
137.86
131.04
127.31
125.75
125.71
125.14
122.98
114.56
108.48
77.00
55.46
f1 (ppm)
CHO
N
PMP
N
5n

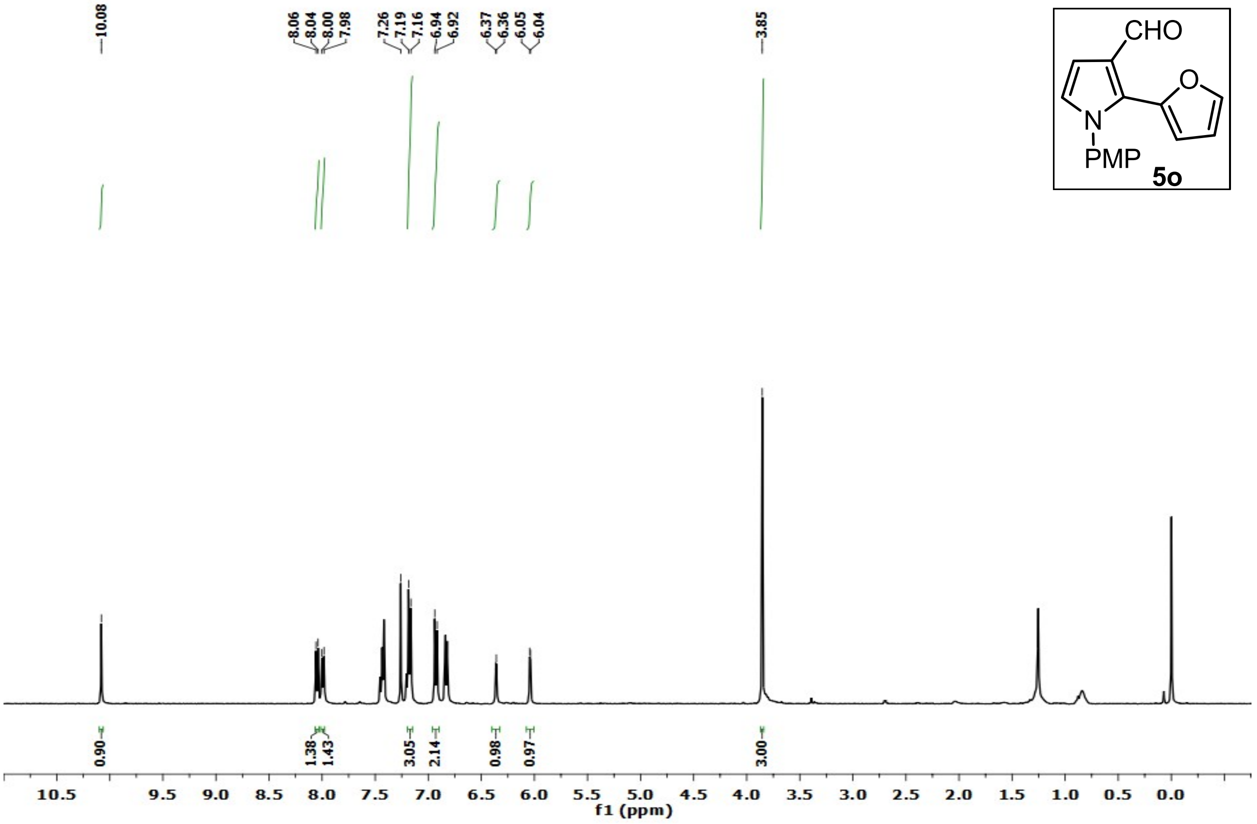
10.08
8.06
8.04
8.00
7.98
7.26
7.19
7.16
6.94
6.92
6.37
6.36
6.05
6.04
3.85
CHO
N
O
PMP
5o
0.90
1.38
1.43
3.05
2.14
0.98
0.97
3.00
f1 (ppm)

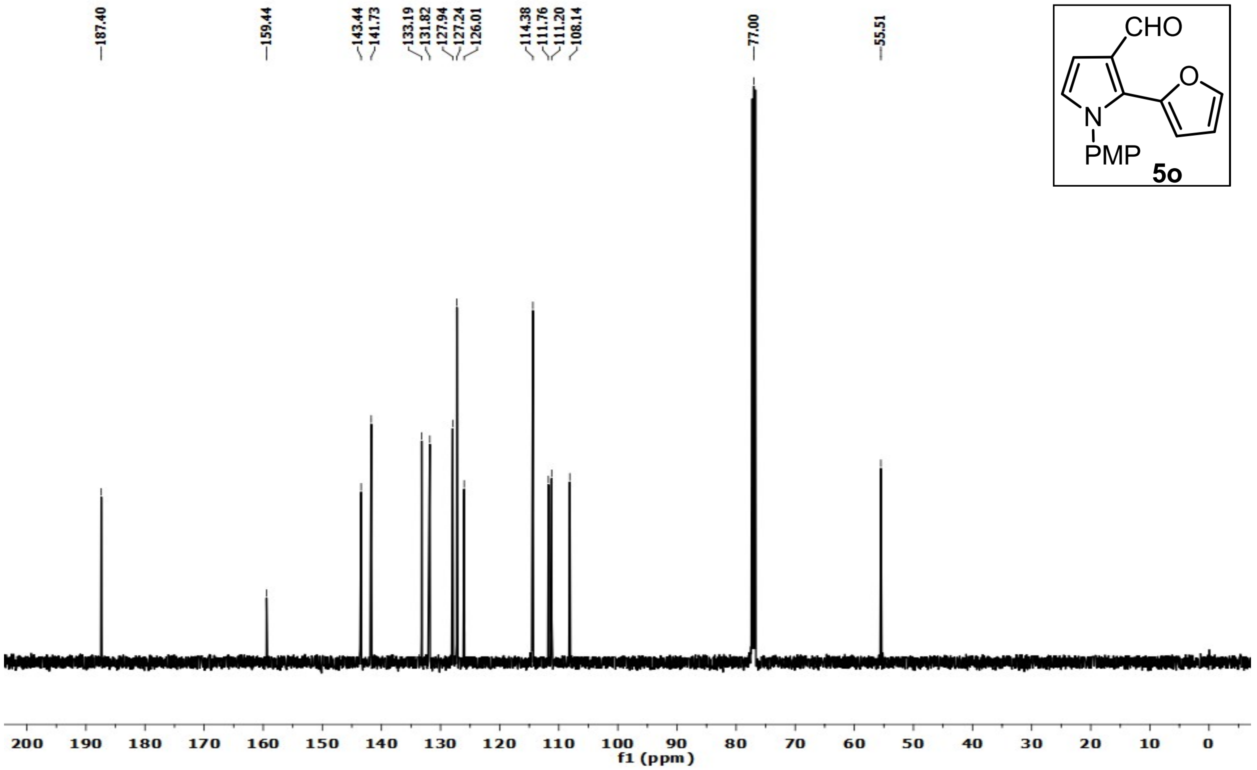
187.40
159.44
143.44
141.73
133.19
131.82
127.94
127.24
126.01
114.38
111.76
111.20
108.14
77.00
55.51
CHO
N
O
PMP
5o
f1 (ppm)

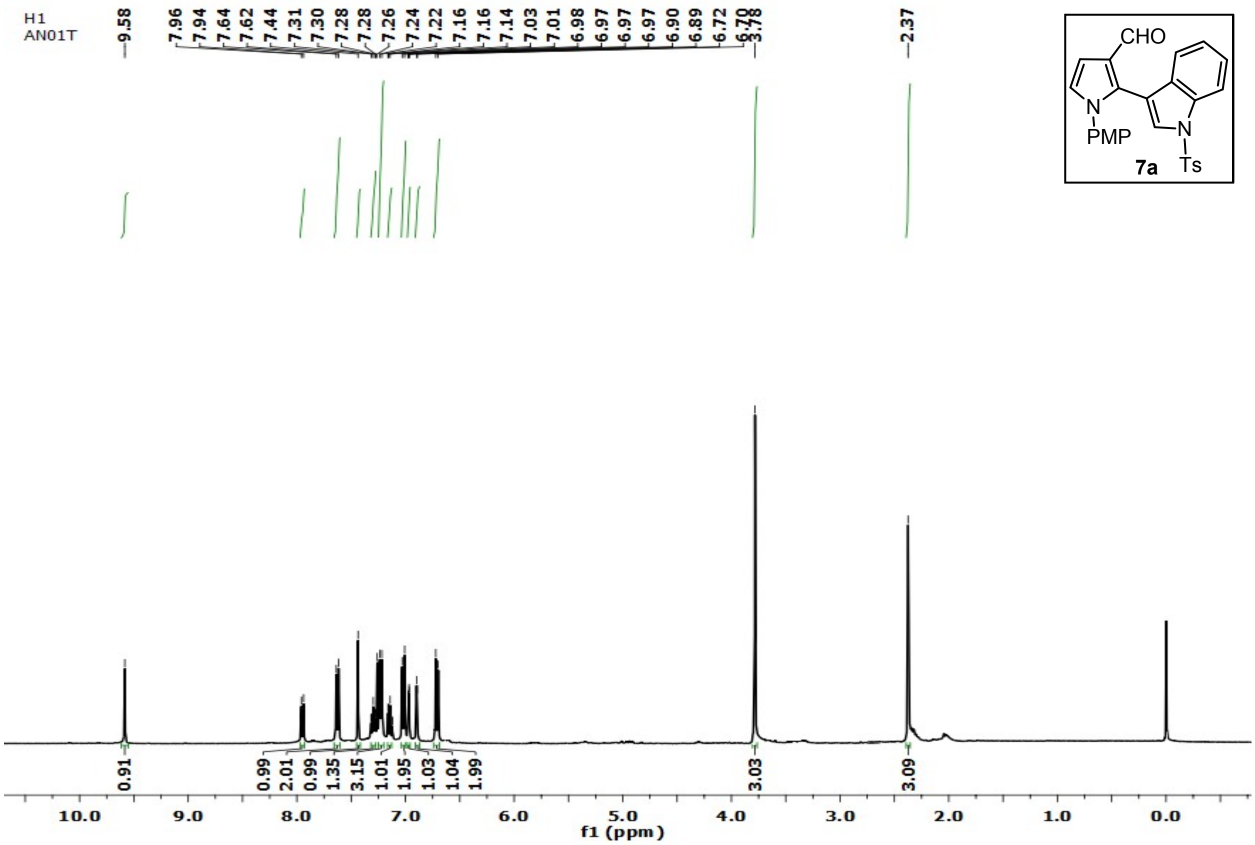
H1
AN01T
9.58
7.96 7.94 7.64 7.62 7.44 7.31 7.30 7.28 7.28 7.26 7.24 7.22 7.16 7.16 7.14 7.03 7.01 6.98 6.97 6.97 6.97 6.90 6.89 6.72 6.70
3.78
2.37
CHO
N
PMP
N
Ts
7a
0.91
0.99 2.01 0.99 1.35 3.15 1.01 1.95 1.03 1.04 1.99
3.03
3.09
10.0 9.0 8.0 7.0 6.0 5.0 4.0 3.0 2.0 1.0 0.0
f1 (ppm)

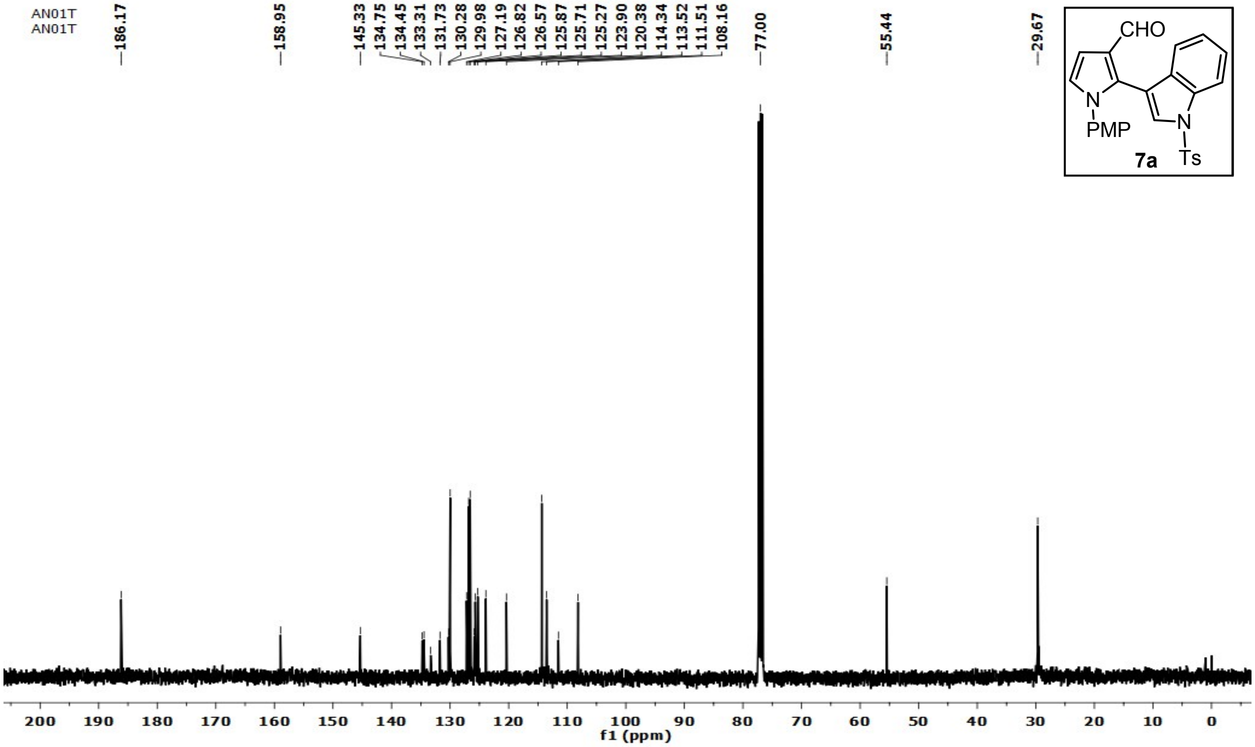
AN01T
AN01T
186.17
158.95
145.33 134.75 134.45 133.31 131.73 130.28 129.98 127.19 126.82 126.57 125.87 125.71 125.27 123.90 120.38 114.34 113.52 111.51 108.16
77.00
55.44
29.67
CHO
N
PMP
N
Ts
7a
200 190 180 170 160 150 140 130 120 110 100 90 80 70 60 50 40 30 20 10 0
f1 (ppm)

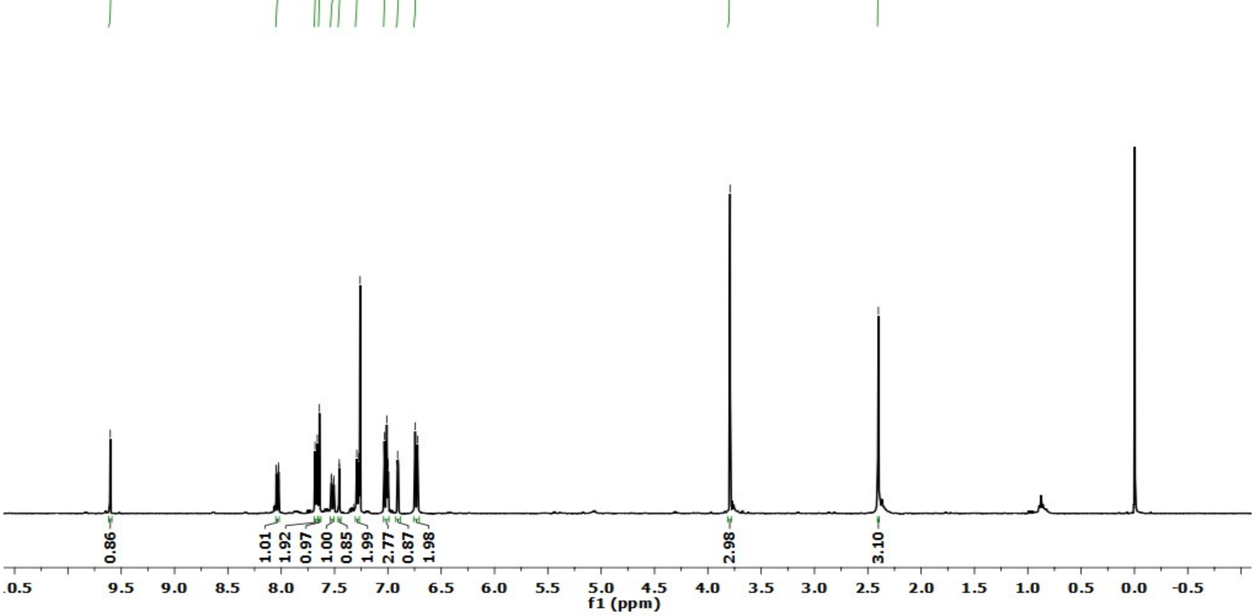
H1
ANO2T
9.60
8.05
8.03
8.02
7.68
7.66
7.64
7.53
7.53
7.51
7.51
7.46
7.45
7.29
7.27
7.26
7.03
7.01
7.01
7.01
7.00
7.00
6.91
6.90
6.74
6.72
3.79
2.40
0.86
1.01
1.92
0.97
1.00
0.85
1.99
2.77
0.87
1.98
2.98
3.10
.0.5 9.5 9.0 8.5 8.0 7.5 7.0 6.5 6.0 5.5 5.0 4.5 4.0 3.5 3.0 2.5 2.0 1.5 1.0 0.5 0.0 -0.5
f1 (ppm)

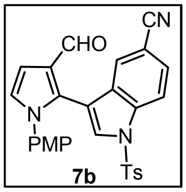
CN
CHO
N
PMP
N
Ts
7b

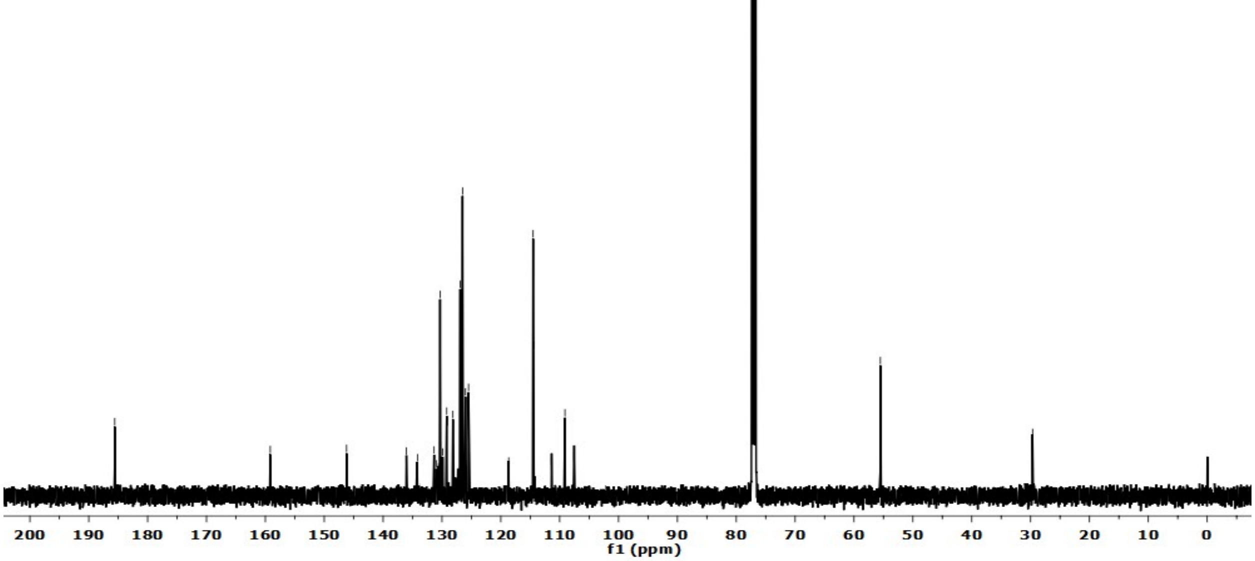
AN02T
ANO2T
185.59
159.17
146.20
136.06
134.14
131.33
130.98
130.32
129.88
129.19
128.14
126.90
126.50
126.00
125.48
118.66
114.51
114.44
111.41
109.10
107.57
77.00
55.52
29.66
200 190 180 170 160 150 140 130 120 110 100 90 80 70 60 50 40 30 20 10 0
f1 (ppm)

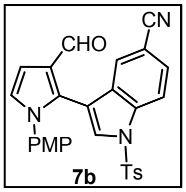
CN
CHO
N
PMP
N
Ts
7b

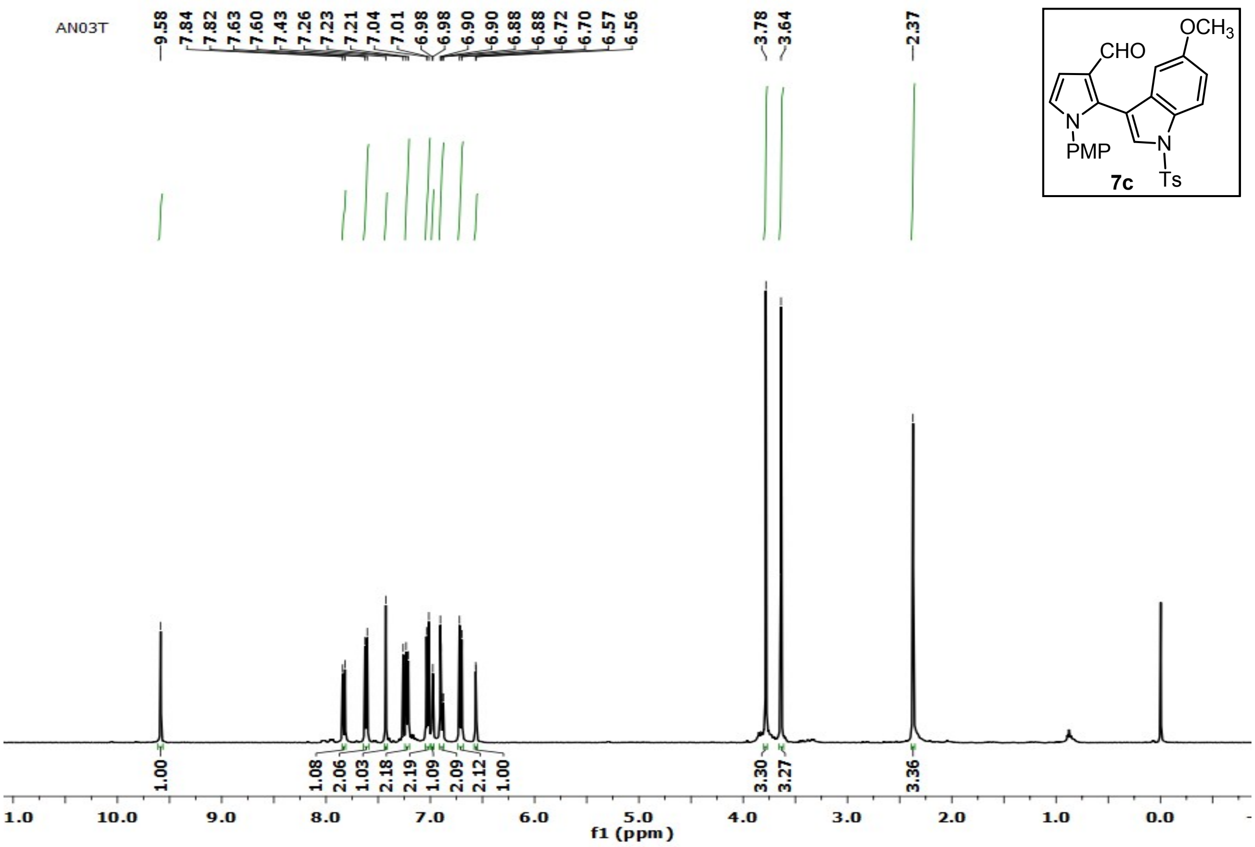
AN03T
9.58
7.84
7.82
7.63
7.60
7.43
7.26
7.23
7.21
7.04
7.01
6.98
6.98
6.90
6.90
6.88
6.88
6.72
6.70
6.57
6.56
3.78
3.64
2.37
1.00
1.08
2.06
1.03
2.18
2.19
1.09
2.09
2.12
1.00
3.30
3.27
3.36
f1 (ppm)
CHO
OCH_3
N
PMP
N
Ts
7c

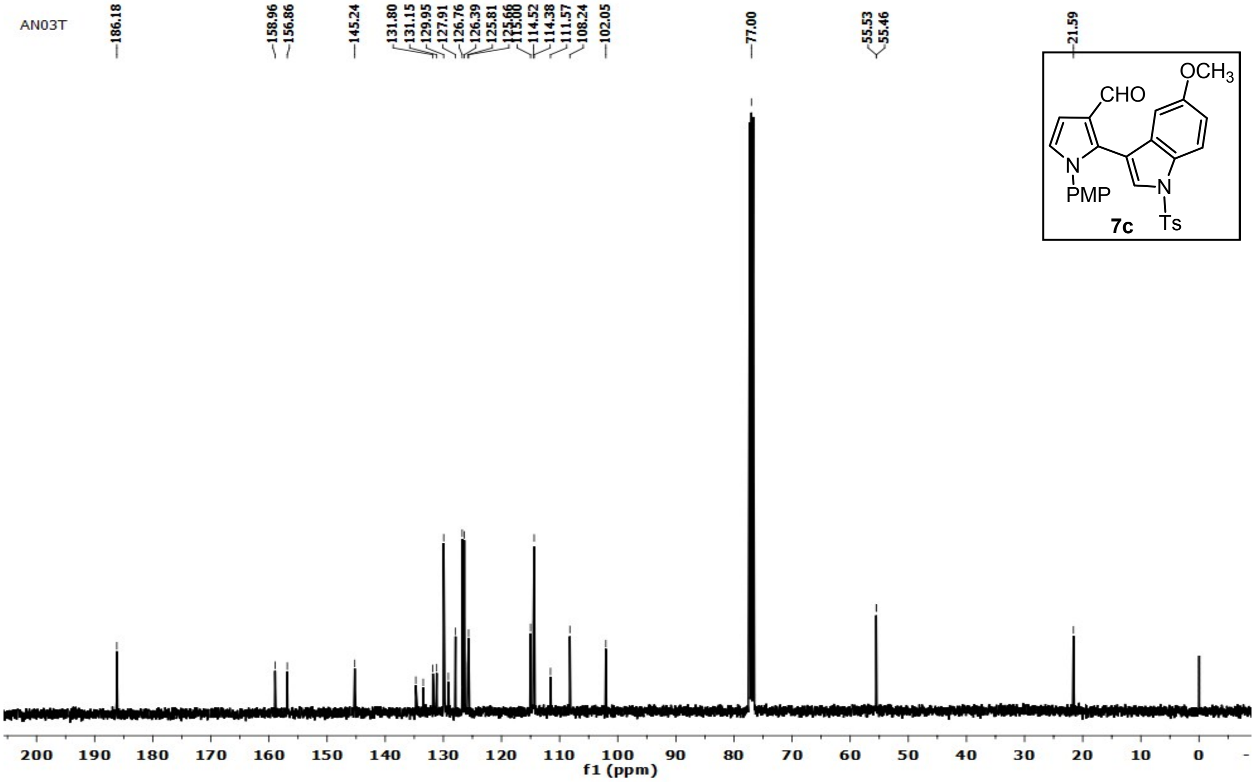
AN03T
186.18
158.96
156.86
145.24
131.80
131.15
129.95
127.91
126.76
126.39
125.81
125.66
115.00
114.52
114.38
111.57
108.24
102.05
77.00
55.53
55.46
21.59
f1 (ppm)
CHO
OCH_3
N
PMP
N
Ts
7c

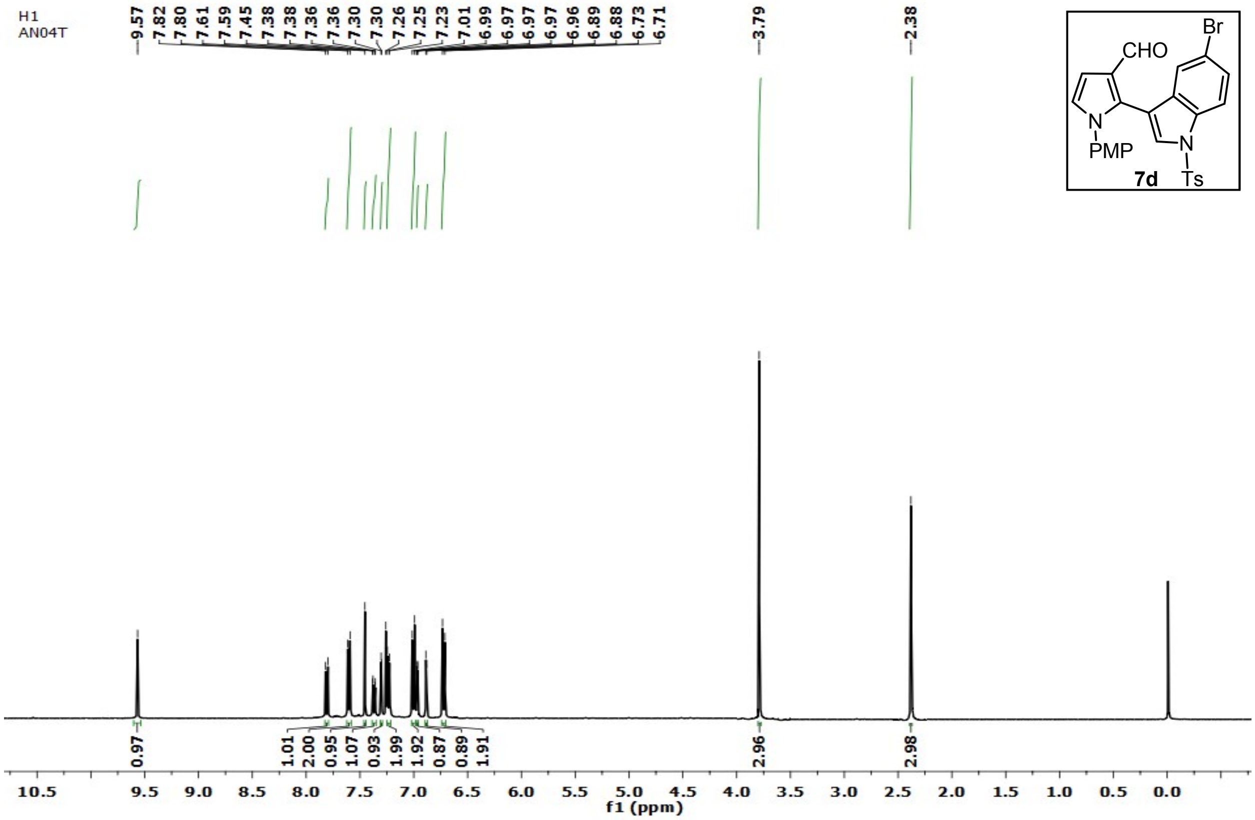
H1
AN04T
9.57
7.82
7.80
7.61
7.59
7.45
7.38
7.38
7.36
7.36
7.30
7.30
7.26
7.25
7.23
7.01
6.99
6.97
6.97
6.97
6.96
6.89
6.88
6.73
6.71
3.79
2.38
CHO
Br
N
PMP
N
7d
Ts
0.97
1.01
2.00
0.95
1.07
0.93
1.99
1.92
0.87
0.89
1.91
2.96
2.98
10.5
9.5
9.0
8.5
8.0
7.5
7.0
6.5
6.0
5.5
5.0
4.5
4.0
3.5
3.0
2.5
2.0
1.5
1.0
0.5
0.0
f1 (ppm)

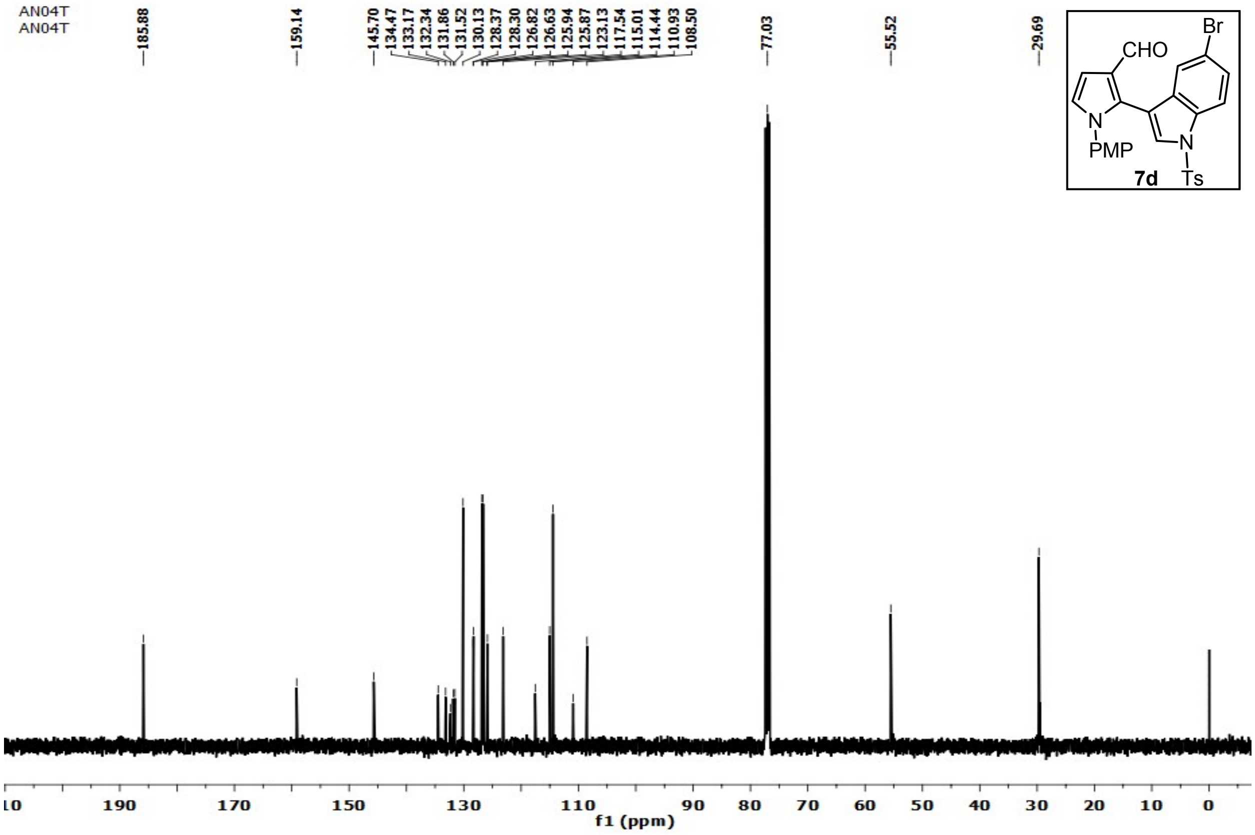
AN04T
AN04T
185.88
159.14
145.70
134.47
133.17
132.34
131.86
131.52
130.13
128.37
128.30
126.82
126.63
125.94
125.87
123.13
117.54
115.01
114.44
110.93
108.50
77.03
55.52
29.69
CHO
Br
N
PMP
N
7d
Ts
10
190
170
150
130
110
90
80
70
60
50
40
30
20
10
0
f1 (ppm)

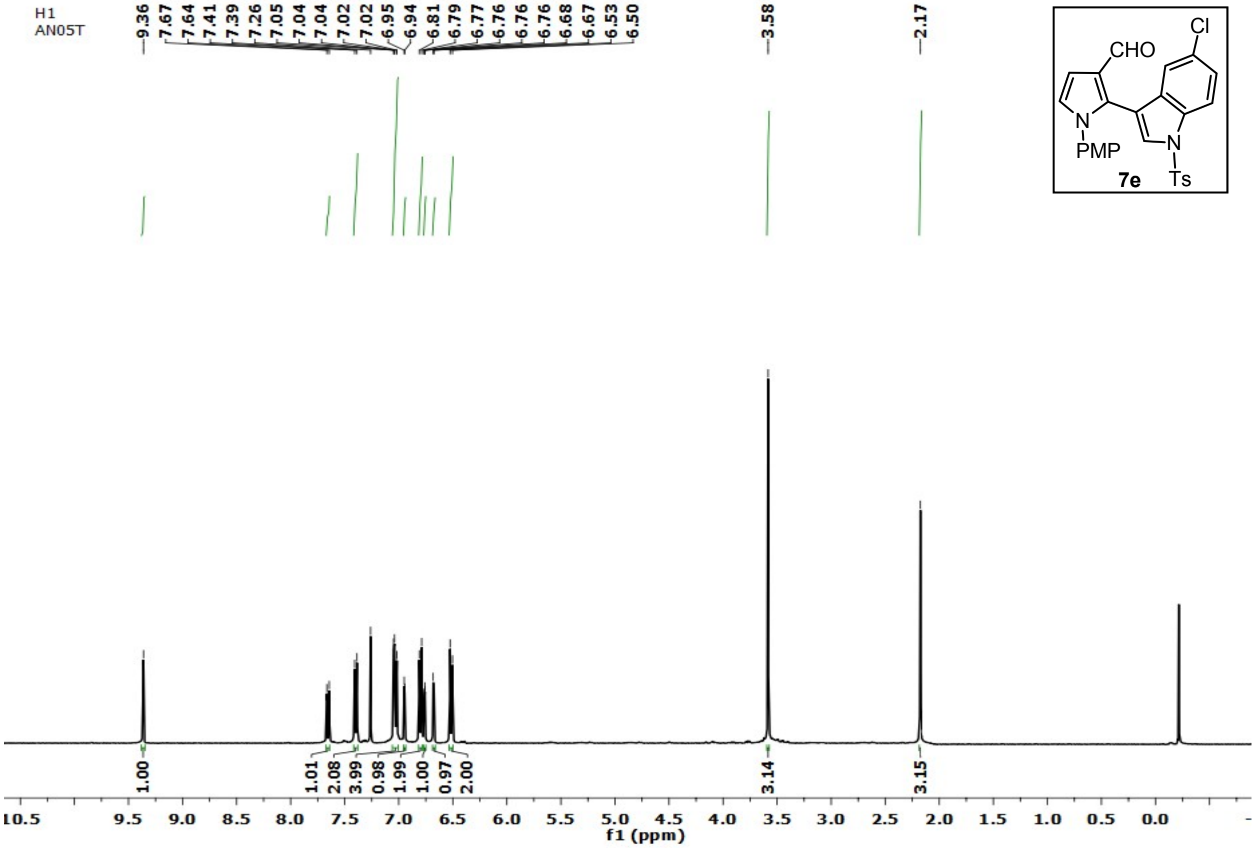
H1
AN05T
9.36
7.67
7.64
7.41
7.39
7.26
7.05
7.04
7.04
7.02
7.02
6.95
6.94
6.81
6.79
6.77
6.76
6.76
6.76
6.68
6.67
6.53
6.50
3.58
2.17
CHO
Cl
N
PMP
N
Ts
7e
1.00
1.01
2.08
3.99
0.98
1.99
1.00
0.97
2.00
3.14
3.15
f1 (ppm)

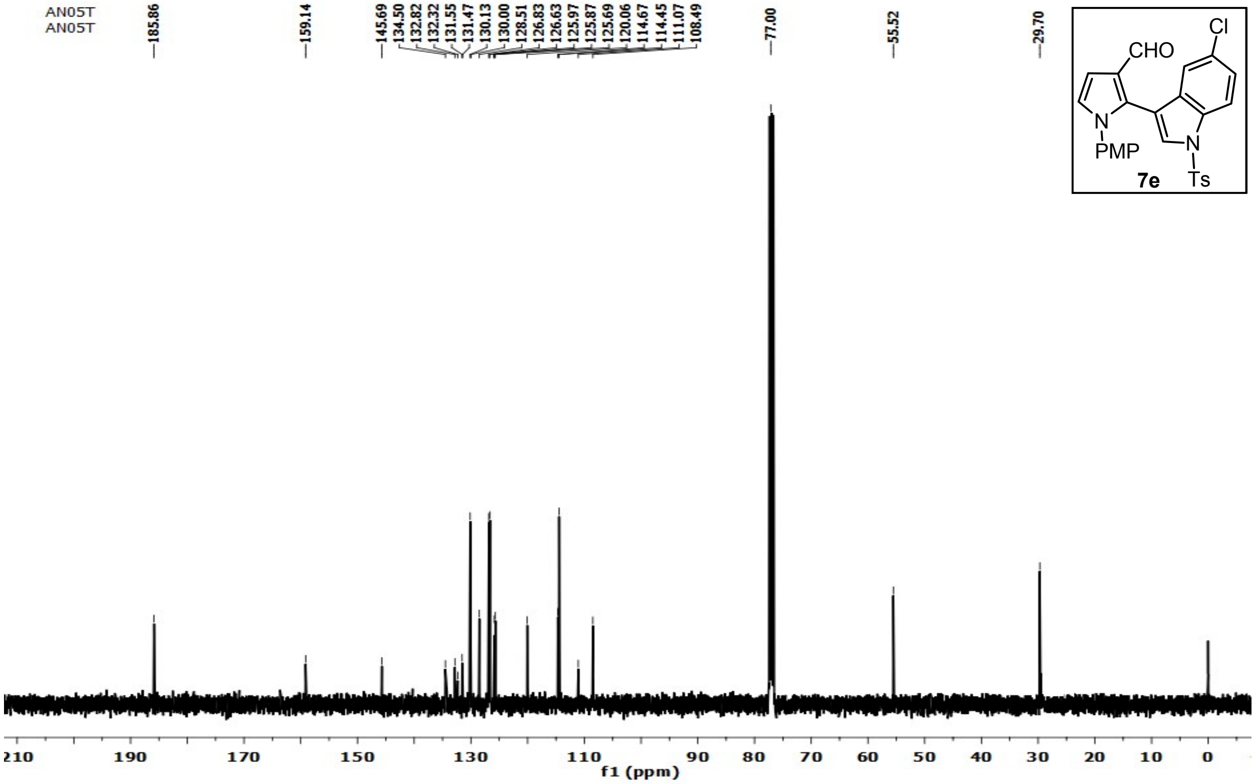
AN05T
AN05T
185.86
159.14
145.69
134.50
132.82
132.32
131.55
131.47
130.13
130.00
128.51
126.83
126.63
125.97
125.87
125.69
120.06
114.67
114.45
111.07
108.49
77.00
55.52
29.70
CHO
Cl
N
PMP
N
Ts
7e
f1 (ppm)

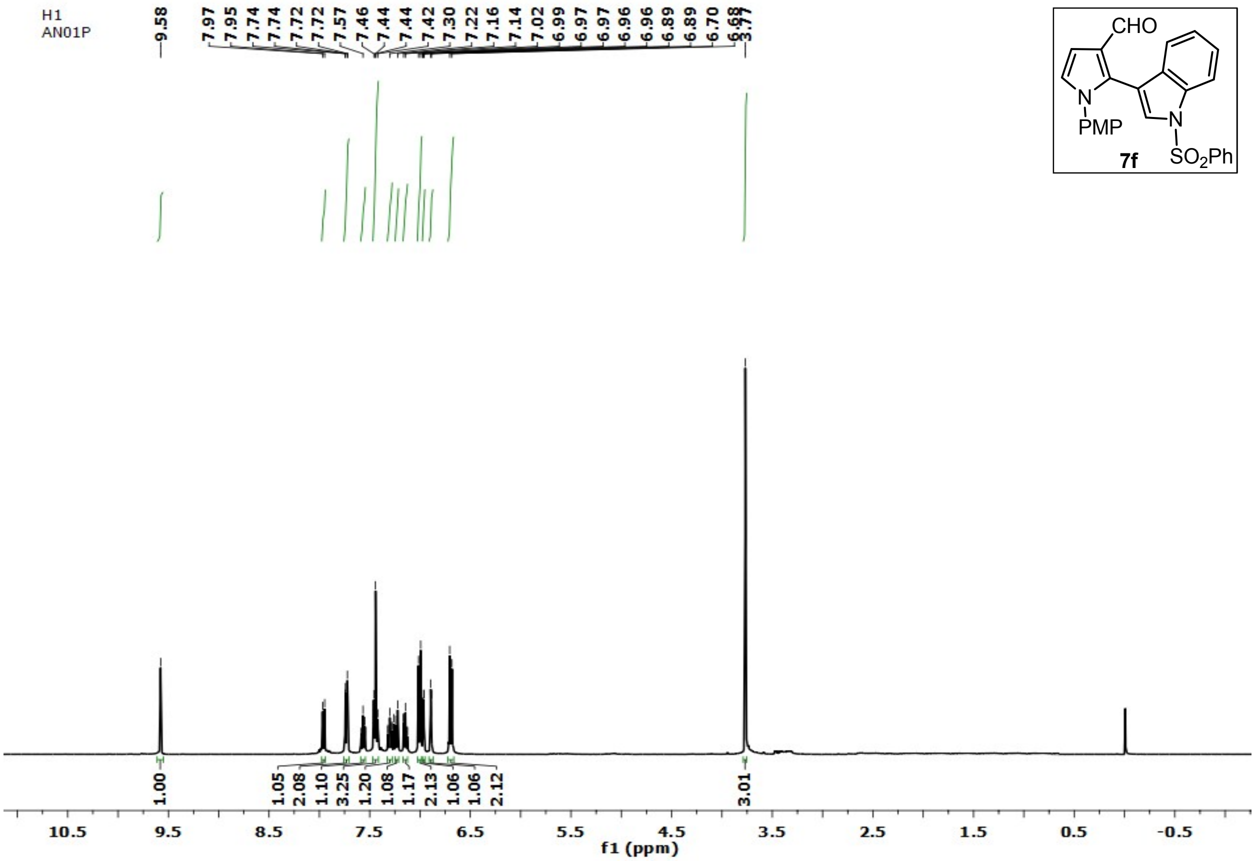
H1
AN01P
9.58
7.97
7.95
7.74
7.74
7.72
7.72
7.57
7.46
7.44
7.44
7.42
7.30
7.22
7.16
7.14
7.02
6.99
6.97
6.97
6.96
6.96
6.89
6.89
6.70
6.68
3.77
CHO
N
PMP
N
SO2Ph
7f
1.00
1.05
2.08
1.10
3.25
1.20
1.08
1.17
2.13
1.06
1.06
2.12
3.01
10.5
9.5
8.5
7.5
6.5
5.5
4.5
3.5
2.5
1.5
0.5
-0.5
f1 (ppm)

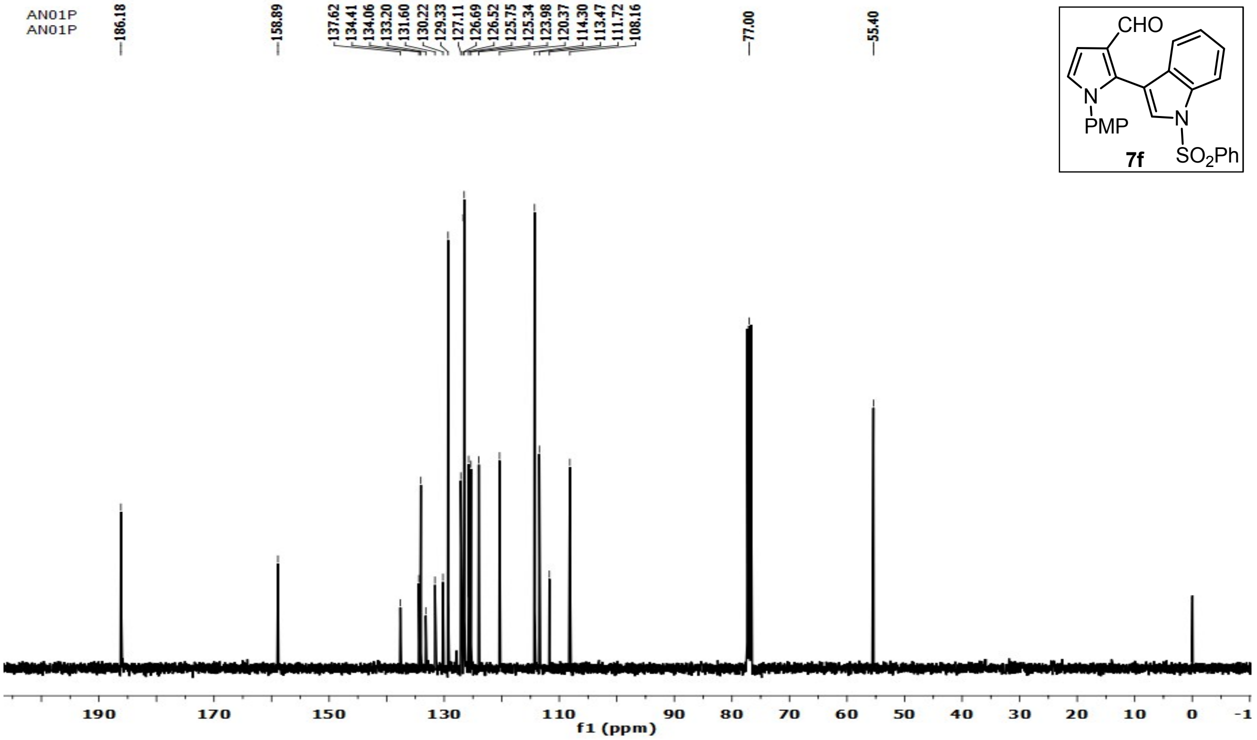
AN01P
AN01P
186.18
158.89
137.62
134.41
134.06
133.20
131.60
130.22
129.33
127.11
126.69
126.52
125.75
125.34
123.98
120.37
114.30
113.47
111.72
108.16
77.00
55.40
CHO
N
PMP
N
SO2Ph
7f
190
170
150
130
110
90
80
70
60
50
40
30
20
10
0
-1
f1 (ppm)

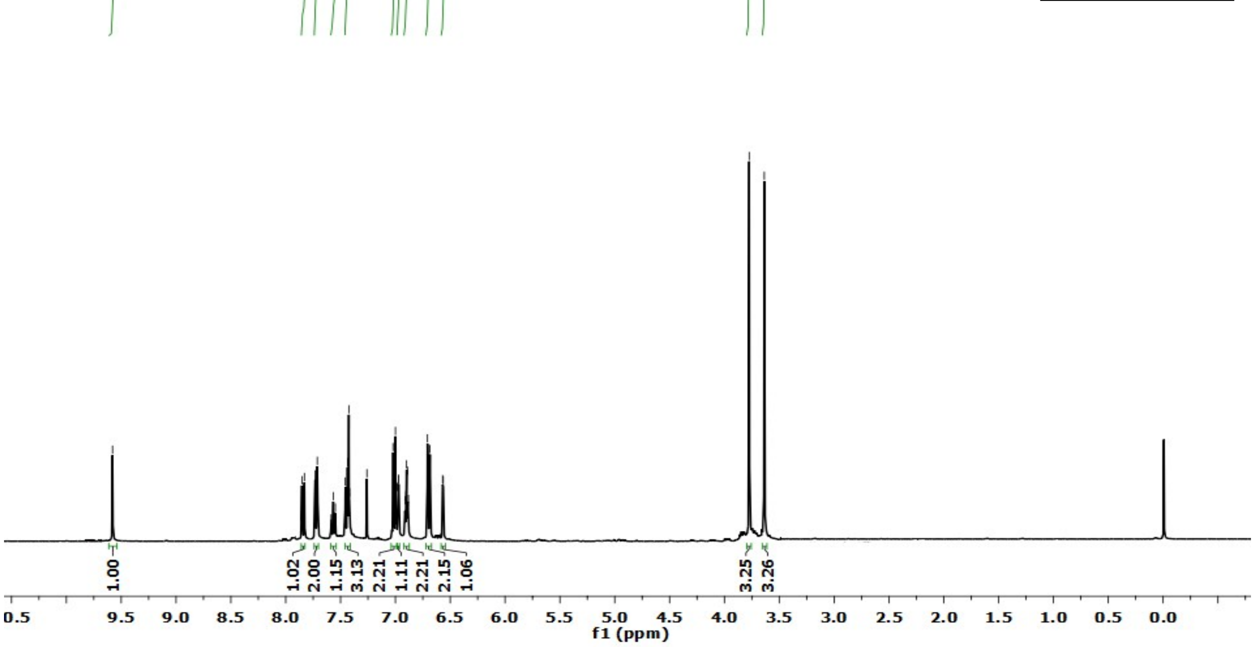
H1
AN02P
9.58
7.85
7.83
7.73
7.73
7.71
7.71
7.57
7.46
7.45
7.44
7.42
7.42
7.26
7.02
7.00
6.98
6.98
6.97
6.97
6.91
6.90
6.90
6.89
6.88
6.71
6.69
6.57
3.64
1.00
1.02
2.00
1.15
3.13
2.21
1.11
2.21
2.15
1.06
3.25
3.26
f1 (ppm)

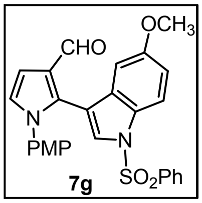
OCH3
CHO
N
PMP
N
SO2Ph
7g

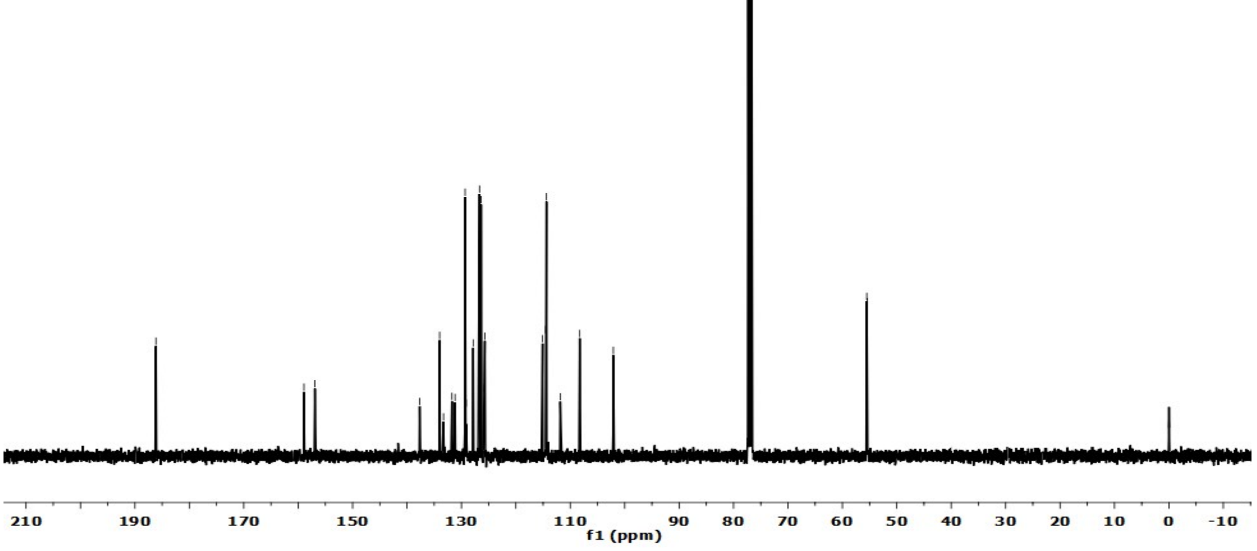
ANO2P
AN02P
186.14
158.94
156.92
137.67
134.01
131.75
131.17
129.33
129.12
127.84
126.68
126.39
125.80
114.52
114.38
111.84
108.26
102.08
77.00
55.52
55.46
f1 (ppm)

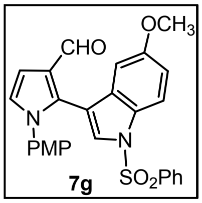
OCH3
CHO
N
PMP
N
SO2Ph
7g

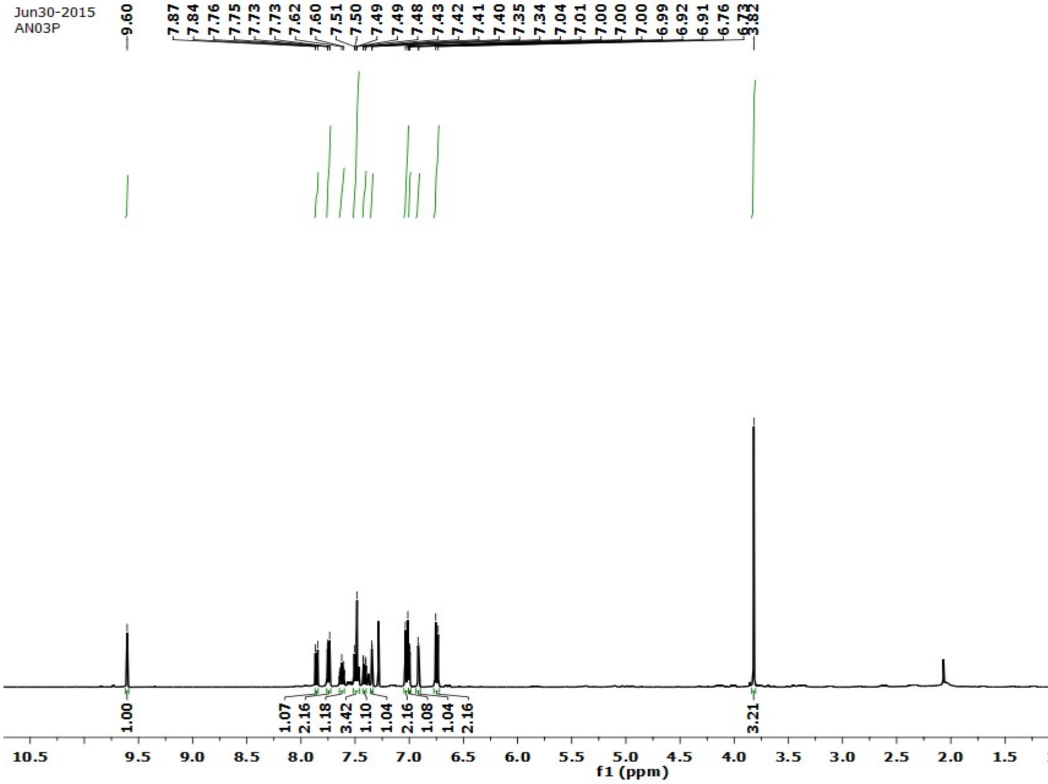
Jun30-2015
AN03P
9.60
7.87 7.84 7.76 7.75 7.73 7.73 7.62 7.60 7.51 7.50 7.49 7.49 7.48 7.43 7.42 7.41 7.40 7.35 7.34 7.04 7.01 7.00 7.00 7.00 6.99 6.92 6.91 6.76 6.73
3.82
1.00
1.07 2.16 1.18 3.42 1.10 1.04 2.16 1.08 1.04 2.16
3.21
10.5 9.5 9.0 8.5 8.0 7.5 7.0 6.5 6.0 5.5 5.0 4.5 4.0 3.5 3.0 2.5 2.0 1.5 1.0 0.5 0.0 -0.5
f1 (ppm)

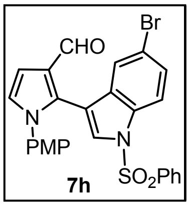
CHO
Br
N
PMP
N
SO2Ph

7h

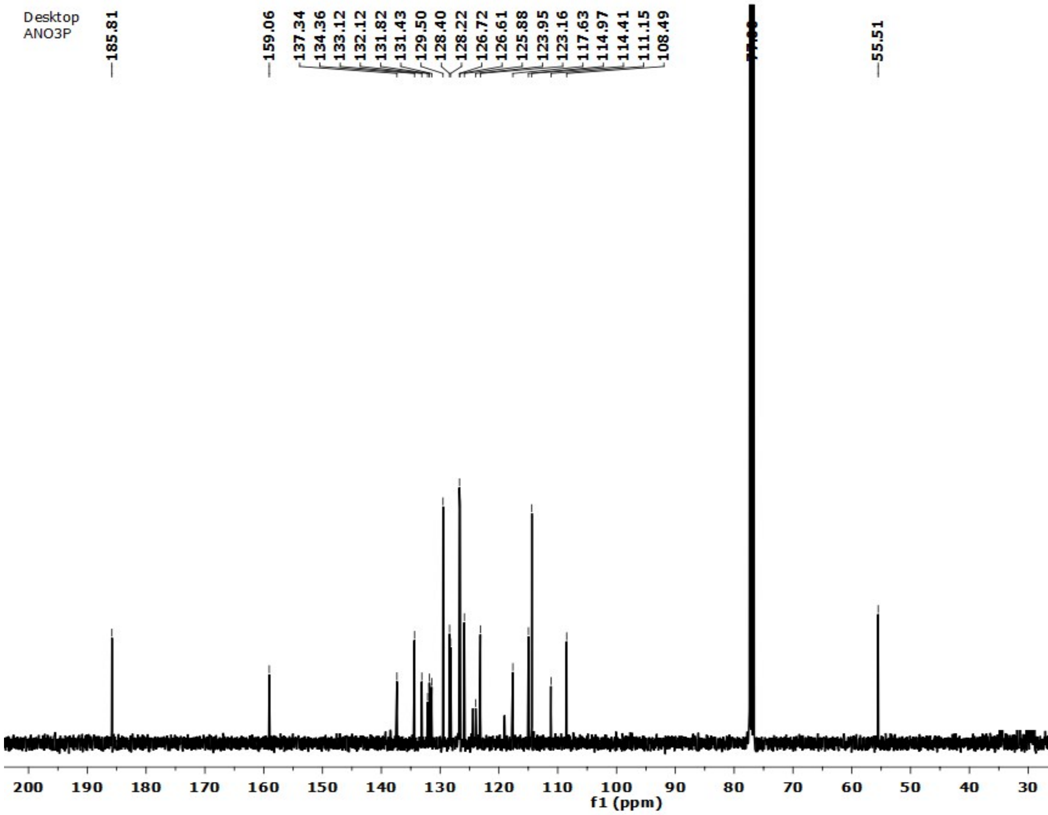
Desktop
ANO3P
185.81
159.06
137.34 134.36 133.12 132.12 131.82 131.43 129.50 128.40 128.22 126.72 126.61 125.88 123.95 123.16 117.63 114.97 114.41 111.15 108.49
55.51
200 190 180 170 160 150 140 130 120 110 100 90 80 70 60 50 40 30 20 10 0
f1 (ppm)

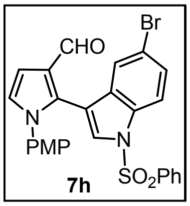
CHO
Br
N
PMP
N
SO2Ph

7h

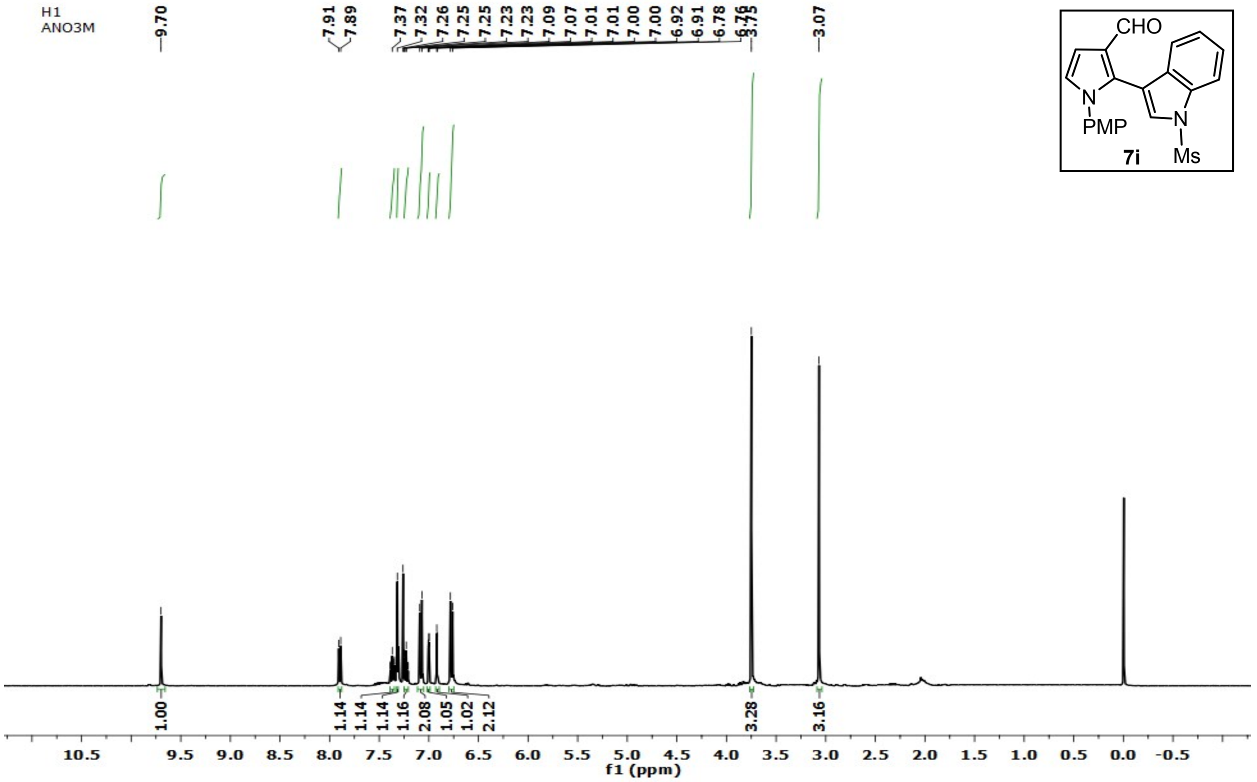
H1
ANO3M
9.70
7.91
7.89
7.37
7.32
7.26
7.25
7.25
7.23
7.23
7.09
7.07
7.01
7.01
7.00
7.00
6.92
6.91
6.78
6.76
3.75
3.07
1.00
1.14
1.14
1.14
1.16
2.08
1.05
1.02
2.12
3.28
3.16
f1 (ppm)
CHO
N
PMP
N
Ms
7i

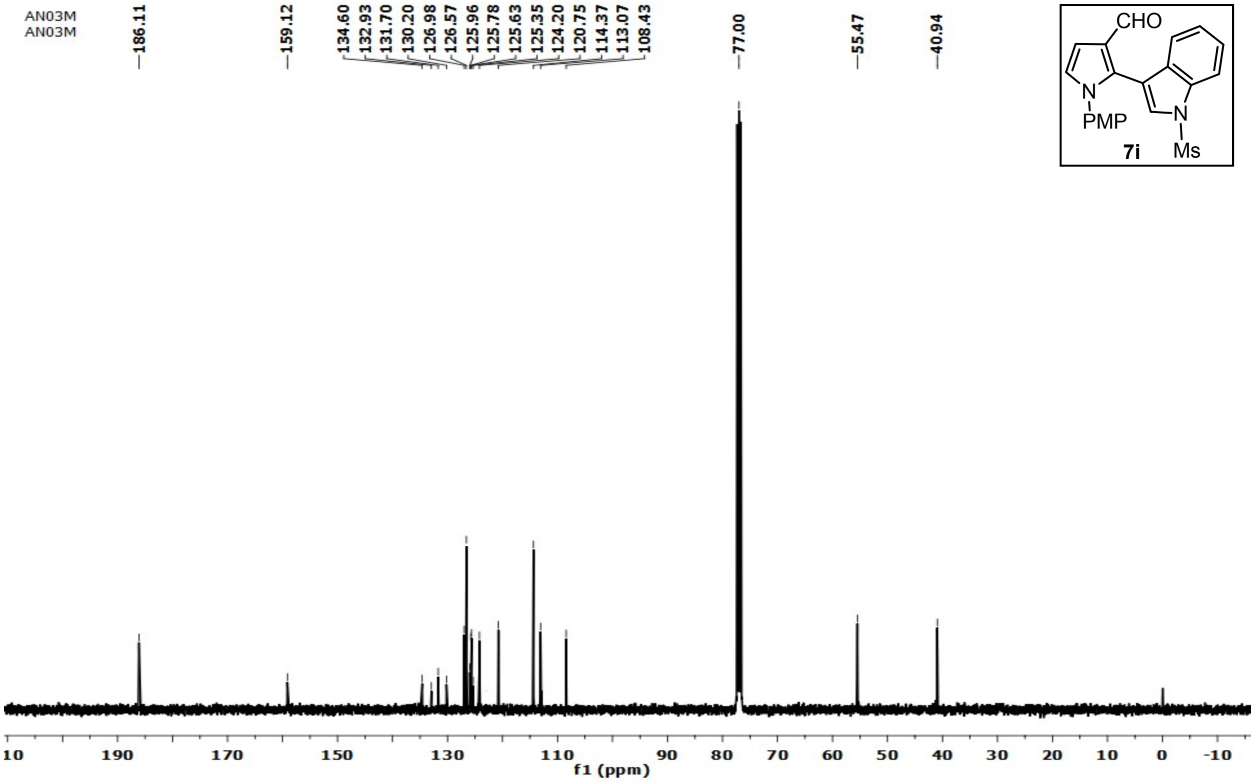
AN03M
AN03M
186.11
159.12
134.60
132.93
131.70
130.20
126.98
126.57
125.96
125.78
125.63
125.35
124.20
120.75
114.37
113.07
108.43
77.00
55.47
40.94
f1 (ppm)
CHO
N
PMP
N
Ms
7i

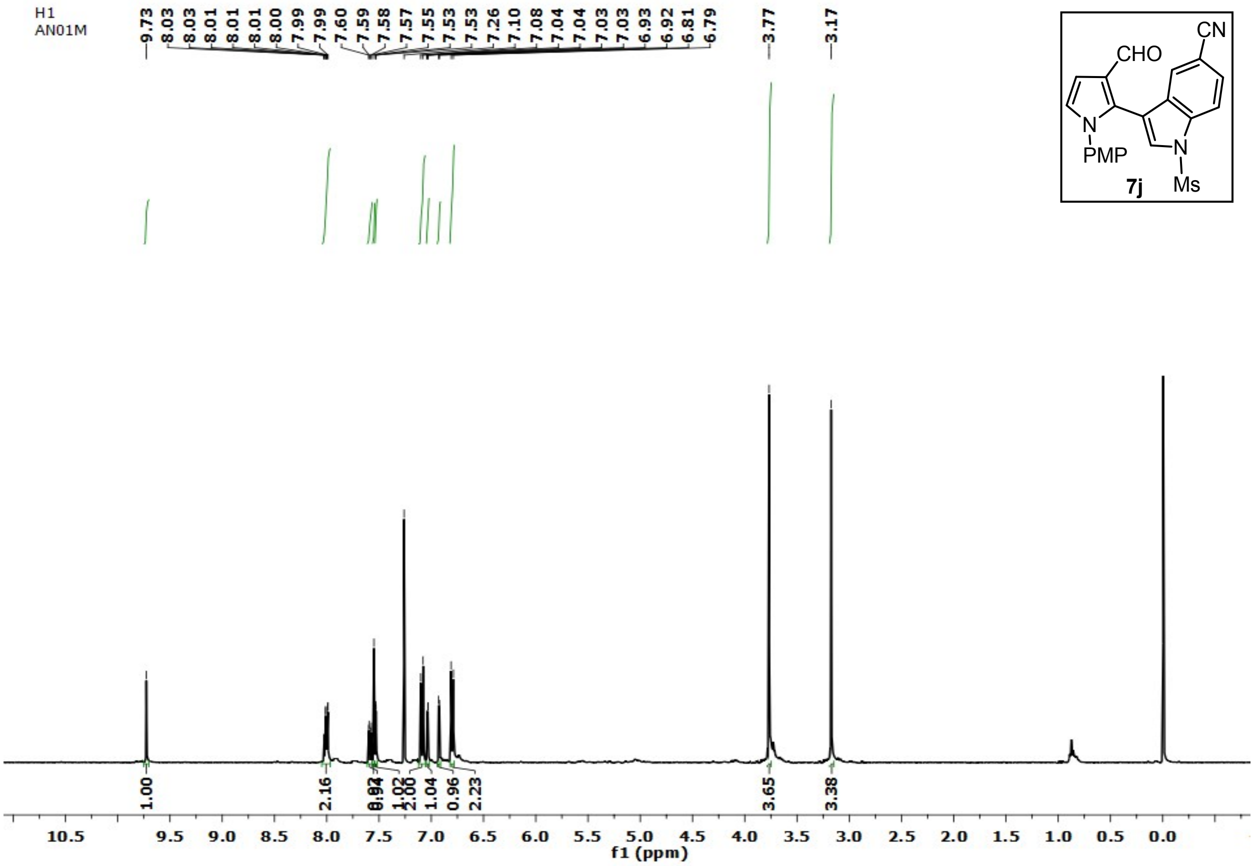
H1
AN01M
9.73 8.03 8.03 8.01 8.01 8.00 7.99 7.99 7.60 7.59 7.58 7.57 7.55 7.53 7.53 7.26 7.10 7.08 7.04 7.04 7.03 7.03 6.93 6.92 6.81 6.79
3.77
3.17
CHO
CN
N
PMP
N
Ms
7j
1.00 2.16 0.92 0.94 1.02 2.00 1.04 0.96 2.23 3.65 3.38
f1 (ppm)

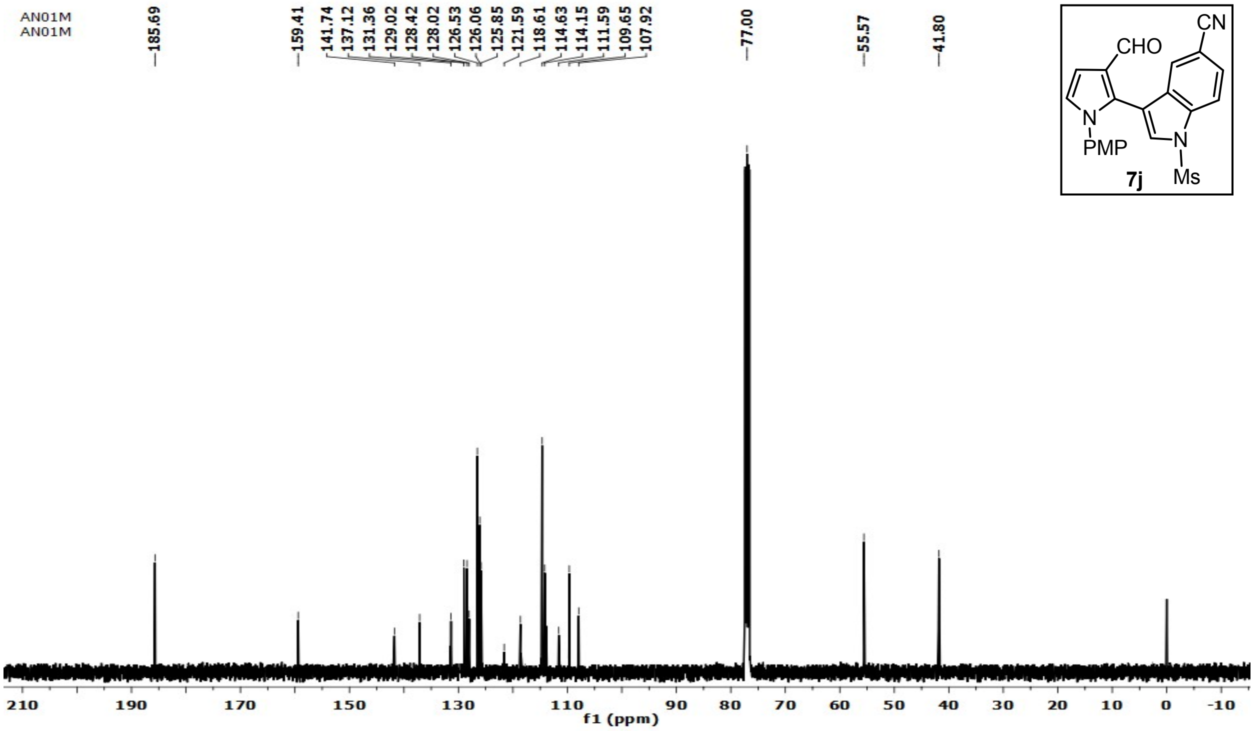
AN01M
AN01M
185.69
159.41 141.74 137.12 131.36 129.02 128.42 128.02 126.53 126.06 125.85 121.59 118.61 114.63 114.15 111.59 109.65 107.92
77.00
55.57
41.80
CHO
CN
N
PMP
N
Ms
7j
f1 (ppm)

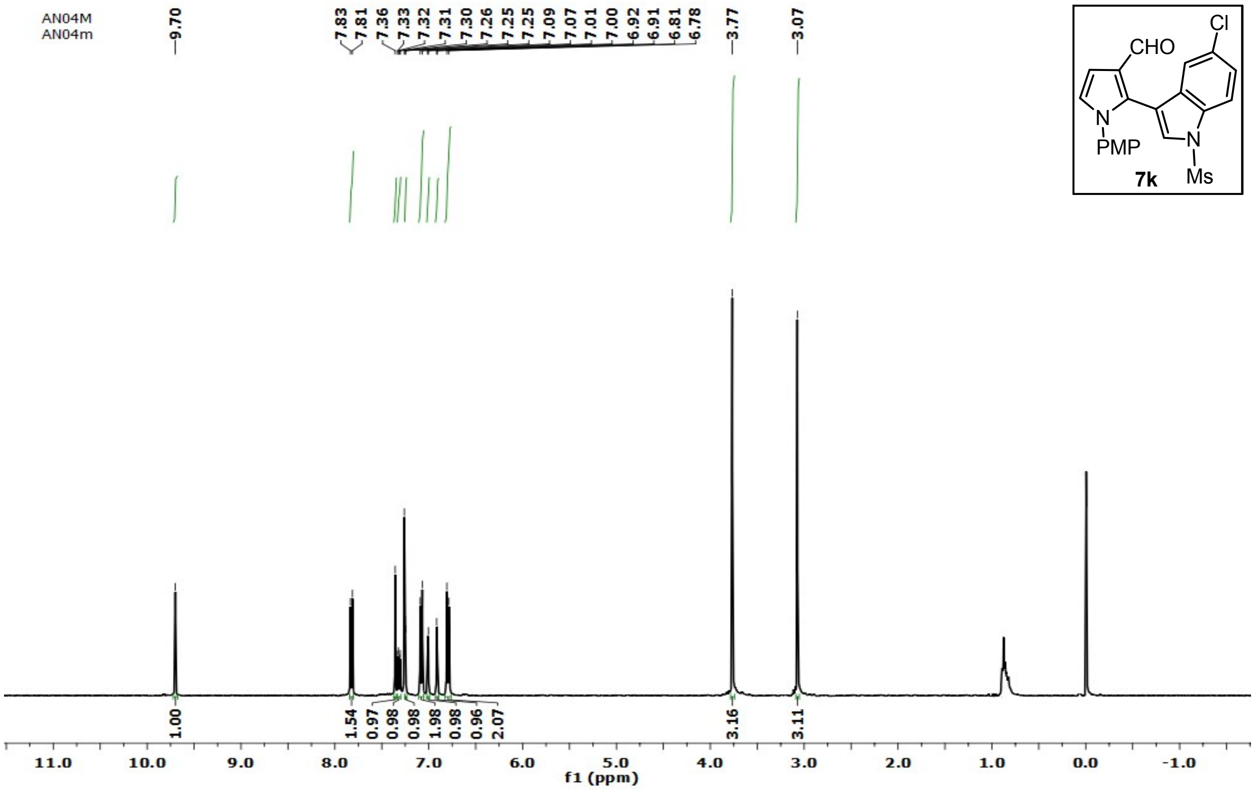
AN04M
AN04m
9.70
7.83
7.81
7.36
7.33
7.32
7.31
7.30
7.26
7.25
7.25
7.09
7.07
7.01
7.00
6.92
6.91
6.81
6.78
3.77
3.07
1.00
1.54
0.97
0.98
0.98
1.98
0.98
0.96
2.07
3.16
3.11
11.0
10.0
9.0
8.0
7.0
6.0
5.0
4.0
3.0
2.0
1.0
0.0
-1.0
f1 (ppm)
CHO
Cl
N
PMP
N
Ms
7k

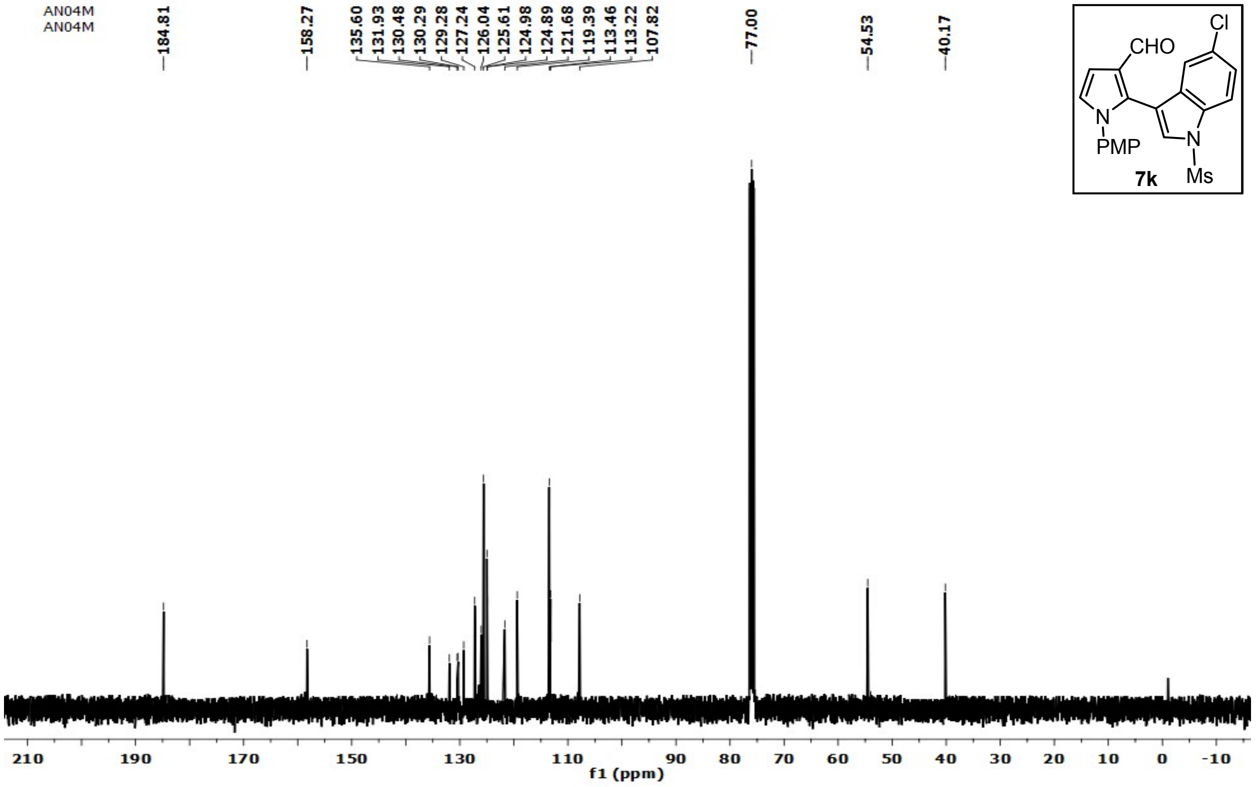
AN04M
AN04M
184.81
158.27
135.60
131.93
130.48
130.29
129.28
127.24
126.04
125.61
124.98
124.89
121.68
119.39
113.46
113.22
107.82
77.00
54.53
40.17
210
190
170
150
130
110
90
80
70
60
50
40
30
20
10
0
-10
f1 (ppm)
CHO
Cl
N
PMP
N
Ms
7k

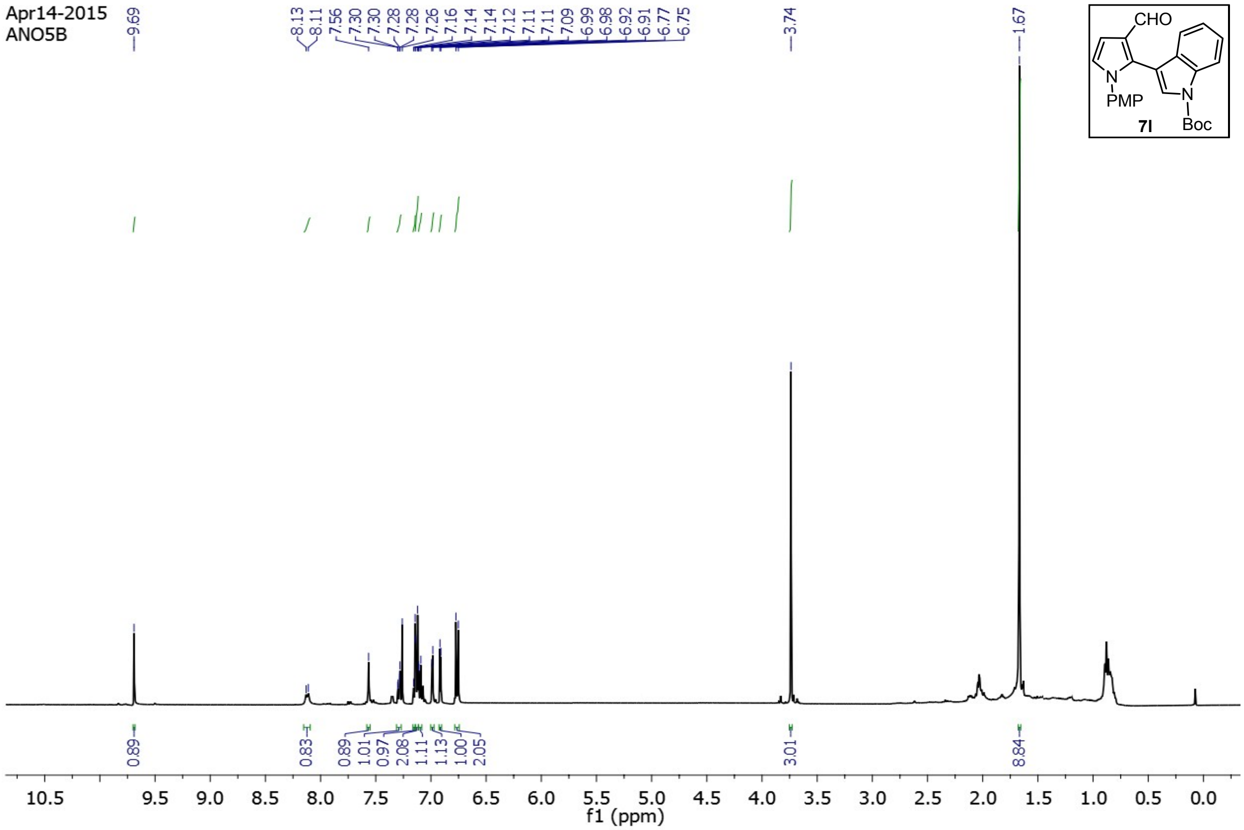

Apr14-2015
ANO5B
9.69
8.13
8.11
7.56
7.30
7.30
7.28
7.28
7.26
7.16
7.14
7.14
7.12
7.11
7.11
7.09
6.99
6.98
6.92
6.91
6.77
6.75
3.74
1.67
CHO
N
PMP
N
Boc
7l
0.89
0.83
0.89
1.01
0.97
2.08
1.11
1.13
1.00
2.05
3.01
8.84
10.5
9.5
9.0
8.5
8.0
7.5
7.0
6.5
6.0
5.5
5.0
4.5
4.0
3.5
3.0
2.5
2.0
1.5
1.0
0.5
0.0
f1 (ppm)

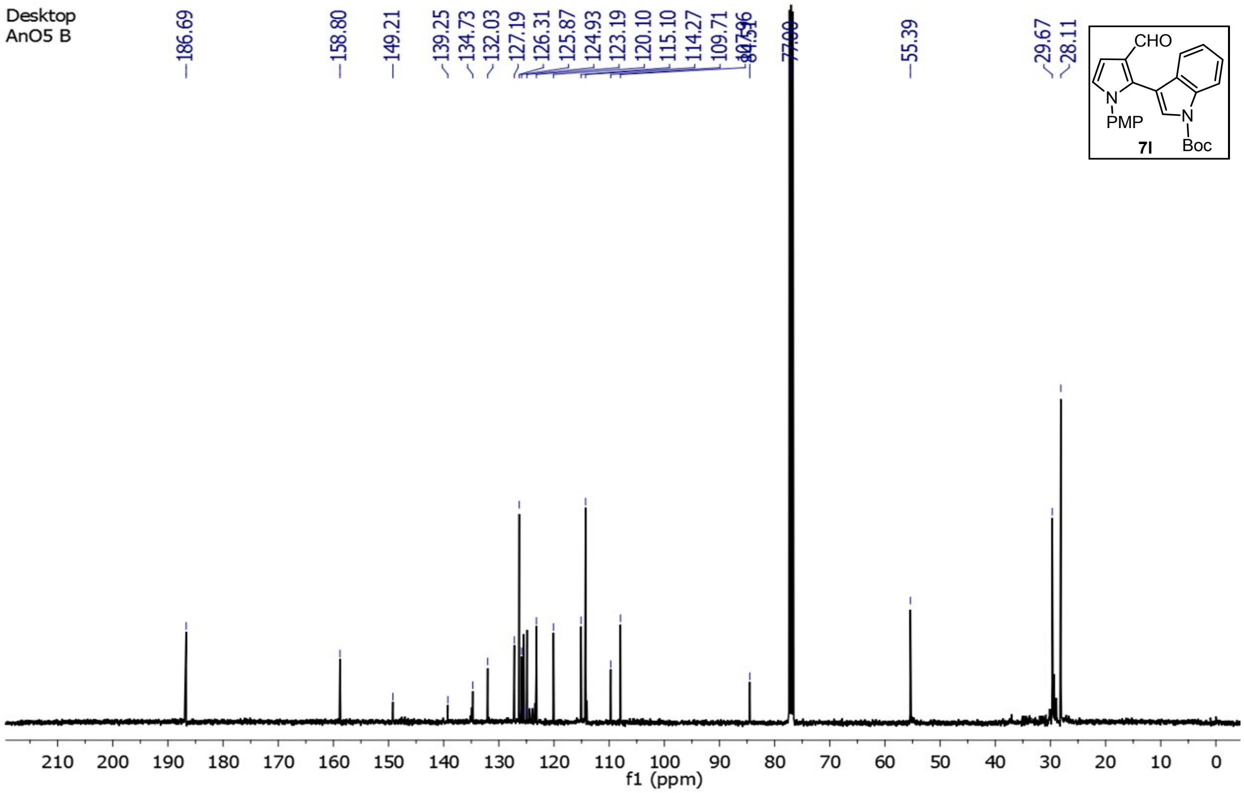

Desktop
AnO5 B
186.69
158.80
149.21
139.25
134.73
132.03
127.19
126.31
125.87
124.93
123.19
120.10
115.10
114.27
109.71
84.51
77.00
55.39
29.67
28.11
CHO
N
PMP
N
Boc
7l
210
200
190
180
170
160
150
140
130
120
110
100
90
80
70
60
50
40
30
20
10
0
f1 (ppm)

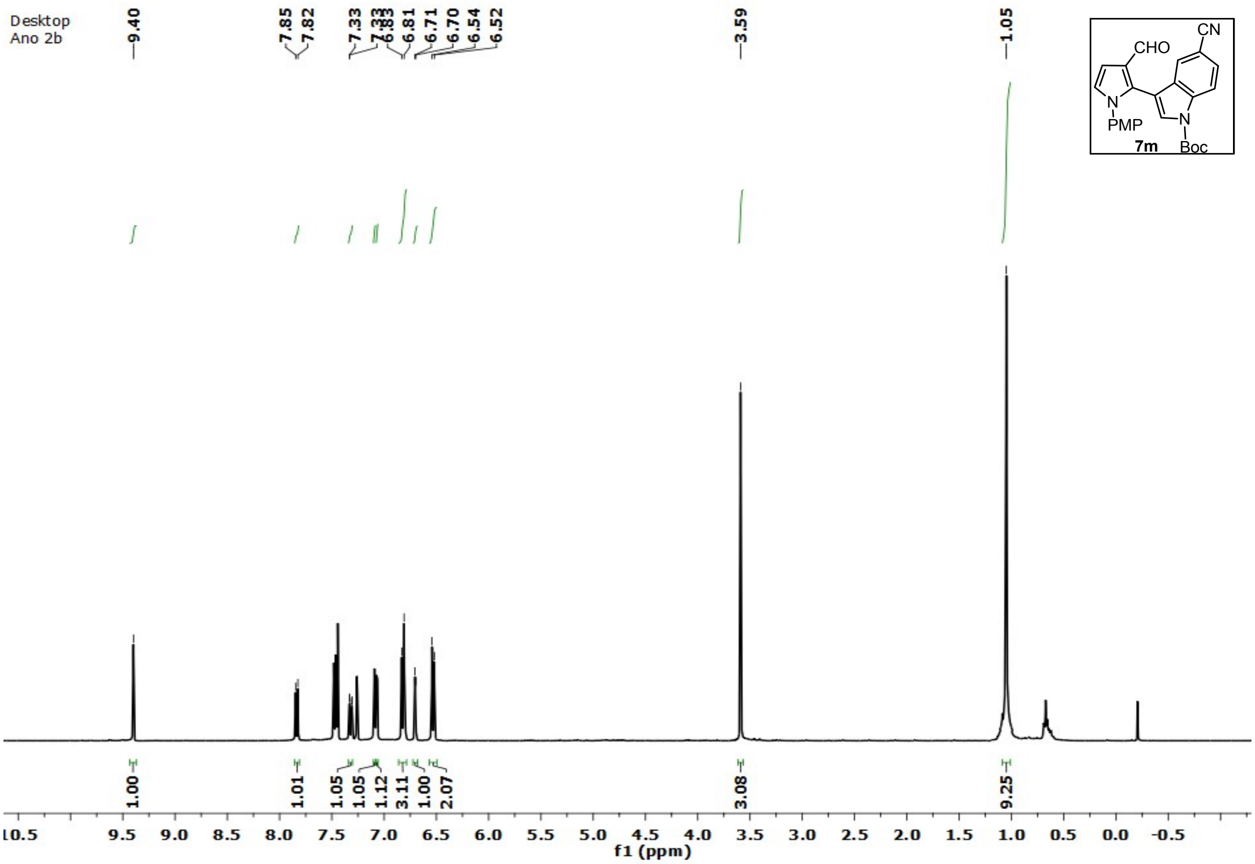
Desktop
Ano 2b
9.40
7.85
7.82
7.33
7.33
6.83
6.81
6.71
6.70
6.54
6.52
3.59
1.05
CHO
CN
N
PMP
7m
Boc
1.00
1.01
1.05
1.05
1.12
3.11
1.00
2.07
3.08
9.25
f1 (ppm)

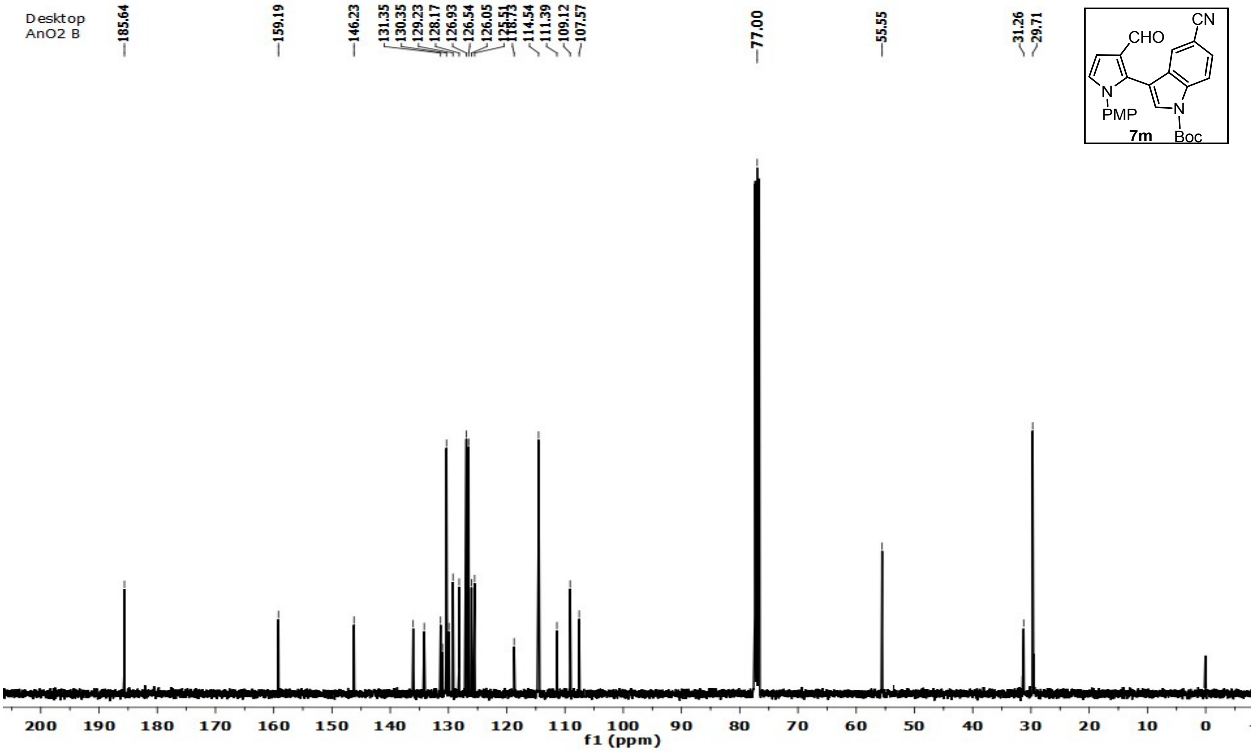
Desktop
AnO2 B
185.64
159.19
146.23
131.35
130.35
129.23
128.17
126.93
126.54
126.05
114.54
111.39
109.12
107.57
77.00
55.55
31.26
29.71
CHO
CN
N
PMP
7m
Boc
f1 (ppm)

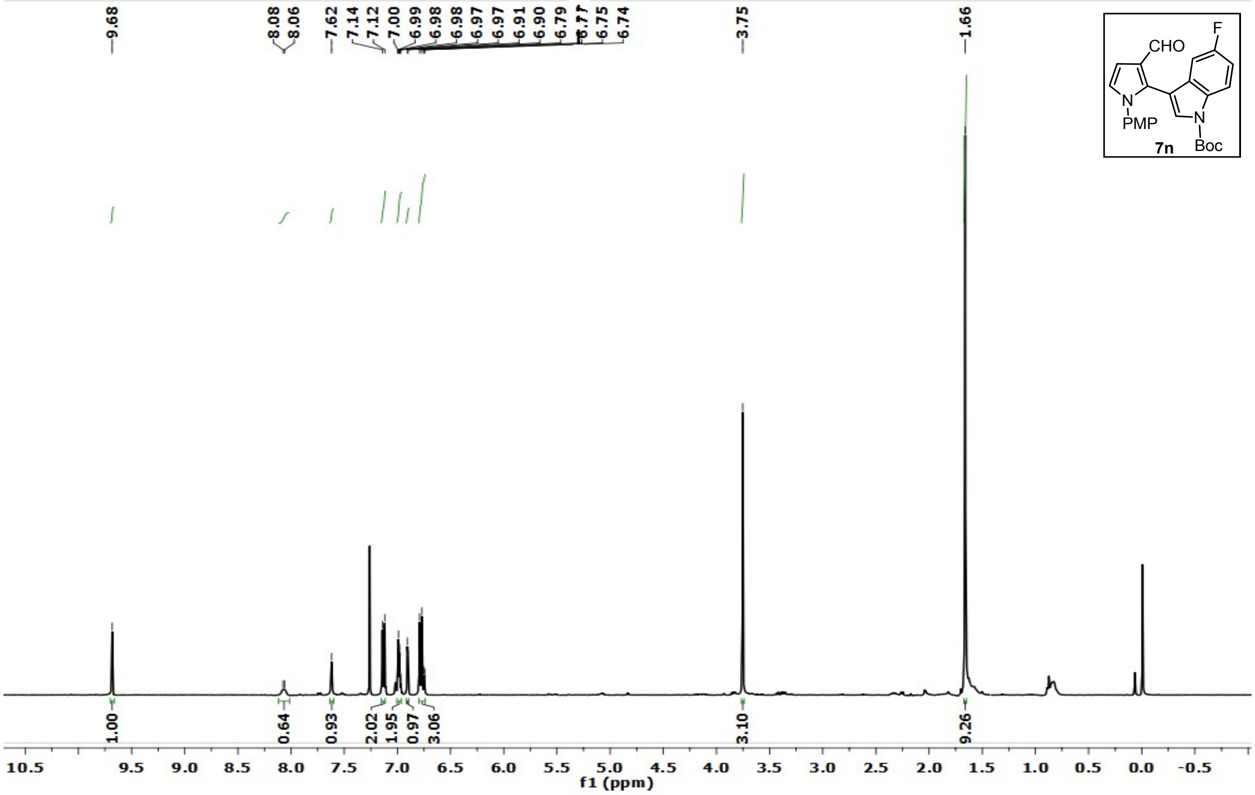
9.68
8.08
8.06
7.62
7.14
7.12
7.00
6.99
6.98
6.98
6.97
6.97
6.91
6.90
6.79
6.75
6.74
3.75
1.66
CHO
F
N
PMP
N
7n
Boc
1.00
0.64
0.93
2.02
1.95
0.97
3.06
3.10
9.26
f1 (ppm)

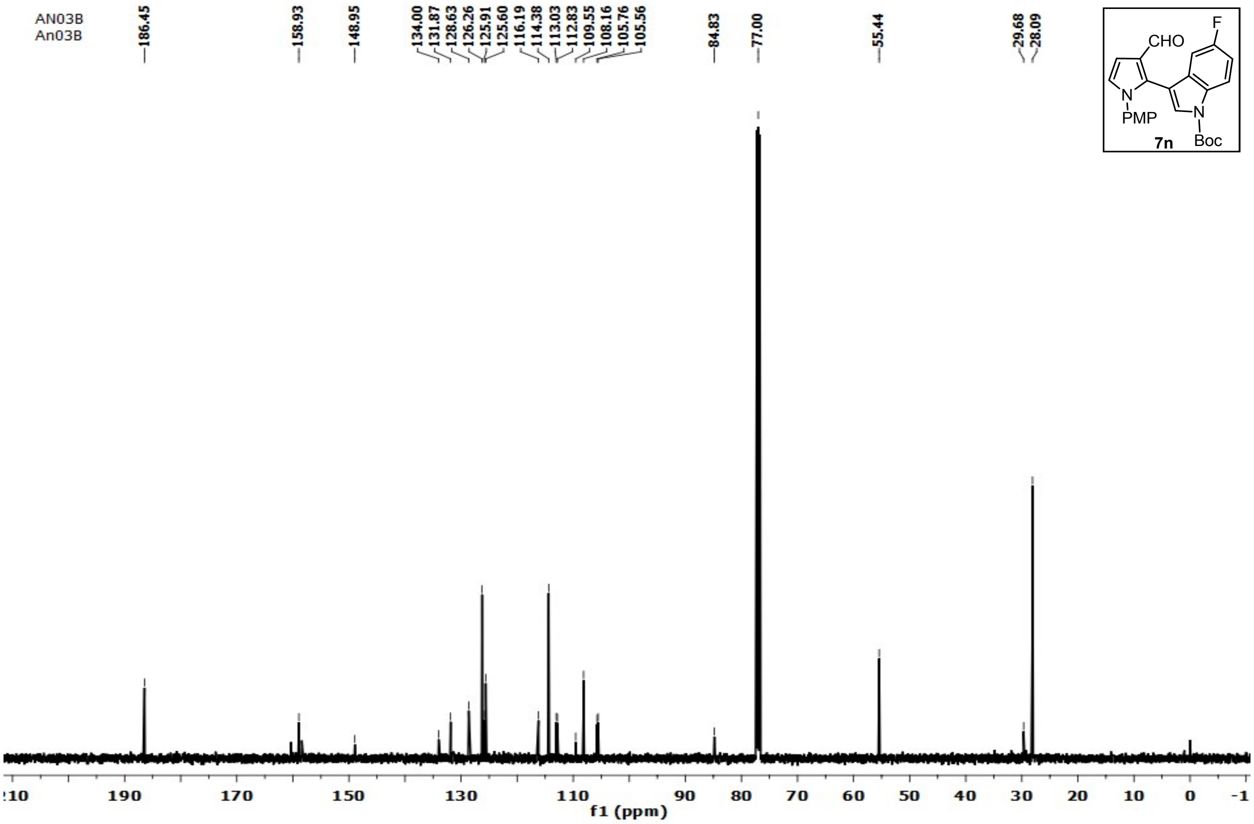
AN03B
An03B
186.45
158.93
148.95
134.00
131.87
128.63
126.26
125.91
125.60
116.19
114.38
113.03
112.83
109.55
108.16
105.76
105.56
84.83
77.00
55.44
29.68
28.09
CHO
F
N
PMP
N
7n
Boc
f1 (ppm)

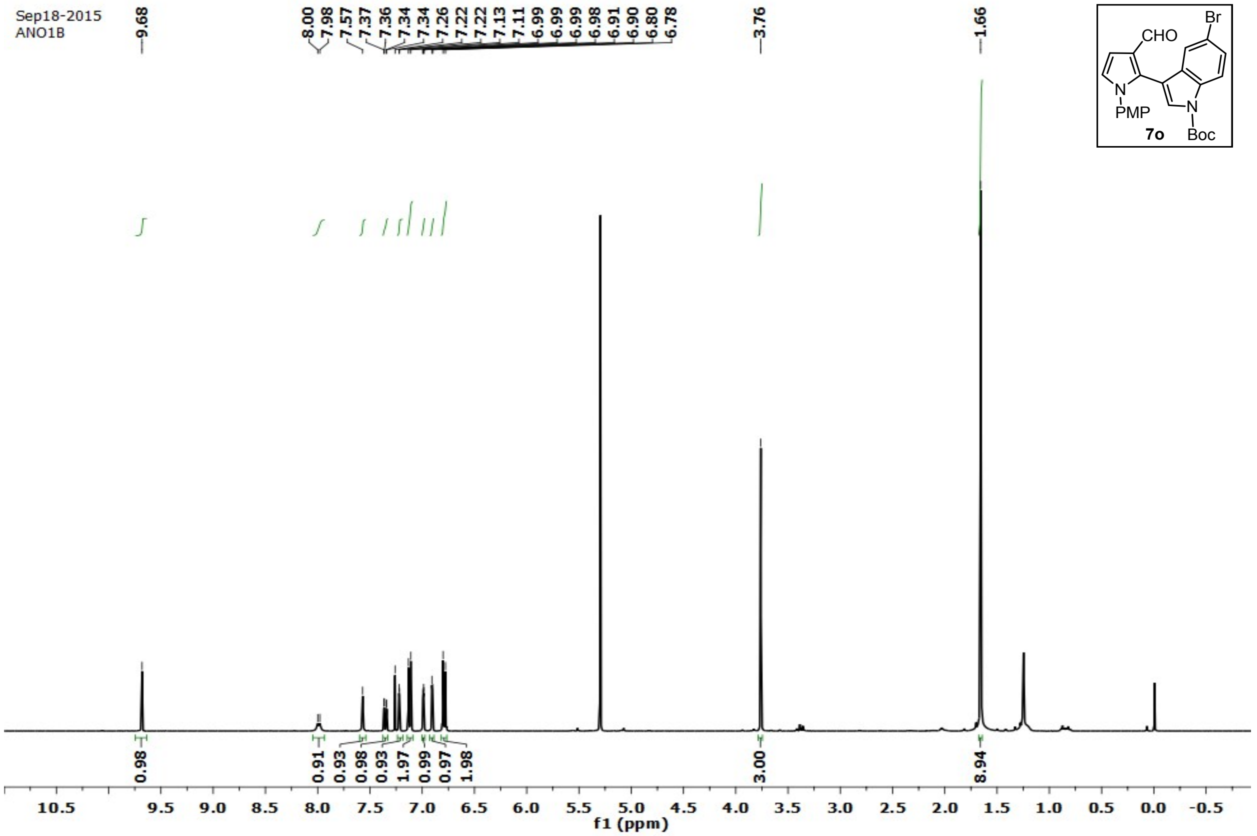
Sep18-2015
ANO1B
9.68
8.00
7.98
7.57
7.37
7.36
7.34
7.34
7.26
7.22
7.22
7.13
7.11
6.99
6.99
6.98
6.91
6.90
6.80
6.78
3.76
1.66
CHO
Br
N
PMP
N
Boc
7o
0.98
0.91
0.93
0.98
0.93
1.97
0.99
0.97
1.98
3.00
8.94
f1 (ppm)

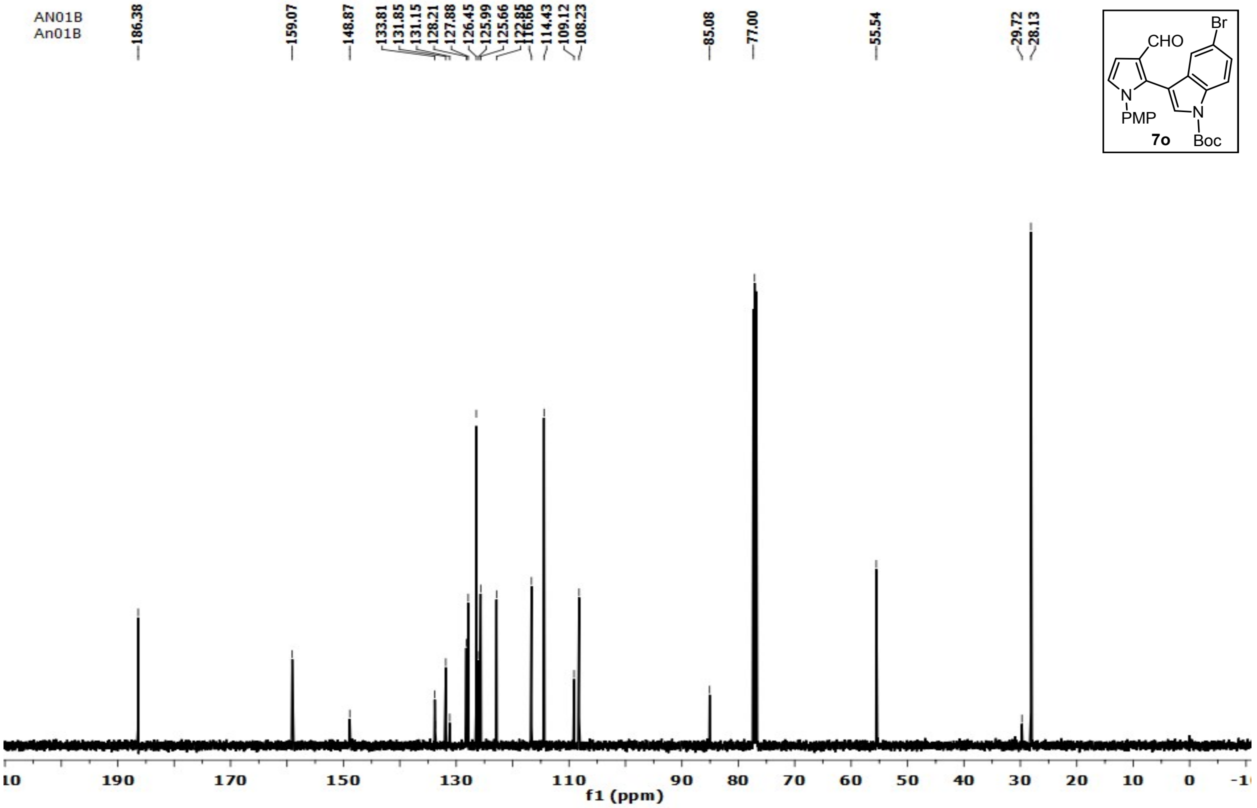
AN01B
An01B
186.38
159.07
148.87
133.81
131.85
131.15
128.21
127.88
126.45
125.99
125.66
109.12
108.23
114.43
85.08
77.00
55.54
29.72
28.13
CHO
Br
N
PMP
N
Boc
7o
f1 (ppm)

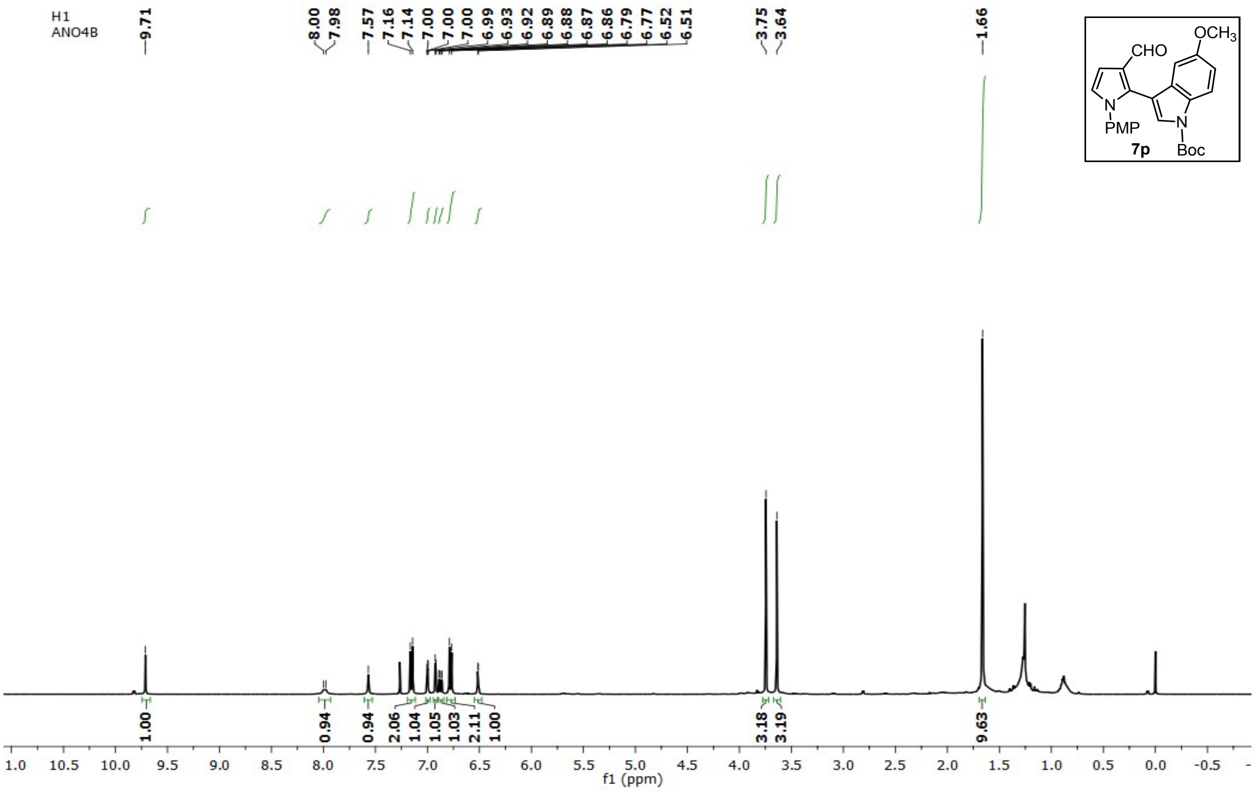
H1
ANO4B
9.71
8.00
7.98
7.57
7.16
7.14
7.00
7.00
7.00
6.99
6.93
6.92
6.89
6.88
6.87
6.86
6.79
6.77
6.52
6.51
3.75
3.64
1.66
CHO
OCH3
N
PMP
N
7p
Boc
1.00
0.94
0.94
2.06
1.04
1.05
1.03
2.11
1.00
3.18
3.19
9.63
f1 (ppm)

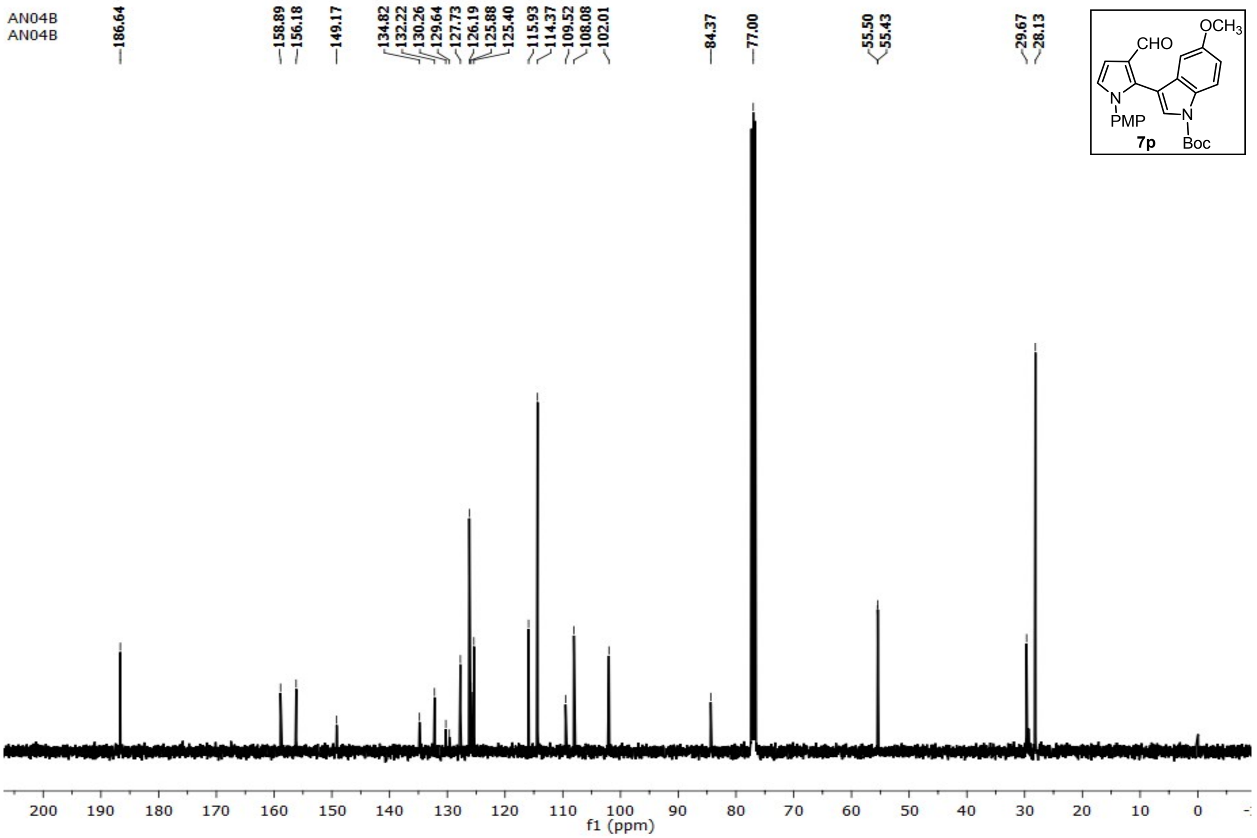
AN04B
AN04B
186.64
158.89
156.18
149.17
134.82
132.22
130.26
129.64
127.73
126.19
125.88
125.40
115.93
114.37
109.52
108.08
102.01
84.37
77.00
55.50
55.43
29.67
28.13
CHO
OCH3
N
PMP
N
7p
Boc
f1 (ppm)

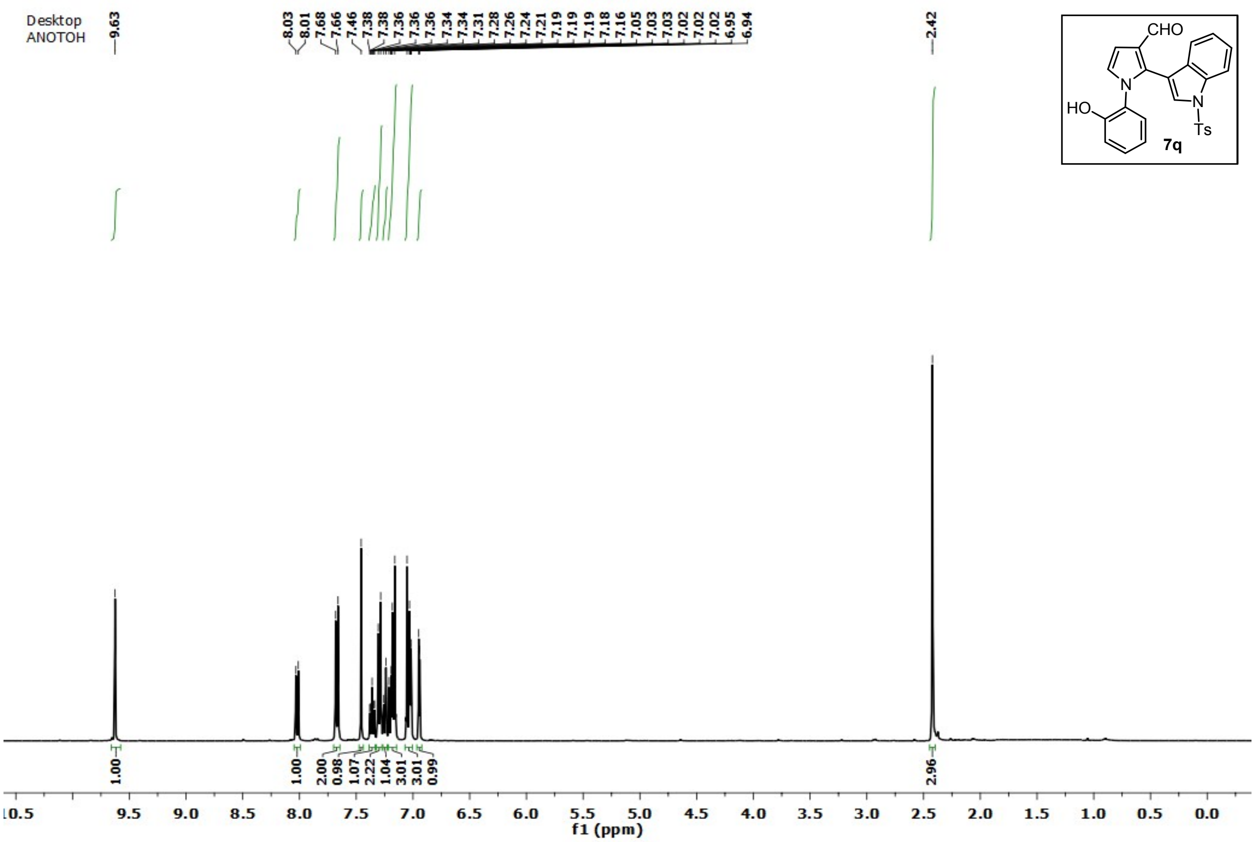
Desktop
ANOTOH
9.63
8.03
8.01
7.68
7.66
7.46
7.38
7.38
7.36
7.36
7.36
7.34
7.34
7.31
7.28
7.26
7.24
7.21
7.19
7.19
7.19
7.18
7.16
7.05
7.03
7.03
7.02
7.02
7.02
6.95
6.94
2.42
CHO
N
HO
N
Ts
7q
1.00
1.00
2.00
0.98
1.07
2.22
1.04
3.01
3.01
0.99
2.96
f1 (ppm)

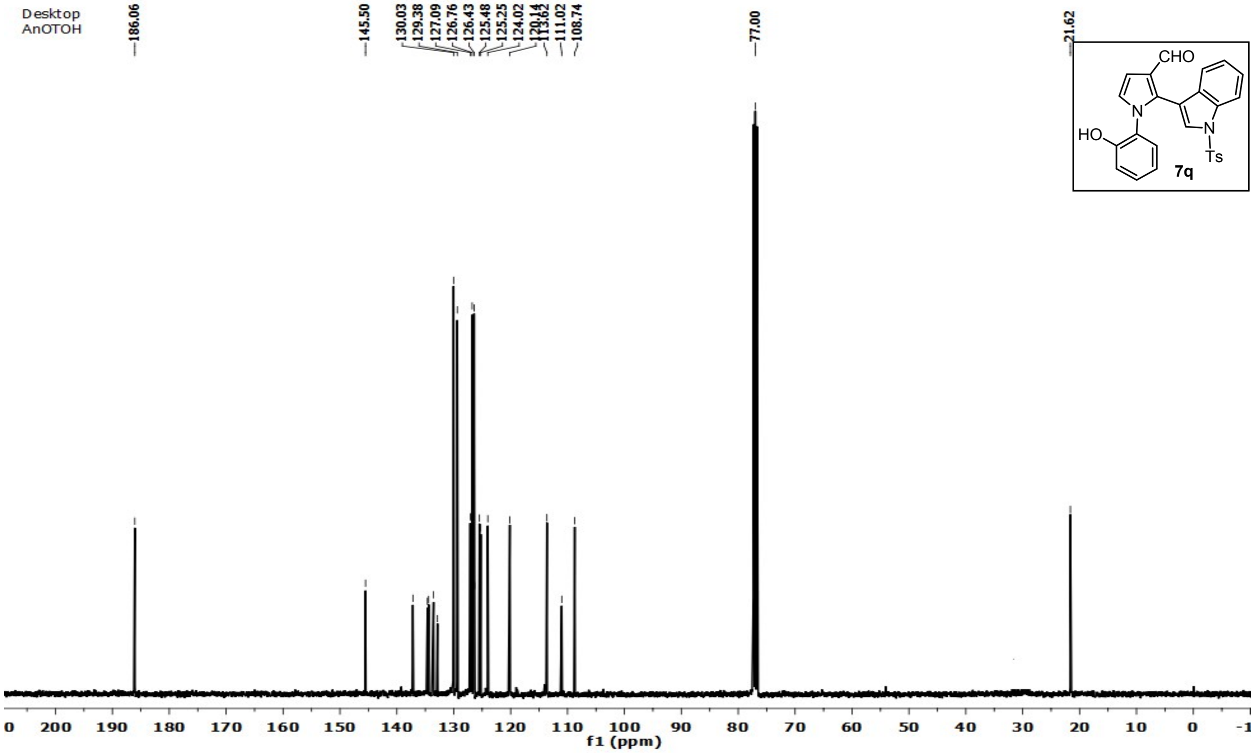
Desktop
AnOTOH
186.06
145.50
130.03
129.38
127.09
126.76
126.43
125.48
125.25
124.02
120.14
113.62
111.02
108.74
77.00
21.62
CHO
N
HO
N
Ts
7q
f1 (ppm)

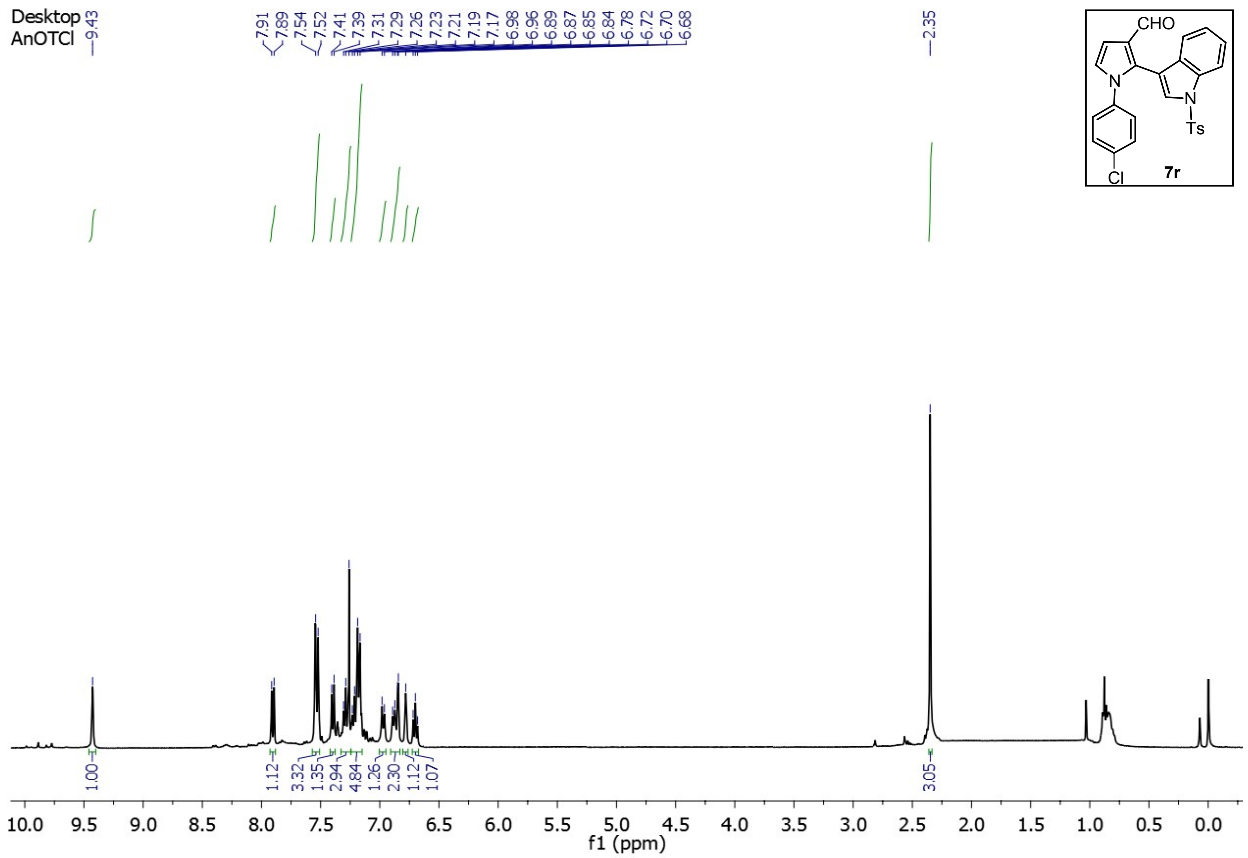
Desktop
AnOTCl
9.43
7.91
7.89
7.54
7.52
7.41
7.39
7.31
7.29
7.26
7.23
7.21
7.19
7.17
6.98
6.96
6.89
6.87
6.85
6.84
6.78
6.72
6.70
6.68
2.35
CHO
N
N
Ts
Cl
7r
1.00
1.12
3.32
1.35
2.94
4.84
1.26
2.30
1.12
1.07
3.05
f1 (ppm)

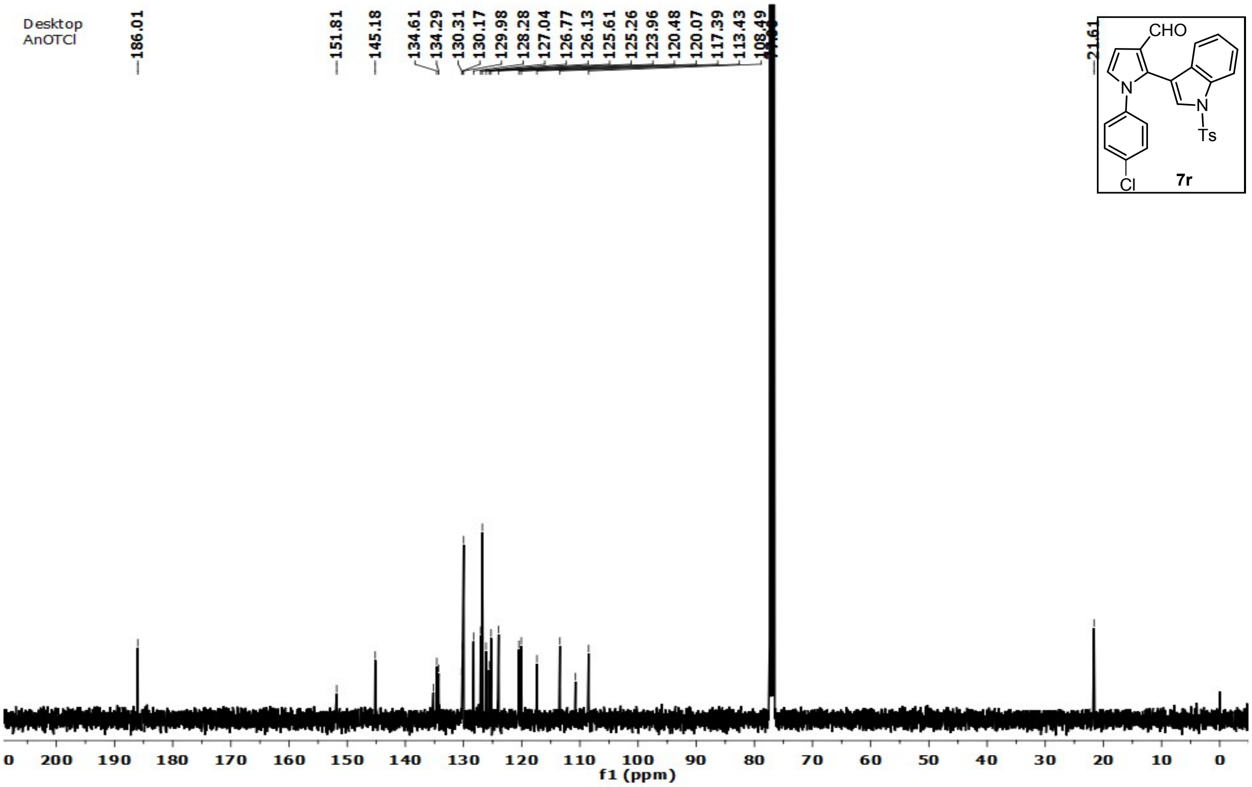
Desktop
AnOTCl
186.01
151.81
145.18
134.61
134.29
130.31
130.17
129.98
128.28
127.04
126.77
126.13
125.61
125.26
123.96
120.48
120.07
117.39
113.43
108.49
21.61
CHO
N
N
Ts
Cl
7r
f1 (ppm)

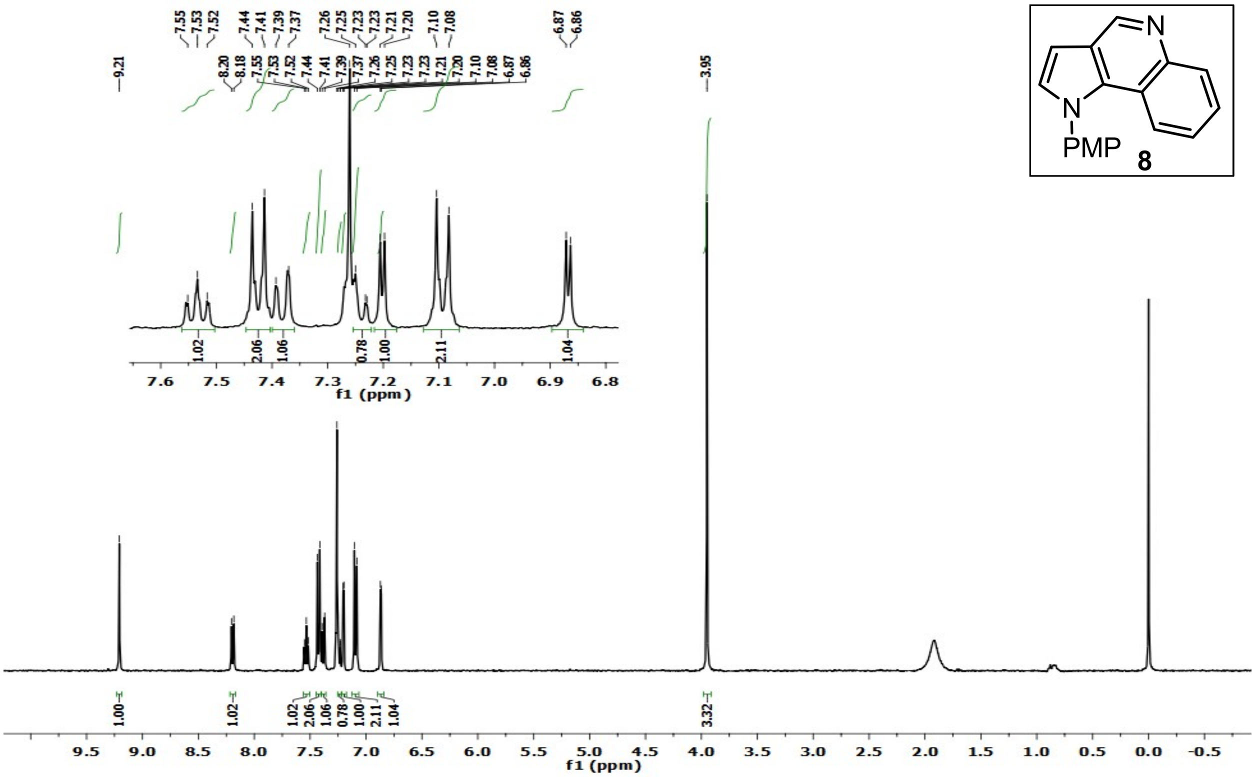

9.21
8.20
8.18
7.55
7.53
7.52
7.44
7.41
7.39
7.37
7.26
7.25
7.23
7.23
7.21
7.20
7.10
7.08
6.87
6.86
3.95
1.02
2.06
1.06
0.78
1.00
2.11
1.04
1.00
1.02
3.32
f1 (ppm)
N
N
PMP
8

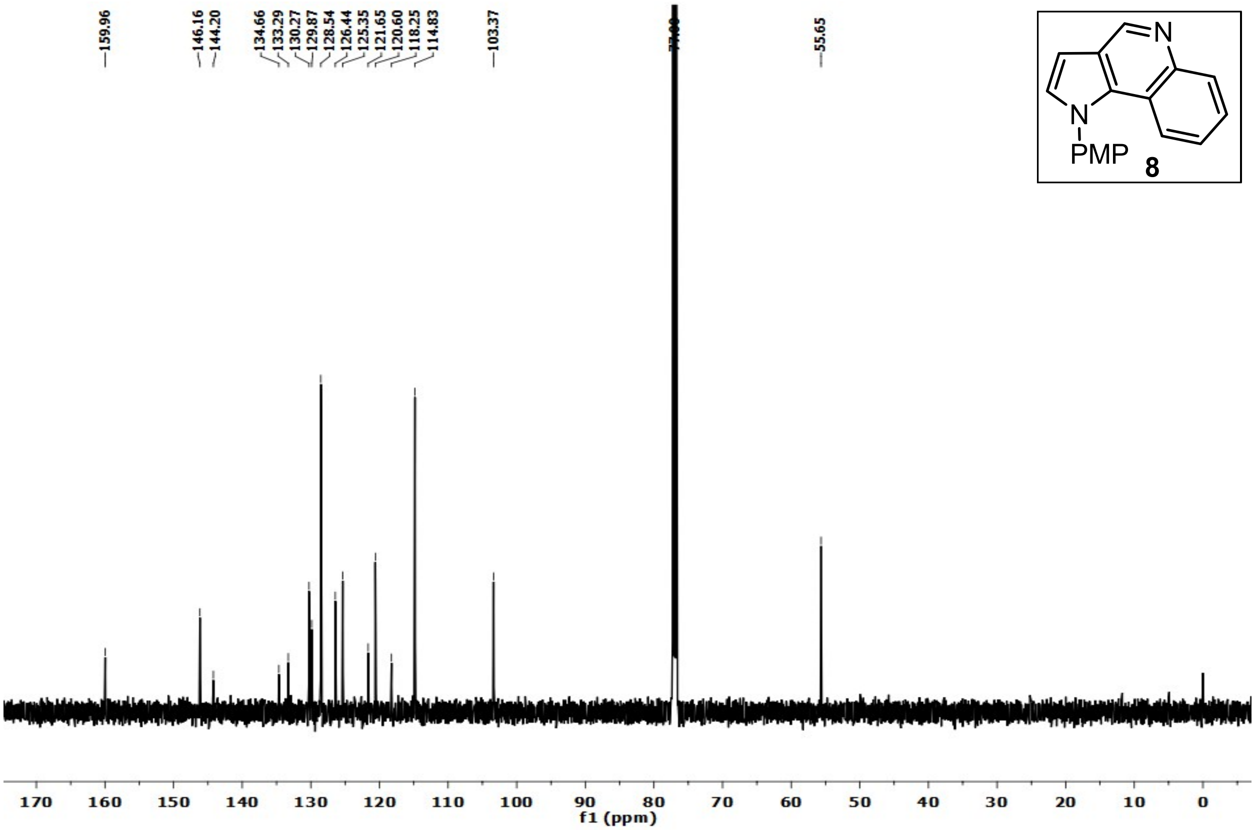

159.96
146.16
144.20
134.66
133.29
130.27
129.87
128.54
126.44
125.35
121.65
120.60
118.25
114.83
103.37
55.65
f1 (ppm)
N
N
PMP
8

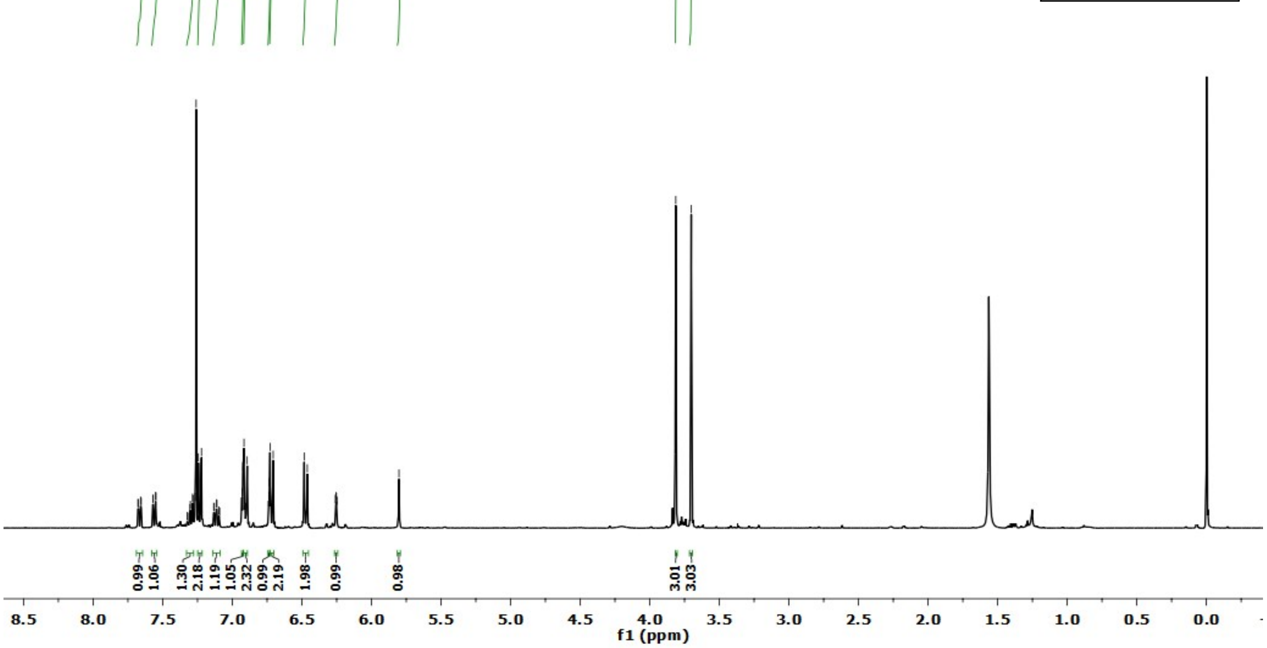

3.81
3.70
0.99
1.06
1.30
2.18
1.19
1.05
2.32
0.99
2.19
1.98
0.99
0.98
3.01
3.03
f1 (ppm)

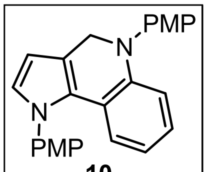

PMP
N
N
PMP

10

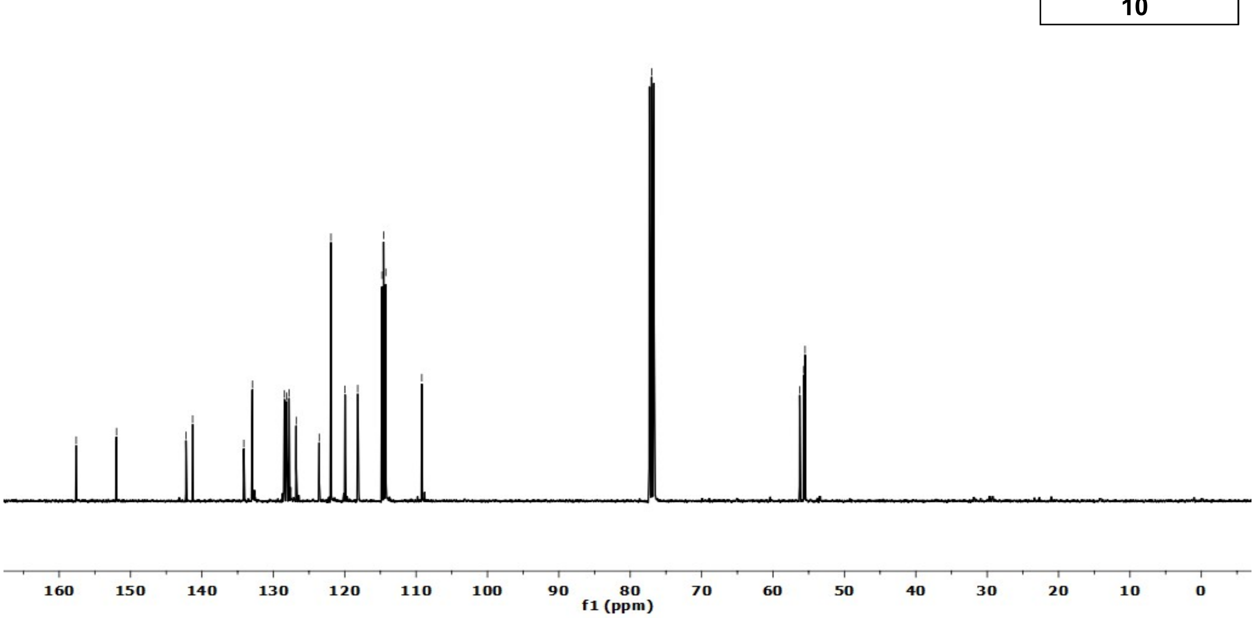

157.62
151.97
142.23
141.30
134.14
132.93
128.44
128.15
127.80
121.93
119.96
118.18
114.77
114.55
77.00
56.26
55.70
55.52
f1 (ppm)

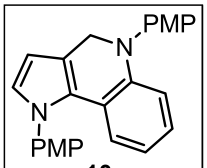

PMP
N
N
PMP

10

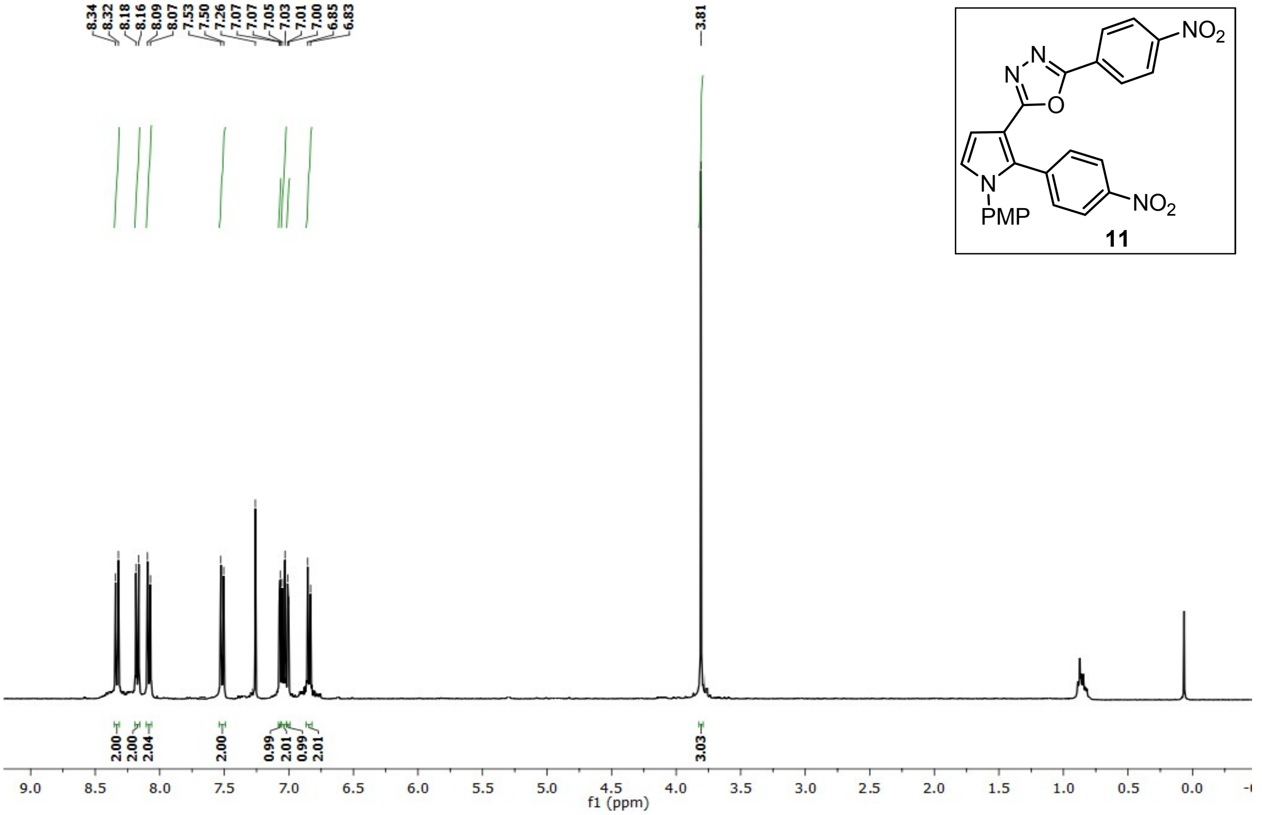
8.34
8.32
8.18
8.16
8.09
8.07
7.53
7.50
7.26
7.07
7.07
7.05
7.03
7.01
7.00
6.85
6.83
3.81
NO2
N
N
O
N
PMP
NO2
11
2.00
2.00
2.04
2.00
0.99
2.01
0.99
2.01
3.03
f1 (ppm)

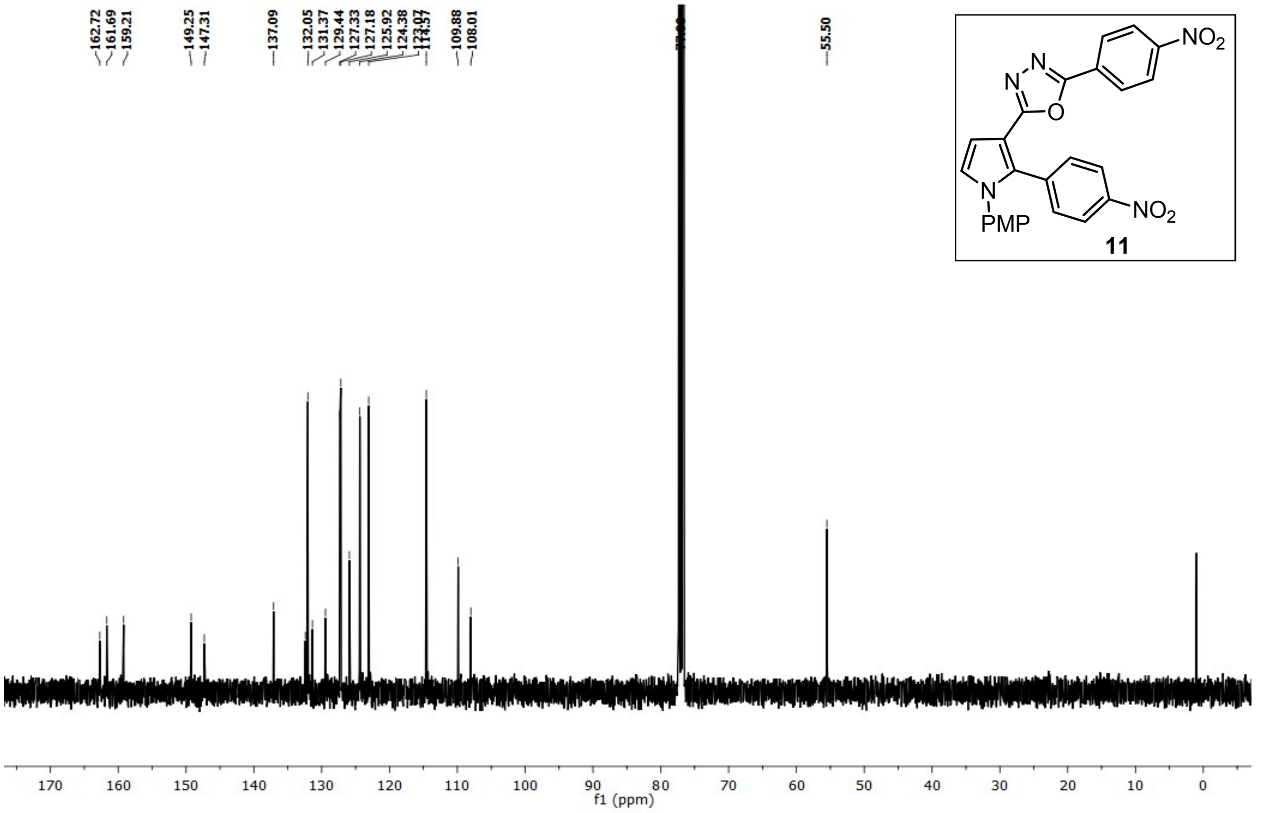
162.72
161.69
159.21
149.25
147.31
137.09
132.05
131.37
129.44
127.33
127.18
125.92
124.38
109.88
108.01
55.50
NO2
N
N
O
N
PMP
NO2
11
f1 (ppm)

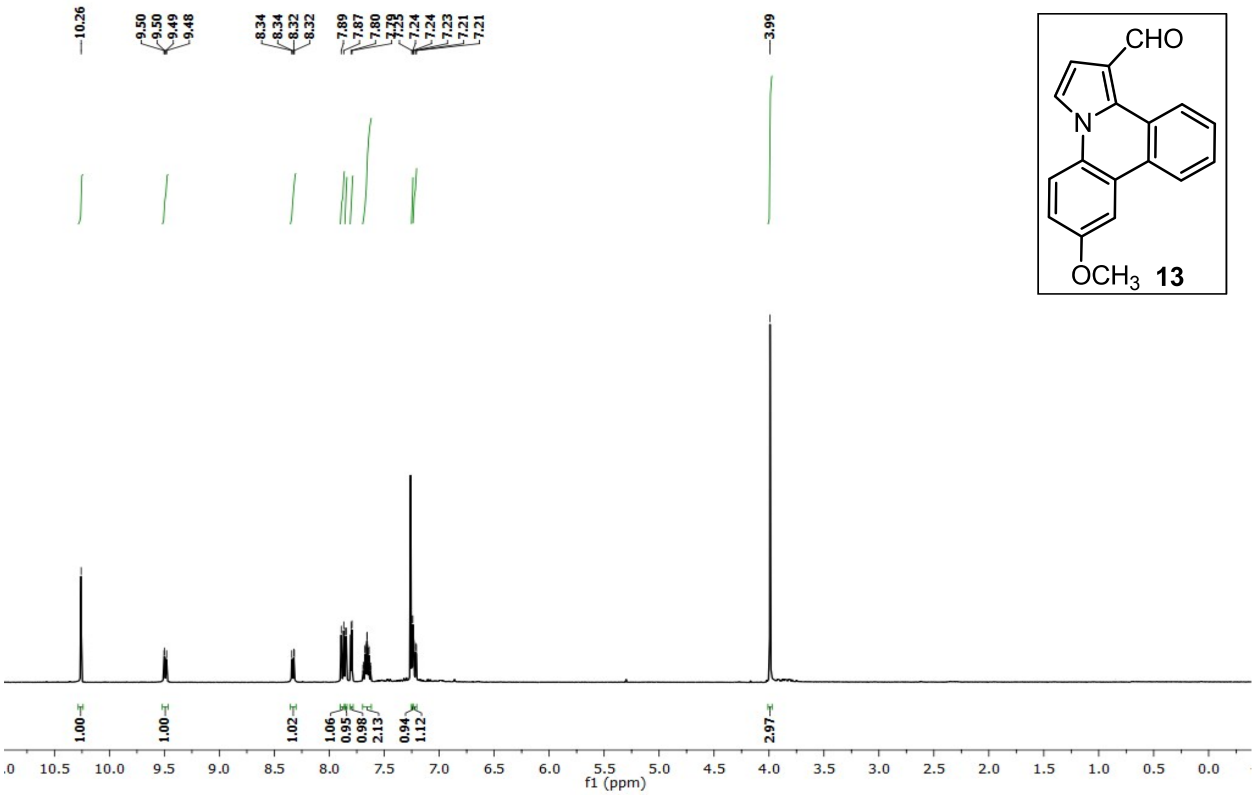
10.26
9.50
9.50
9.49
9.48
8.34
8.34
8.32
8.32
7.89
7.87
7.80
7.24
7.24
7.23
7.21
7.21
3.99
CHO
N
OCH3 13
1.00
1.00
1.02
1.06
0.95
0.98
2.13
0.94
1.12
2.97
f1 (ppm)

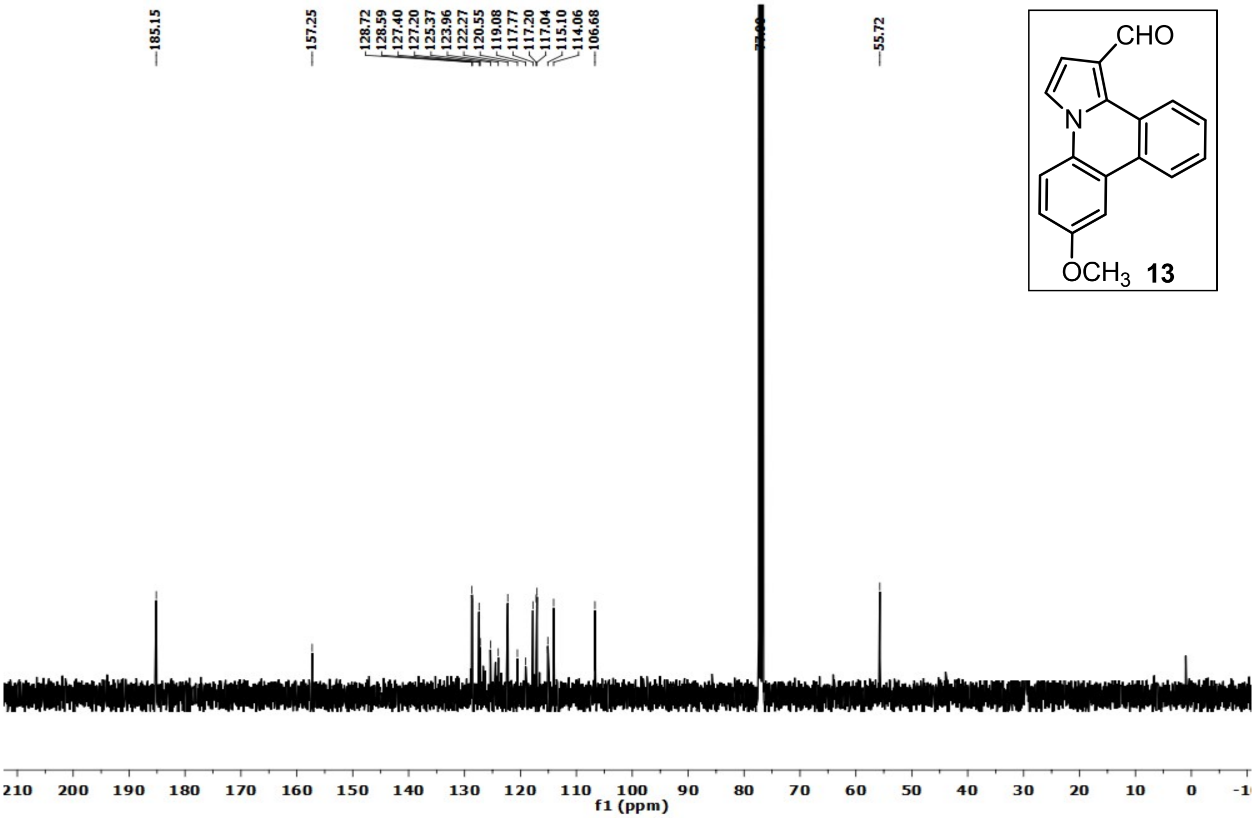
185.15
157.25
128.72
128.59
127.40
127.20
125.37
123.96
122.27
120.55
119.08
117.77
117.20
117.04
115.10
114.06
106.68
55.72
CHO
N
OCH3 13
f1 (ppm)

Crystal structure of 1-(4-methoxyphenyl)-2-(4-nitrophenyl)-1H-pyrrole-3-carbaldehyde (5c):

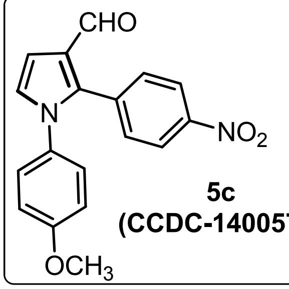

5c
(CCDC-1400572)

The compound 1-(4-methoxyphenyl)-2-(4-nitrophenyl)-1H-pyrrole-3-carbaldehyde, $C_{18}H_{14}N_2O_4$, crystallizes in the orthorhombic space group $P2_12_12_1$ with the unit-cell parameters: a= 8.0230(6), b= 10.5211(8), c= 18.4479(16) Å and Z = 4. The crystal structure was solved by direct methods using single-crystal X-ray diffraction data collected at room temperature and refined by full-matrix least-squares procedures to a final R-value of 0.0511 for 1184 observed reflections.

Crystal structure determination and refinement

X-ray intensity data of 4243 reflections (of which 1762 unique) were collected on *X'calibur* CCD area-detector diffractometer equipped with graphite monochromated MoKα radiation (λ = 0.71073 Å). The crystal used for data collection was of dimensions 0.30 x 0.20 x 0.20 mm. The cell dimensions were determined by least-squares fit of angular settings of 1096 reflections in the θ range 3.85 to 25.01°**.** The intensities were measured by ώ scan mode for θ ranges 3.84 to 26.00°. 1184 reflections were treated as observed ($I > 2\sigma(I)$). Data were corrected for Lorentz, polarisation and absorption factors. The structure was solved by direct methods using SHELXS97 [1]. All non-hydrogen atoms of the molecule were located in the best E-map. Full-matrix least-squares refinement was carried out using SHELXL97 [1]. The final refinement cycles converged to an R = 0.0511 and wR (F^2) = 0.0913 for the observed data. Residual electron densities ranged from $-0.194 < \Delta\rho < 0.143$ eÅ^{-3}. Atomic scattering factors were taken from International Tables for X-ray Crystallography (1992, Vol. C, Tables 4.2.6.8 and 6.1.1.4). The crystallographic data are summarized in Table 1. The geometry of the molecule was calculated using the WinGX [3], PARST [4] and PLATON [5] softwares.

Table 1: Crystal and experimental data

CCDC	1400572
Crystal description	white block

Crystal size	0.3 X 0.2 X 0.2 mm
Empirical formula	$C_{18}H_{14}N_2O_4$
Formula weight	322.31
Radiation, Wavelength	Mo *K*α, 0.71073 Å
Unit cell dimensions	a= 8.0230(6), b= 10.5211(8),<br>c= 18.4479(16) Å
Crystal system	orthorhombic
Space group	$P2_12_12_1$
Unit cell volume	1557.2(2)
No. of molecules per unit cell, Z	4
Temperature	293(2) K
Absorption coefficient	0.099 mm^{-1}
F(000)	672
Scan mode	ω scan
θ range for entire data collection	$3.84 < \theta < 26.00$
Range of indices	h= -8 to 9, k= -5 to 12, l= -13 to 22
Reflections collected / unique	4243/ 1762
Reflections observed (I > 2σ(I))	1184
R_{int}	0.0390
R_{sigma}	0.0639
Structure determination	Direct methods
Refinement	Full-matrix least-squares on F^2
No. of parameters refined	218
Final R	0.0511
$wR(F^2)$	0.0913
Weight	$1/[\sigma^2(F_o^2)+(0.0412P)^2+0.0000P]$<br>Where $P=[F_o^2+2F_c^2]/3$
Goodness-of-fit	1.032

Final residual electron density	$-0.194<\Delta\rho < 0.143 e\AA^{-3}$
Measurement	*X'calibur system-Oxford diffraction make, U.K*
Software for structure solution:	SHELXS97 (Sheldrick, 2008)
Software for refinement:	SHELXL97 (Sheldrick, 2008)
Software for molecular plotting:	ORTEP-3 (Farrugia, 1997) PLATON
Software for geometrical calculation	PLATON (Spek, 2009) PARST (Nardelli, 1995)

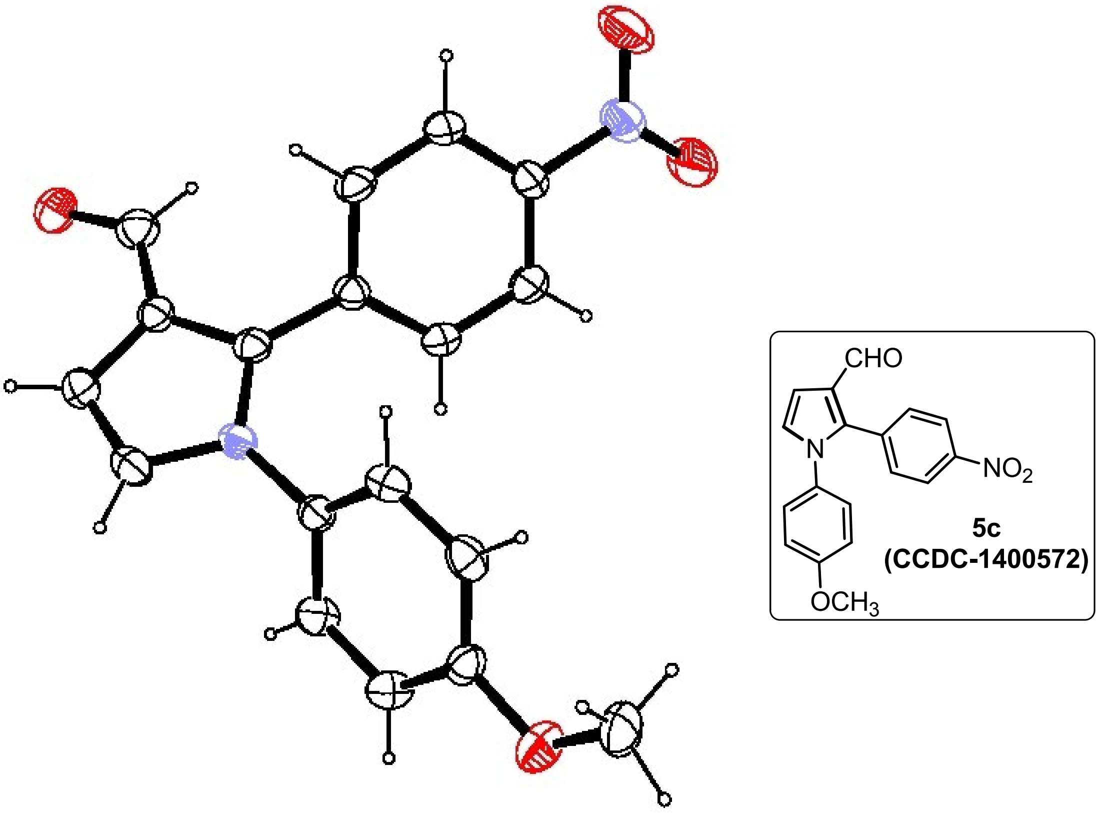

Figure 1 *ORTEP* view of the molecule with displacement ellipsoids drawn at 40%.

H atoms are shown as small spheres of arbitrary radii.

2-(5-chloro-1-tosyl-1*H*-indol-3-yl)-1-(4-methoxyphenyl)-1*H*-pyrrole-3-carbaldehyde (7e):

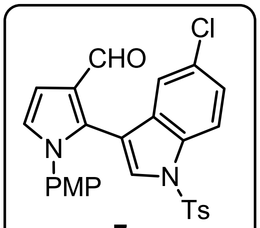

7e
(CCDC-1471798)

The compound 2-(5-chloro-1-tosyl-1*H*-indol-3-yl)-1-(4-methoxyphenyl)-1*H*-pyrrole-3-carbaldehyde, $C_{27}H_{21}ClN_2O_4S$ crystallizes in the monoclinic space group $P2_1/n$ with the following unit cell parameters: *a*=9.9462 (7), *b*= 15.4122 (9), *c*= 15.2477 (11) Å, β=96.432 (7)° and Z=4. The crystal structure was solved by direct methods and refined by full matrix least-squares procedure to a final R value of 0.051 for 2862 observed reflections.

Crystal structure determination and refinement

X-ray intensity data consisting of 10516 reflections were collected at 293(2) K and 4546 reflections were found unique. A crystal of dimensions 0.30 x 0.20 x 0.20 mm was used for data collection on *X*'calibur CCD area-detector diffractometer, equipped with graphite monochromated Mo*Kα* radiation (λ=0.71073 Å) [6]. The intensities were measured by ω scan mode for θ ranges 3.7 to 26.0°. A total number of 2862 reflections were treated as observed [$I > 2\sigma(I)$] . Data were corrected for Lorentz-polarization and absorption factors. The structure was solved by direct methods using SHELXS97 [1]. All non-hydrogen atoms of the molecule were located in the best E-map. All the hydrogen atoms were geometrically fixed and allowed to ride on the corresponding non-H atoms with C-H= 0.93-0.94 Å and U_{iso} = 1.2 U_{eq}(C), except for the methyl groups where $U_{iso}(H) = 1.5U_{eq}(C)$. The refinement cycles converged the structure to a final *R*- factor of 0.0513 (w*R* (F^2) = 0.1128) for the 2862 observed reflections. Residual electron densities ranges from -0.271 to 0.270 $eÅ^{-3}$. Atomic scattering factors were taken from International Tables for X-ray Crystallography (1992, Vol. C, Tables 4.2.6.8 and 6.1.1.4) [7].

An ORTEP [8] view of the compound with atomic labelling scheme is shown in Figure 1. The geometry of the molecule was calculated using the PARST [4] and PLATON [5] software. Crystal data, along with data collection and structure refinement details are summarized in Table 1. Selected bond lengths, bond angles and torsion angles are given in Table 2. The geometry of intra and inter molecular interactions is given in Table 2.

Table 2: Crystal and experimental Data.

Chemical formula	$C_{26}H_{19}ClN_2O_3S$
CCDC No.	1471798
M_r	474.94
Crystal system, space group	Monoclinic, $p2_1/n$
Temperature (K)	293
a, b, c (Å)	9.9462 (7), 15.4122 (9), 15.2477 (11)
β (°)	96.432 (7)
V (Å^3)	2322.7 (3)
Z	4
Radiation type	Mo $K\alpha$
μ (mm^{-1})	0.29
Crystal size (mm)	0.30 × 0.20 × 0.20
T_{min}, T_{max}	0.625, 1.000
No. of measured, independent and observed [$I > 2\sigma(I)$] reflections	10516, 4546, 2862
R_{int}	0.041
$(\sin \theta/\lambda)_{max}$ (Å^{-1})	0.617
$R[F^2 > 2\sigma(F^2)]$, $wR(F^2)$, S	0.051, 0.113, 1.02
No. of reflections	4546
No. of parameters	311
No. of restraints	0
$\Delta\rho_{max}$, $\Delta\rho_{min}$ (e Å^{-3})	0.28, −0.27

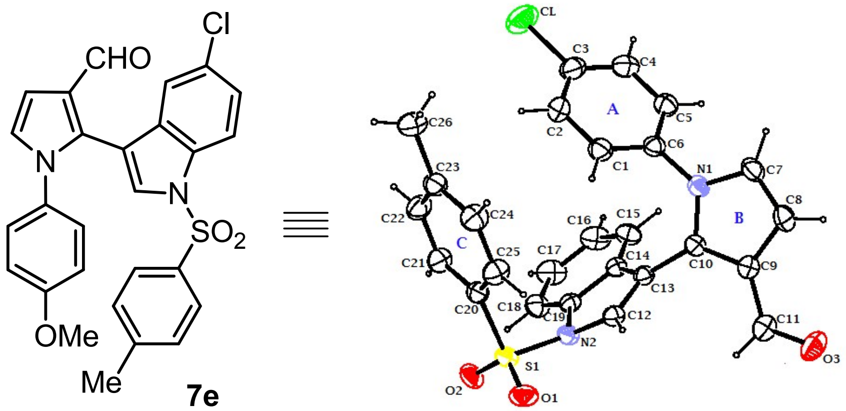

7e
(CCDC-1471798)

Figure 2: ORTEP view of the molecule with displacement ellipsoids drawn at the 40% probability level. H atoms are shown as small sphere of arbitrary radii.

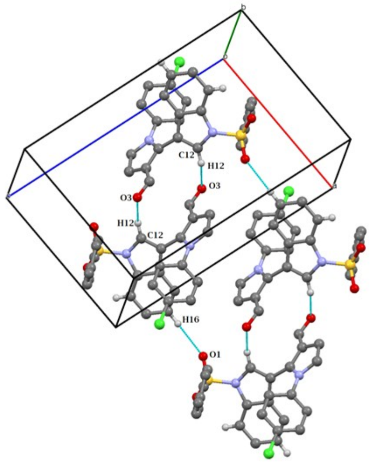

Figure 3: A dimer formed by intermolecular C-H….O hydrogen bonds

References:

1. Sheldrick GM (2008) Acta Cryst A64: 112
2. Farrugia LJ (1997) J Appl Cryst 30:565
3. Farrugia LJ (1999) J Appl Cryst 32:837
4. Nardelli M (1995) J Appl Cryst 28:659
5. Spek AL (2009) Acta Cryst D65: 148–155.
6. Oxford Diffraction (2010).CrysAlis PRO. Oxford Diffraction Ltd, Yarnton, England.
7. International Tables for X-ray Crystallography, Tables 4.2.6.8 and 6.1.1.4, A J C Wilson, Editor, Boston, Kluwer Academic Press, Vol. C, 1992.
8. Farrugia, L. J. (2012).J. Appl. Cryst.45, 849–854.
